# Global, Regional and National Burden of Cancers Attributable to High Fasting Plasma Glucose in 204 Countries and Territories, 1990-2019

**DOI:** 10.3389/fendo.2022.879890

**Published:** 2022-07-19

**Authors:** Saeid Safiri, Seyed Aria Nejadghaderi, Nahid Karamzad, Jay S. Kaufman, Kristin Carson-Chahhoud, Nicola Luigi Bragazzi, Mark J. M. Sullman, Mohammad Reza Beyranvand, Mohammad Ali Mansournia, Amir Almasi-Hashiani, Gary S. Collins, Ali-Asghar Kolahi

**Affiliations:** ^1^ Research Center for Integrative Medicine in Aging, Aging Research Institute, Tabriz University of Medical Sciences, Tabriz, Iran; ^2^ Social Determinants of Health Research Center, Department of Community Medicine, Faculty of Medicine, Tabriz University of Medical Sciences, Tabriz, Iran; ^3^ Systematic Review and Meta‐analysis Expert Group (SRMEG), Universal Scientific Education and Research Network (USERN), Tehran, Iran; ^4^ Social Determinants of Health Research Center, Shahid Beheshti University of Medical Sciences, Tehran, Iran; ^5^ Nutrition Research Center, Department of Biochemistry and Diet Therapy, School of Nutrition and Food Sciences, Tabriz University of Medical Sciences, Tabriz, Iran; ^6^ Department of Epidemiology, Biostatistics and Occupational Health, Faculty of Medicine, McGill University, Montreal, Quebec, Canada; ^7^ Australian Centre for Precision Health, Allied Health and Human Performance, University of South Australia, Adelaide, SA, Australia; ^8^ School of Medicine, The University of Adelaide, Adelaide, SA, Australia; ^9^ Centre for Disease Modelling, York University, Toronto, ON, Canada; ^10^ Department of Life and Health Sciences, University of Nicosia, Nicosia, Cyprus; ^11^ Department of Social Sciences, University of Nicosia, Nicosia, Cyprus; ^12^ Department of Epidemiology and Biostatistics, School of Public Health, Tehran University of Medical Sciences, Tehran, Iran; ^13^ Department of Epidemiology, School of Health, Arak University of Medical Sciences, Arak, Iran; ^14^ Centre for Statistics in Medicine, NDORMS, Botnar Research Centre, University of Oxford, Oxford, United Kingdom; ^15^ NIHR Oxford Biomedical Research Centre, Oxford University Hospitals NHS Foundation Trust, Oxford, United Kingdom

**Keywords:** diabetes mellitus, neoplasms, incidence, mortality, epidemiology

## Abstract

**Background:**

To report the burden of cancers attributable to high fasting plasma glucose (HFPG) by sex, age, location, cancer type and Socio-demographic Index (SDI) over the period 1990 to 2019 for 204 countries and territories.

**Methods:**

Using the Comparative Risk Assessment approach of Global Burden of Disease (GBD) study 2019, the burden of cancers attributable to HFPG was reported in 1990 and 2019.

**Results:**

Globally, in 2019 there were an estimated 419.3 thousand cancer deaths (95% UI: 115.7 to 848.5) and 8.6 million cancer DALYs (2.4 to 17.6) attributable to HFPG. By sex, 4.6 (1.1 to 9.9) and 4.0 (1.1 to 8.4) million global cancer DALYs were attributable to HFPG in men and women, respectively. The global age-standardized death and DALY rates of cancers attributable to HFPG (per 100,000) have increased by 27.8% (20.5 to 38.7%) and 24.5% (16.4 to 35.6%), respectively, since 1990. High-income North America (9.5 [2.7 to 18.8]) and Eastern Sub-Saharan Africa (2.0 [0.5 to 4.2]) had the highest and lowest regional age-standardized death rates, respectively, for cancers attributable to HFPG. In 2019, the global number of attributable cancer DALYs were highest in 65-69 age group. Moreover, there was an overall positive association between SDI and the regional age-standardized DALY rate for HFPG-attributable cancers.

**Conclusions:**

HFPG was associated with more burden in 2019. Preventive programs for diabetes and screening of individuals with diabetes for cancers, especially in older males living in developed countries, are required to arrest the large increases in HFPG-attributable cancers.

## Introduction

In 2019, high fasting plasma glucose (HFPG), which is defined as fasting plasma glucose above 86.4-97.2 mg/dl, was globally responsible for 11.3% of all age-standardized deaths and 6.4% of the disability-adjusted life-years (DALYs) from all causes (1, 2). In addition, it is a prognostic factor for several infectious diseases (3). Diabetes, which is one of the most common manifestations of HFPG, imposes a substantial mortality, morbidity and economic burden upon society (4).

In 2017, the global age-standardized death and DALY rates of diabetes were 17.5 and 839.0 per 100,000, respectively, which had increased by more than 100% since 1990 (5). Diabetes was more prevalent in individuals aged over 60 years of age and in low- and middle-income countries, while there were no significant sex differences (4). Moreover, it was estimated that the prevalence of impaired glucose tolerance in adults aged 18-99 years will increase from 374 million in 2017 to 587 million by 2045 (4). Globally, diabetes was also estimated to cost 850 billion USD in 2017, with an expected increase of 958 billion by 2045 (4).

Studies have shown associations between HFPG/diabetes, and several different types of cancers, such as colorectal and breast cancer (6, 7) and this suggests that burden of cancers attributable to HFPG may be of public health interest. One study reported the burden of all non-communicable diseases (NCDs) attributable to HFPG at the global, regional and national level in 2017 (8), but there was no specific report published on cancers. Also, a few studies have reported the burden of cancers attributable to HFPG or diabetes and these also require updating (9, 10). To the best of our knowledge, no up-to-date study has comprehensively reported the level and trend of cancers attributable to HFPG across the globe. Establishing a scientifically rigorous evidence-based snapshot of the disease burden across regions and nations, may help spur action by governments and policy makers where the burden is identified as being high and/or increasing. This comparative analysis also provides benchmarking across regions and nations that policy makers can use to direct further exploration for areas doing well and those doing poorly.

Therefore, we aimed to report the HFPG-attributable burden of cancers in 204 countries and territories from 1990 to 2019 by age, sex, level of development (i.e., Socio-demographic index - SDI) and cancer type using the most comprehensive and up-to-date estimates from the Global Burden of Diseases (GBD) 2019 study. This information is necessary to underpin recommendations by government representatives, researchers, and policy makers that can be used to inform intervention development, resource allocation and priority agenda setting. These activities are needed to create a coordinated evidence-based effort that can be applied broadly across priority settings to reduce the burden of HFPG and diabetes.

## Material and Methods

### Overview

The Global Burden of Disease (GBD) project, which is conducted by the Institute for Health Metrics and Evaluation (IHME), examines the levels and trends of communicable diseases, non-communicable diseases and injuries across the world. In the most recent update of the project, GBD 2019, 87 risk factors were studied in 204 countries and territories, 7 super-regions and 21 regions, from 1990 to 2019 (11). The Comparative Risk Assessment approach, as used in GBD 2019, was used to report the burden of cancers attributable to HFPG. The GBD 2019 methodology for estimating the burden of diseases, injuries and risk factors are detailed elsewhere (11, 12). Further information on non-fatal event estimates can be found at https://vizhub.healthdata.org/gbd-compare/ and http://ghdx.healthdata.org/gbd-results-tool).

### Case Definition and Data Sources

High fasting plasma glucose (FPG) was measured as the mean FPG in a population, where FPG is a continuous exposure in units of mmol/L. As FPG runs along a continuum, IHME defined high FPG as any level above the theoretical minimum-risk exposure level (TMREL), which is 4.8-5.4 mmol/L or 86.4–97.2 mg/dL.

The IHME conducted a systematic review for FPG and diabetes in GBD 2019. All available research on FPG and the prevalence of diabetes were included in the FPG model. The search terms used for diabetes mellitus and FPG are reported elsewhere (11). A total of 717 studies were identified, but only 36 were retained (11). The following sources were used as data inputs: a) estimates of the mean FPG in a representative population, b) individual-level data of FPG measured using surveys and c) estimates of the prevalence of diabetes in a representative population. Those sources which did not report mean FPG or the prevalence of diabetes were excluded from the study, but when a study reported both mean FPG and the prevalence of diabetes, the mean FPG was used. Individual-level data supersede any data described in a study and these were aggregated to produce estimates for each 5-year age group, sex, location, and year. A total of 549 data sources were used in the modelling process, with data on the exposure to HFPG and the relative risk coming from 127 countries and territories (11).

### Data Process and Modelling

The data were subjected to several processing steps to address sampling and measurement inconsistencies and to improve comparability. Those estimates in a sex- and age- group with a sample size < 30 were considered to be too small and were collapsed into the next age group in the same study in order to increase the sample size and to avoid probable biases. If the sample size of the entire study was < 30 and did not include a population-weight, the study was not included in the modelling process. The mean FPG was estimated from the prevalence of diabetes using an ensemble distribution developed by GBD. In addition, it was checked that the reported prevalence of diabetes was based upon the standard definition, FPG >126 mg/dL (7 mmol/L) or on receiving treatment for diabetes (11, 13).

The exposure estimates were produced for each year, from 1990 to 2019, for each location, sex, and 5-year age group (starting from 25 years old). The Spatio-Temporal Gaussian Process Regression (ST-GPR) framework was used to model the mean FPG at the location-, year-, age-, and sex- level (11). FPG was often reported in studies investigating the prevalence of diabetes mellitus. In these studies, the case definition of diabetes may include a glucose test and/or questions about whether they were receiving treatment for diabetes. Those with a history of diabetes treatment may have been excluded from the FPG test, so the mean FPG in these surveys would not represent the mean for the entire population. In this case, the prevalence of diabetes was calculated using a definition of FPG>126 mg/dL (7mmol/L), then it was adjusted to the reference case definition, and the mean FPG was estimated.

### Data on Estimated Relative Risk

The TMREL for FPG (4.8-5.4 mmol/L or 86.4–97.2 mg/dL) was calculated by taking the person-year weighted average of the levels of FPG that were associated with the lowest risk of mortality in the pooled analyses of prospective cohort studies. This level is lower than the current cutoff point for the diagnosis of diabetes (≥7.0 mmol/L or 126 mg/dL) (11). Although there is a high degree of over-lap between diabetes and HPFG, there are also some differences. People with pre-diabetes have HFPG (as a risk factor), but do not have diabetes, while some people with controlled diabetes might be at lower risk, based on their plasma glucose levels. Relative risks (RR) were obtained from a dose-response meta-analysis of prospective cohort studies. Morbidity and mortality that were directly caused by diabetes type 1 and diabetes type 2 were considered directly attributable to FPG. The above-mentioned systematic review found the following cancers to be associated with HFPG (11): tracheal, bronchus and lung cancer (14), breast cancer (15), bladder cancer (16), ovarian cancer (17), pancreatic cancer (18), liver cancer (19), and colon and rectum cancer (20).

### Estimation of the Proportion of Cancers Attributable to High Fasting Plasma Glucose

The estimated relative risk (RR) was used and the level of risk factors that were associated with the lower risk were measured using the theoretical minimum risk exposure level (TMREL) in GBD 2019 (11). The population-attributable fractions (PAF) were then computed to quantify the burden of cancers related to HFPG by country, age, sex, and year. In the present study, the International Classification of Diseases (ICD) version 10 codes (C00-C96) were used to indicate malignant neoplasms or cancers (11). More detailed information on the methods for calculating the PAFs for HFPG (continuous risk factor) and diabetes (categorical risk factor) are reported elsewhere (11).

The deaths and DALYs attributable to HFPG were estimated by multiplying the PAFs by the total number of deaths or DALYs reported in GBD 2019, for each country, age group, sex, year, and type of cancer. The detailed methods for calculating the total deaths and DALYs, due to cancers, can be found elsewhere (11). The estimates were all reported in terms of numbers, proportions (PAFs) and age-standardized rates per 100,000, along with their corresponding 95% uncertainty intervals (UIs). The current study also investigated the association that SDI had with the burden of cancers attributable to HFPG. SDI is a composite measure of lag-distributed income per capita, years of schooling in the population 15 years of age and above, and total fertility rate under the age of 25. The SDI ranges from 0 (least developed) to 1 (most developed).

## Results

### Global Level

In 2019, globally there were an estimated 419.3 thousand cancer deaths (95% UI: 115.7 to 848.5) attributable to HFPG, which represents 4.2% (1.1 to 8.4) of all cancer-related deaths (**Table 1**), with 224.8 thousand (55.5 to 481.5) of these deaths being among males and 194.5 thousand (53.4 to 409.5) among females (**Table S1**). The age-standardized death rate of cancers attributable to HFPG in 2019 (5.3 [1.5 to 10.6] per 100,000) was 27.8% (20.5 to 38.7) higher than in 1990 (4.1 [1.1 to 8.5]) (**Table S2**). In addition, in 2019 HFPG contributed 8.6 million DALYs (2.4 to 17.6), which represents 3.4% (0.9 to 6.9) of all cancer-related DALYs, with 4.6 (1.1 to 9.9) million DALYs in men and 4.0 (1.1 to 8.4) million DALYs in women (**Table S3**). Between 1990 and 2019, the age-standardized DALY rate of cancers attributable to HFPG (per 100,000) increased from 83.7 (21.8 to 174.2) to 104.2 (28.7 to 212.9), a relative increase of 24.5% (16.4 to 35.6) from the 1990 level (**Table S4**).

**Table 1:** Cancer deaths and DALYs attributable to high fasting plasma glucose in 2019, by GBD region (Generated from data available from http://ghdx.healthdata.org/gbd-results-tool) .

	Deaths (95% UI)				DALY (95% UI)			
	Counts (2019)	PAF(2019)	ASRs(2019)	% change in ASRs 1990-2019	Counts (2019)	PAF(2019)	ASRs(2019)	% change in ASRs 1990-2019
Global	419338 (115729, 848484)	4.2 (1.1, 8.4)	5.3 (1.5, 10.6)	27.8 (20.5, 38.7)	8580783 (2357076, 17568241)	3.4 (0.9, 6.9)	104.2 (28.7, 212.9)	24.5 (16.4, 35.6)
High‐income Asia‐Pacific	20869 (5468, 43482)	3.8 (1, 7.7)	4 (1.1, 8.4)	5.8 (-1.6, 12.5)	338416 (89233, 701103)	3.4 (0.9, 7)	75.9 (19.9, 157.3)	1 (-4.6, 7)
High‐income North America	62291 (17929, 122539)	7.2 (2.1, 14.1)	9.5 (2.7, 18.8)	14.6 (7.9, 27)	1203538 (343541, 2366869)	6.5 (1.9, 12.8)	191.4 (54.4, 376.8)	4.8 (-1.2, 16.1)
Western Europe	74827 (21400, 148875)	5.8 (1.7, 11.5)	7.5 (2.1, 15.1)	19.6 (13.1, 31.4)	1293209 (369006, 2586197)	5.2 (1.4, 10.4)	145.2 (41, 291.3)	15.4 (9, 27.1)
Australasia	2573 (698, 5274)	4 (1.1, 8.2)	4.8 (1.3, 9.9)	18 (8.3, 33.5)	44694 (12088, 91895)	3.4 (0.9, 7)	89.4 (24.2, 184.5)	14.3 (5.2, 29.1)
Andean Latin America	1694 (462, 3598)	2.6 (0.7, 5.3)	3.1 (0.9, 6.7)	82.6 (51.6, 126)	32792 (8962, 70558)	2 (0.5, 4.1)	59.5 (16.2, 127.7)	77.7 (45.4, 121.7)
Tropical Latin America	9761 (2712, 19987)	3.6 (1, 7.2)	4.2 (1.2, 8.5)	11.5 (5, 19.9)	203917 (56014, 420429)	2.9 (0.8, 5.9)	84 (23.2, 172.7)	8.7 (2.4, 17.4)
Central Latin America	10949 (3168, 22280)	4.7 (1.4, 9.2)	4.8 (1.4, 9.7)	14.1 (-0.7, 32)	236568 (67139, 491403)	3.9 (1.1, 7.6)	100.2 (28.5, 207.5)	20.5 (4, 41)
Southern Latin America	6034 (1689, 12145)	4.8 (1.3, 9.5)	7.1 (2, 14.3)	53.7 (42.2, 73.1)	116880 (32392, 236478)	4.1 (1.1, 8.2)	139.7 (38.6, 283.2)	49 (37.4, 68.6)
Caribbean	3546 (1015, 7217)	5.1 (1.5, 10)	6.9 (2, 14)	43.5 (25, 69.3)	72360 (20252, 150189)	4.2 (1.2, 8.3)	139.5 (39, 289.3)	46.1 (27.1, 73.5)
Central Europe	20676 (5721, 42693)	6 (1.7, 12)	9.3 (2.6, 19.2)	52.5 (33.6, 75.8)	421066 (115565, 878373)	5.4 (1.5, 10.9)	196.5 (53.6, 413.1)	47.3 (28.6, 71.4)
Eastern Europe	12022 (3190, 25535)	2.7 (0.7, 5.7)	3.4 (0.9, 7.2)	21.7 (9.4, 37.6)	263707 (69152, 559496)	2.4 (0.6, 5)	75.9 (19.9, 161.4)	14.2 (2.6, 30.5)
Central Asia	3000 (802, 6151)	3.4 (0.9, 6.9)	4.4 (1.2, 9.1)	77.7 (59, 108.1)	75461 (19933, 156357)	2.8 (0.8, 5.7)	98.8 (26.4, 203.1)	59 (41.3, 86.6)
North Africa and Middle East	19755 (5517, 40246)	4.7 (1.3, 9.3)	5.1 (1.4, 10.3)	93.9 (68.3, 133.3)	462151 (127349, 959468)	3.8 (1.1, 7.6)	107 (29.8, 220.8)	90.1 (64.4, 127.8)
South Asia	38990 (10922, 80737)	3.1 (0.9, 6.3)	3 (0.8, 6.2)	95.6 (66.1, 137.3)	931637 (256236, 1937835)	2.5 (0.7, 5.1)	65.2 (18, 135.2)	100.5 (69.7, 142.2)
Southeast Asia	26110 (7020, 55023)	3.9 (1.1, 8)	4.8 (1.3, 10.1)	85.4 (58.3, 119.3)	581360 (154813, 1235009)	3.1 (0.8, 6.2)	96.7 (26.2, 204.4)	78 (52.7, 109.7)
East Asia	94519 (24177, 204701)	3.4 (0.9, 7.1)	4.7 (1.2, 10.3)	45.8 (21.2, 76.6)	2034530 (517721, 4451841)	2.9 (0.7, 6.2)	95.7 (24.4, 209.1)	37 (12.6, 69.1)
Oceania	486 (131, 1052)	5.5 (1.6, 11.2)	7.4 (2.1, 15.9)	77.4 (47.4, 121)	13829 (3717, 30700)	4.6 (1.3, 9.4)	177.4 (48, 384.6)	83.6 (50, 131.4)
Western sub‐Saharan Africa	4174 (1131, 8853)	2.1 (0.6, 4.4)	2.7 (0.7, 5.7)	88.1 (60.1, 122.8)	92015 (24253, 198384)	1.5 (0.4, 3.1)	52.5 (14.2, 112.3)	90.2 (60, 125.5)
Eastern sub‐Saharan Africa	2682 (707, 5689)	1.4 (0.4, 2.9)	2 (0.5, 4.2)	49.5 (32.8, 71)	62092 (16026, 134535)	0.9 (0.3, 2)	39.7 (10.4, 84.8)	49 (30.9, 72.2)
Central sub‐Saharan Africa	1492 (370, 3423)	2.6 (0.7, 5.7)	3.4 (0.8, 7.6)	29.7 (3.4, 66.4)	37203 (8966, 86838)	2 (0.5, 4.3)	70.9 (17.5, 162.3)	32.1 (2.4, 71.6)
Southern sub‐Saharan Africa	2886 (811, 5789)	3.9 (1.1, 7.8)	5.8 (1.6, 11.5)	61.7 (38.2, 88.7)	63361 (17503, 129140)	3.1 (0.9, 6.2)	114.9 (32.2, 232.2)	67.4 (44.4, 96.6)

DALY, Disability adjusted life year; GBD, Global Burden of Disease; ASRs, Age-standardized rates.

### Regional Level

In 2019, the total number of cancer-related deaths attributable to HFPG were highest in East Asia (94,519 [24,177 to 204,701]), Western Europe (74,827 [21,400 to 148,875]) and High-income North America (62,291 [17,929 to 122,539]). In contrast, the lowest numbers were found in Oceania (486 [131 to 1,052]), Central Sub-Saharan Africa (1492 [370 to 3,423]) and Andean Latin America (1,694 [462 to 3,598]) (**Table 1**). The proportion of all cancer-related deaths (PAFs) that were attributable to HFPG ranged from 1.4% to 7.2%. High-income North America (7.2% [2.1 to 14.1]), Central Europe (6.0% [1.7 to 12.0]) and Western Europe (5.8% [1.7 to 11.5]) had the three highest PAFs, while the lowest were found in Eastern Sub-Saharan Africa (1.4% [0.4 to 2.9]), Western Sub-Saharan Africa (2.1% [0.6 to 4.4]) and Andean Latin America (2.6% [0.7 to 5.3]) (**Table 1**).

The age-standardized death rates for cancers attributable to HFPG (per 100,000) in 2019 were highest in High-income North America (9.5 [2.7 to 18.8]), Central Europe (9.3 [2.6 to 19.2]) and Western Europe (7.5 [2.1 to 15.1]). The lowest rates were observed in Eastern Sub-Saharan Africa (2.0 [0.5 to 4.2]), Western Sub-Saharan Africa (2.7 [0.7 to 5.7]) and South Asia (3.0 [0.8 to 2.6]) (**Table 1**). Most GBD regions saw increases in the age-standardized death rates of cancers attributable to HFPG from 1990-2019, with the largest increases being observed in South Asia (95.6% [66.1 to 137.3]), North Africa and the Middle East (93.9% [68.3 to 133.3]) and Western Sub-Saharan Africa (88.1% [60.1 to 122.8]) (**Table 1**). The age-standardized death rates of cancers attributable to HFPG (per 100,000) in 2019 are presented in **Figure S1**, by region and sex, and the changes from 1990 to 2019 are presented in **Figure S2**, by region and sex.

In 2019, the total number of cancer-related DALYs attributable to HFPG were highest in East Asia (2,034,530 [517,721 to 4,451,841]), Western Europe (1,293,209 [369,006 to 2,586,197]) and High-income North America (1,203,538 [343,531 to 2,366,869). The lowest number of DALYs were in Oceania (13,829 [3,717 to 30,700]), Andean Latin America (32,792 [8,962 to 70,558]) and Central Sub-Saharan Africa (37,203 [8,966 to 86,838]) (**Table 1**). The proportion of all cancer-related DALYs that were attributable to HFPG ranged from 0.9% to 6.5%. High-income North America (6.5% [1.9 to 12.8]), Central Europe (5.4% [1.5 to 10.9]) and Western Europe (5.2% [1.4 to 10.4]) had the three highest PAFs, while the lowest were found in Western Sub-Saharan Africa (0.9% [0.3 to 2.0]), Andean Latin America (1.5% [0.4 to 3.1]) and Eastern Sub-Saharan Africa (2.0% [0.5 to 4.1]) (**Table 1**).

In 2019, the age-standardized DALY rates of cancers attributable to HFPG (per 100,000) were highest in Central Europe (196.5 [53.6 to 413.1]), High-income North America (191.4 [54.4 to 376.8]) and Oceania (177.4 [48.0 to 384.6]). Conversely, the lowest rates were found in Eastern Sub-Saharan Africa (39.7 [10.4 to 84.8]), Western Sub-Saharan Africa (52.5 [14.2 to 112.3]) and Andean Latin America (59.5 [16.2 to 127.7]) (**Table 1**). Most GBD regions showed increases in the age-standardized DALY rates of cancer attributable to HFPG from 1990 to 2019, with the largest increases being seen in South Asia (100.5% [69.7 to 142.2]), Western Sub-Saharan Africa (90.2% [60.0 to 125.5]) and North Africa and the Middle East (90.1% [64.4 to 127.8]) (**Table 1**). The age-standardized DALY rates of cancers attributable to HFPG (per 100,000) in 2019 are presented in **Figure S3**, by region and sex, and the changes from 1990 to 2019 are presented in **Figure S4**, by region and sex.

Globally, the number of deaths attributable to HFPG increased from 150,100 (UI 39,209 to 312,371) in 1990 to 419,338 (UI 115,729 to 848,484) in 2019. In 2019, East Asia (94,519 [24,177 to 204,701]), Western Europe (74,827 [21,400 to 148,875]) and High-income North America (62,291 [17,929 to 122,539]) had the highest number of cancer related deaths attributable to high fasting plasma glucose. Similarly, in 1990 the number of deaths were highest in Western Europe (37,979 [10,073 to 77,067]), High-income North America (29,867 [7,965 to 60,987]) and East Asia (25,734 [6,560 to 55,611]) (**Figure S5** and **Table S2**). This pattern was also seen in the number of cancer DALYs attributable to HFPG in 1990 and 2019 (**Figure S6** and **Table S4**).

**Figure 1:**
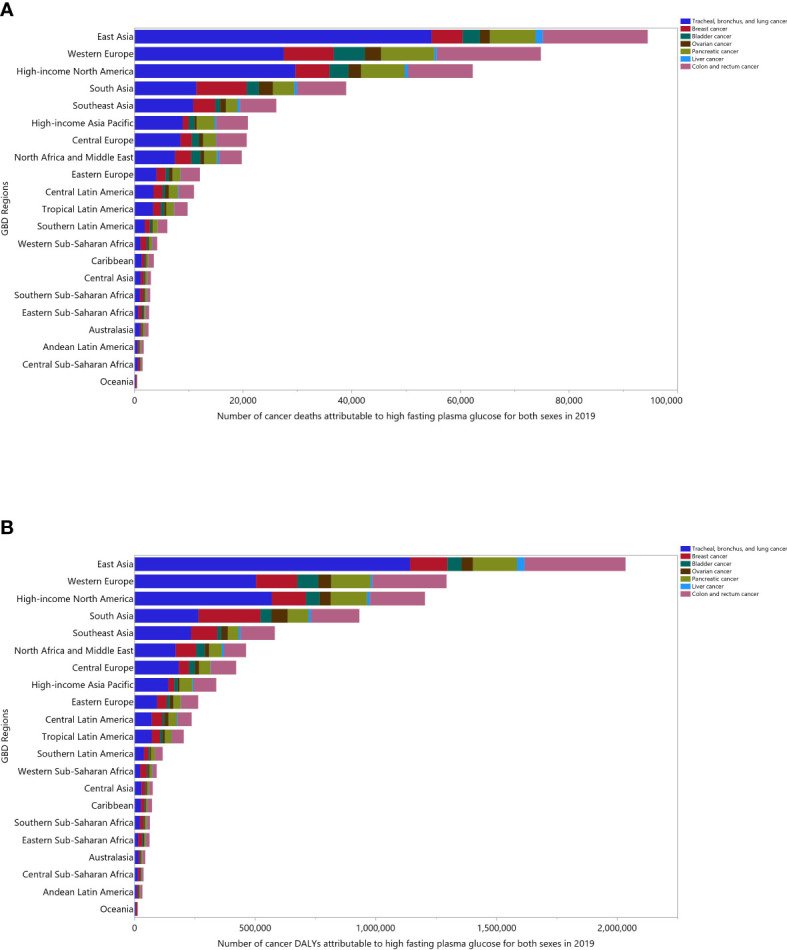
Number of cancer deaths **(A)** and DALYs **(B)** attributable to high fasting plasma glucose in 2019 by cancer types and GBD region. DALY= disability-adjusted-life-years (Generated from data available from http://ghdx.healthdata.org/gbd-results-tool).

The contribution of each cancer type attributable to HFPG also varied by region. The number of deaths due to tracheal, bronchus and lung (TBL) cancers were highest in all regions, except Eastern Sub-Saharan Africa. Colon and rectal cancer had the second highest contribution in most GBD regions, while in some regions TBL cancer (Eastern Sub-Saharan Africa) and breast cancer (Central Sub-Saharan Africa, Oceania, South Asia and Southern Sub-Saharan Africa) were found to be the second largest contributors to the number of deaths attributable to HFPG (**Figure 1A**). The contribution of each cancer to the number of all-cancer related DALYs attributable to HFPG, also differed by region (**Figure 1B**).

### National Level

**Figure 2:**
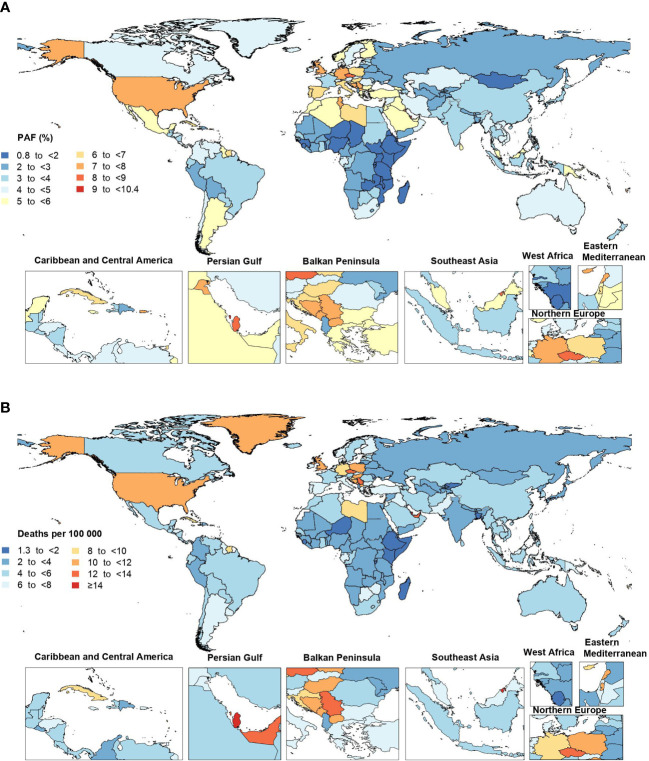
Population attributable fraction (PAF) **(A)** and age-standardized rates **(B)** of cancer deaths attributable to high fasting plasma glucose in 2019, by country. (Generated from data available from http://ghdx.healthdata.org/gbd-results-tool).

In 2019, the proportion of all cancer-related deaths that were attributable to HFPG differed substantially by country (from 0.9% to 10.3%). Bahrain (10.3% [3.3 to 18.9]), American Samoa (10.1% [3.2 to 18.6]) and Niue (9.9% [3.0 to 19.0]) had the three highest PAFs. In contrast, the lowest PAFs were found in Mongolia (0.9% [0.2 to 1.9]), Somalia (1.0% [0.2 to 2.2]) and Burundi (1.2% [0.3 to 2.5]) (**Figure 2A** and **Table S2**).

The age-standardized death rate of cancers attributable to HFPG in 2019 ranged from 1.4 to 19.9 per 100,000. Brunei Darussalam (19.9 [6.2 to 37.4]), Qatar (15.8 [5.1 to 30.3]) and American Samoa (15.5 [4.9 to 29.9]) had the three highest age-standardized death rates for cancers attributable to HFPG (per 100,000) in 2019. In contrast, the lowest rates were found in Ethiopia (1.4 [0.4 to 3.2]), Niger (1.4 [0.4 to 3.3]) and Somalia (1.5 [0.3 to 3.8]) (**Figure 2B** and **Table S2**). Increases in the age-standardized death rates for cancers attributable to HFPG were found in several countries and territories over the measurement period. Cabo Verde (310.7% [219.2 to 426.1]), Egypt (204.4% [122.5 to 323.9]) and Uzbekistan (190.1% [139.7 to 269.6]) saw the largest increases in the age-standardized death rates from 1990 to 2019. In contrast, Singapore (-37.4% [-44.1 to -30.6]) was the only country that showed a decrease over this period (**Table S2**).

The proportion of all cancer-related DALYs that were attributable to HFPG in 2019 also differed considerably by country (from 0.7% to 9.2%). American Samoa (9.2% [2.9 to 17.2]), Niue (9.1% [2.7 to 17.5]) and Bahrain (8.2% [2.6 to 15.3]) had the three highest PAFs. In contrast, the lowest PAFs were found in Somalia (0.7% [0.2 to 1.5]), Mongolia (0.7% [0.2 to 1.6]) and Ethiopia (0.8% [0.2 to 1.7]) (**Figure S7** and **Table S4**). The age-standardized DALY rate of cancers attributable to HFPG in 2019 ranged from 27.4 to 379.0 per 100,000. Brunei Darussalam (379.0 [117.5 to 725.8]), the Marshall Islands (344.3 [95.3 to 772.9]) and American Samoa (342.3 [106.6 to 674.3]) had the three highest age-standardized DALY rates. In contrast, the lowest rates were observed in Ethiopia (27.4 [6.7 to 61.9]), Niger (27.6 [6.8 to 63.1]) and Somalia (31.4 [7.2 to 80.7]) (**Figure S8** and **Table S4**). Cabo Verde (284.7% [195.9 to 396.5]), the Solomon Islands (212.7% [126.5 to 363.8]), and Egypt (209.3% [125.9 to 331.7]) showed the largest increases in the age-standardized DALY rates of cancers attributable to HFPG from 1990 to 2019. In contrast, Singapore (-42.7% [-48.5 to -36.4]) was again the only country to show a decrease over the measurement period (**Table S4**).

### Age and Sex Patterns

**Figure 3:**
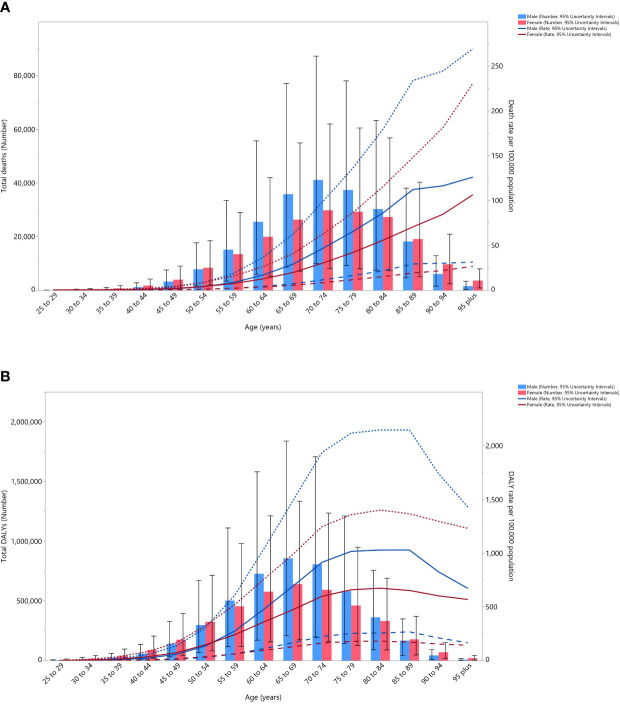
Global number of deaths and death rate **(A)** and global number of DALYs and DALY rate **(B)** of cancers attributable to high fasting plasma glucose (per 100,000) by age and sex in 2019; Dotted and dashed lines indicate 95% upper and lower uncertainty intervals, respectively. DALY= disability-adjusted-life-years. (Generated from data available from http://ghdx.healthdata.org/gbd-results-tool).

In 2019, the global number of cancer deaths attributable to HFPG was highest in the 70-74 age group. The death rate of cancers that was attributable to HFPG started to increase from the 45-49 age group and peaked in the oldest (95^+^) age group for both males and females. There were no substantial differences between males and females, in terms of the number of deaths and the death rate (**Figure 3A**). Moreover, in 2019 the global number of DALYs was highest in the 65-69 age group. The DALY rate started increasing in the early age groups, peaking in the 80-84 age group and then decreasing for both males and females (**Figure 3B**).

### Burden of Cancers Attributable to High Fasting Plasma Glucose by Socio-Demographic Index

**Figure 4:**
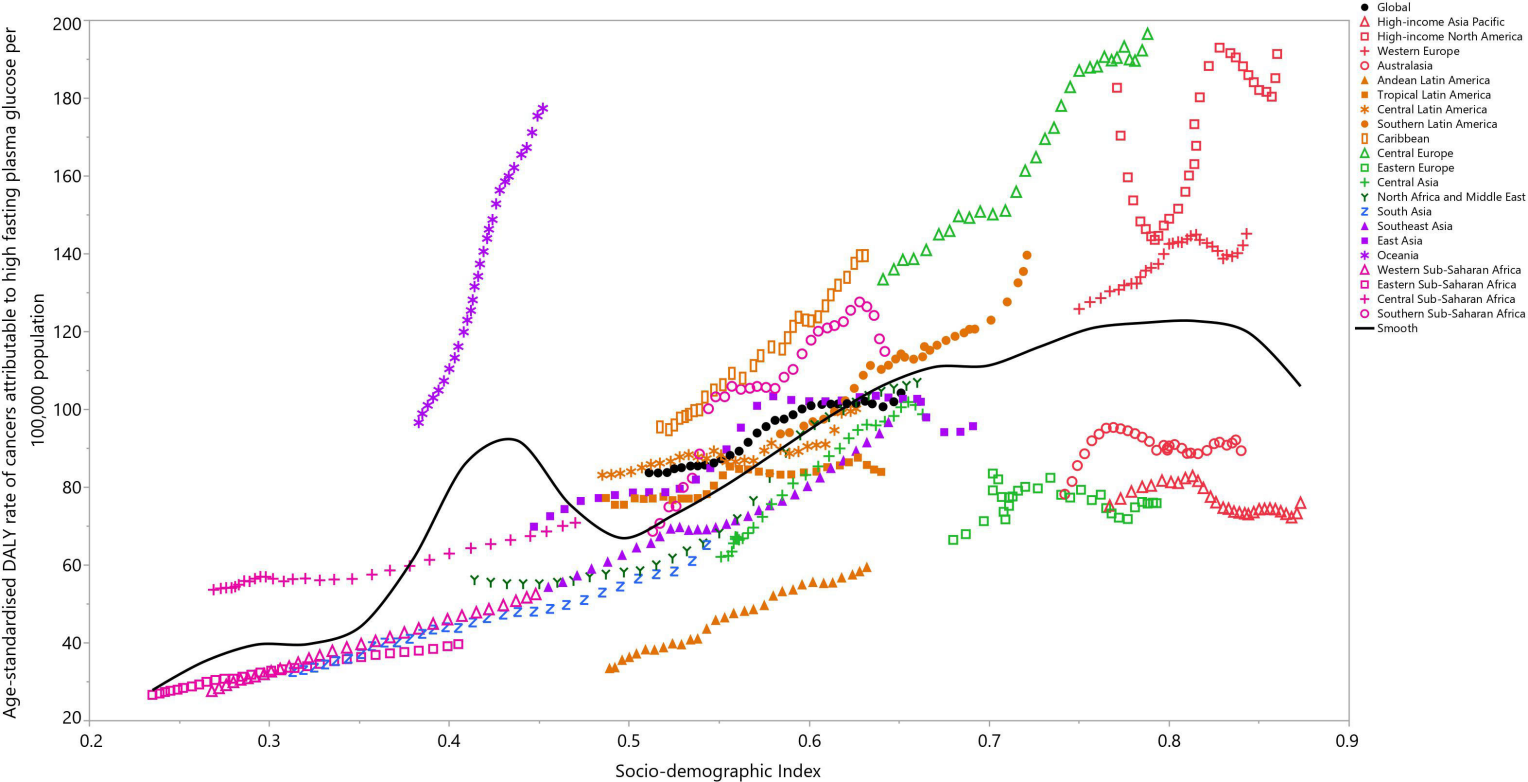
Age-standardized DALY rates of cancers attributable to high fasting plasma glucose for the 21 Global Burden of Disease regions by Socio-demographic Index, 1990–2019; Expected values based on Socio-demographic Index and disease rates in all locations are shown as the black line. Thirty points are plotted for each GBD region and show the observed age-standardized DALY rates from 1990 to 2019 for that region. DALY= disability-adjusted-life-years. (Generated from data available from http://ghdx.healthdata.org/gbd-results-tool).

There was generally a positive association between the regional SDI and the corresponding age-standardized DALY rates of all cancers attributable to HFPG, from 1990 to 2019. Regions higher than the solid black line had a higher than expected burden (based on SDI), while those below the line had a lower than expected burden. Most of the GBD regions saw an increase in the age-standardized DALY rate across the measurement period. High-income North America, Western Europe, Central Europe, Caribbean, Southern Sub-Saharan Africa and Oceania had higher than expected burdens from 1990 to 2019. In contrast, the burden of cancers attributable to HFPG was lower than expected for High-income Asia-Pacific, Australasia, Eastern Europe, Central Asia, Andean Latin-America, South Asia, Western Sub-Saharan Africa and Eastern Sub-Saharan Africa during the measurement period (**Figure 4**).

The association between the age-standardized DALY rate of cancers attributable to HFPG in 2019 and each country’s SDI was also generally positive (**Figure S9**). Countries and territories, such as Brunei Darussalam, American Samoa, the Marshall Islands, Palau, Niue and Nauru had much higher than expected levels of burden. In contrast, the corresponding burdens were much lowest than expected for several countries, including Japan, Belarus, Turkmenistan, Kyrgyzstan, Kenya, and Madagascar (**Figure S9**).

## Discussion

The present study found that the global HFPG-attributable age-standardized death and DALY rate of cancers increased by 27.8% and 24.5%, respectively, from 1990 to 2019 and that HFPG accounted for a large number of cancer related deaths and DALYs in 2019. In addition, the HFPG-attributable burden of cancers had an approximately positive association with SDI and advancing age.

In terms of NCDs, in 2019 15.2% of all-age deaths and 10.4% of all-age DALYs were attributable to HFPG (1). Using GBD 2019 data, we found that 4.2% of deaths and 3.4% of all cancer-related DALYs were attributable to HFPG. In fact, about one third of the HFPG-attributable burden of NCDs were due to cancers. Furthermore, the same 2017 study found that among cancers, in decreasing order, tracheal, bronchus and lung cancers, colon and rectal cancers, breast cancer and pancreatic cancer had the highest DALYs attributable to HFPG (11), which concurs with our findings. A systematic review and meta-analysis also showed a 70% higher risk of cancer incidence in diabetic patients than among non-diabetics (21) and another study found that diabetes mellitus was associated with poor prognosis and higher all-cause mortality among patients with cancers (22). Since diabetes is associated with a poor prognosis and higher incidence of cancers, facilitation of access and reducing the costs of screening programs like mammography or colonoscopy could lead to earlier detection and treatment of cancers in these populations (23).

In 2019, Oceania (3703.4), Southern Sub-Saharan Africa (1878.0), and Central Latin America (1746.5) had the highest burdens of diabetes, in terms of the age-standardized rate of DALYs per 100 000 population (24). In our 2019 estimates, Central Europe, High-income North America and Oceania had the three highest age-standardized burdens of cancers attributable to HFPG. This data revealed that it is not only a higher burden of diabetes in a region that affects the HFPG-attributable burden of cancers, as shown in Oceania, health care programs and interventions can lead to a reduction in the attributable burden of cancers attributable to HFPG.

The cancer death rates attributable to HFPG increased with age. In line with our findings, a 2017 study, which investigated the global burden of NCDs attributable to HFPG, found an increasing crude mortality rate with advancing age (8). Moreover, in 2019 the global age-standardized point prevalence, death, and DALYs of diabetes increased slightly with advancing age and peaked among the elderly (24). The higher deaths and DALYs among older people could be as a result of the higher prevalence of diabetes in these ages, comorbidities and immunosenescence, which can increase both morbidity and mortality (25).

We observed a positive association between the age-standardized DALY rate of cancers attributable to HFPG and the SDI. The study by Khan et al. showed a higher prevalence of diabetes in developed regions, and that the DALY rates were increasing (25). Furthermore, Lin and colleagues reported that the age-standardized DALY rate of type 2 diabetes mellitus was increasing in all World Bank regions and that the World Bank lower middle income region showed the greatest increases between 1990 and 2017 (5). Moreover, an article reporting the burden of diabetes, using GBD 2019 data, showed no clear association between the age-standardized DALY rate and SDI at the regional level, but at the national level an increase was seen to around the middle SDI quintile, followed by a decrease (24). In contrast, a negative association was found between the global burden of NCDs attributable to HFPG and SDI (8), although this discrepancy with our findings might be as a result of variations in the methodologies and including all NCDs, instead of focusing only on cancers.

The current study found that the burden of cancers attributable to HFPG has increased over the last 30 years, although the PAF has not changed substantially. Regions like High-income North America or Central Europe and countries like Brunei Darussalam or American Samoa had substantial attributable burdens in 2019. Furthermore, overall we found positive associations with age and socioeconomic development. The findings of the present study can be used by regional and national health authorities and health policy-makers to inform efforts to reduce those cancers attributable to HFPG. Nevertheless, we provide some general evidence-based recommendations here. To reduce the burden of cancers attributable to HFPG, health policy makers can approach this in two ways, which are modifications in the risk factors of diabetes to reduce its burden or the early diagnosis and treatment of cancers to reduce their complications and mortalities. High body mass index, unhealthy diet (e.g. diets low in whole grains, fruits, nuts and seeds, and high in sugar-sweetened beverages, processed meat and red meat) first- or second-hand smoking, low physical activity, air pollution, stress and hypertension are all risk factors for the development of diabetes (5, 26). Interventions for the prevention of diabetes include lifestyle modifications, such as increasing physical activity to at least 150 minutes per week and weight loss, in addition to pharmacologic interventions like metformin (27). These recommendations are not new and efforts to implement them are well studied in the scientific literature (28–32). However, they are not routinely implemented and even when they are, sustained behavior change remains elusive. Therefore, policy efforts need to focus on implementation sustainability in intervention outcomes and mechanisms to increase behavior change, rather than efficacy of individual intervention approaches, which has already been well resourced and studied. In particular, a strong evidence base already supports diabetes prevention in response to even small weight reductions. Therefore, policy efforts could begin by focusing on small changes to behavior that may reap substantial reward for investment. Despite the implementation of the above mentioned interventions, if cancers occurred, the development of secondary preventive programs, like using mammography for breast cancer, fecal occult blood tests, sigmoidoscopy or colonoscopy for colorectal cancer can reduce the morbidity and mortality of cancers in diabetic patients (33, 34).

### Strengths and Limitations of This Study

This is the first study to report the burden of cancers attributable to HFPG, using the most recent data from the GBD project. This study also reported the burden associated with demographic features, such as age, sex, cancer type and sociodemographic factors. However, we acknowledge that the current article has some limitations. Firstly, the subtypes of diabetes were not differentiated in our study and due to the higher prevalence of type 2 diabetes, particular among adults, these results represent the burden of cancers mostly attributable to type 2 diabetes. Secondly, the attributable burden of cancer by tumor location or the molecular or pathological subtypes of cancers were not reported. For instance, the data were not differentiated between tracheal, bronchus and lung cancers and the location of colon cancer (i.e. ascending, transverse and descending colon) was not estimated. Thirdly, our results are limited to individuals aged above 25 years old, as children and adolescents were not included. Fourthly, the burden of cancers attributable to HFPG were estimated at the global, regional, and national levels, however, the attributable burden in sub-national levels were not included. As there are potential sub-national variations in the prevalence of HFPG within a country, further studies on sub-national levels could be helpful for policy makers. Finally, there was poor quality data on the burden of cancer or HFPG in some countries, which might affect the validity of the estimates provided by our model and made wide UIs for some of the estimates. It is noteworthy that most of these limitations were due to the GBD methodology and were thus beyond the control of the authors (11).

## Conclusions

Although less than 5% of cancer deaths and DALYs were attributable to HFPG, there was a more than 20% increase in the age-standardized death and DALY rate of cancers that were attributable to HFPG between 1990 and 2019. The attributable burden increased with an increasing level of development, as measured by the SDI. Moreover, cancers of the respiratory and gastrointestinal systems were highly attributable to HFPG. Therefore, health authorities and policy makers need to urgently increase efforts to stem the growth of cancer burden caused by HFPG, by considering increases to resource allocation, design and implementation of diabetes prevention programs and prioritization of cancer screening efforts, particularly for lung and colorectal cancers among diabetic patients. Further studies should also focus on the effects of HFPG on each subtype of cancer, the attributable burden in children and adolescents, and evaluate the effects of healthcare measures on the burden of cancers attributable to HFPG.

## Author Note

This study is based on publicly available data and solely reflects the opinion of its authors and not that of the Institute for Health Metrics and Evaluation.

## Data Availability Statement

Publicly available datasets were analyzed in this study. This data can be found here: http://ghdx.healthdata.org/gbd-results-tool.

## Ethics Statement

The present study was approved by ethical committee of Shahid Beheshti University of Medical Sciences, Tehran, Iran (IR.SBMU.RETECH.REC.1400.431).

## Author Contributions

SS, NK and A-AK designed the study. SS, NLB, AA-H and MAM analyzed the data and performed the statistical analyses. SS, SAN, NK, JSK, KCC, MJMS, MRB, GSC and A-AK drafted the initial manuscript. All authors reviewed the drafted manuscript for critical content. All authors approved the final version of the manuscript.

## Funding

The Bill and Melinda Gates Foundation, who were not involved in any way in the preparation of this manuscript, funded the GBD study. The Shahid Beheshti University of Medical Sciences also supported the present report (Grant No. 29055).

## Conflict of Interest

The authors declare that the research was conducted in the absence of any commercial or financial relationships that could be construed as a potential conflict of interest.

## Publisher’s Note

All claims expressed in this article are solely those of the authors and do not necessarily represent those of their affiliated organizations, or those of the publisher, the editors and the reviewers. Any product that may be evaluated in this article, or claim that may be made by its manufacturer, is not guaranteed or endorsed by the publisher.

## Supplementary Material

Supplementary Table 1 Number, proportion and age-standardized rates of cancer deaths attributable to high fasting plasma glucose (per 100,000) in 2019, by sex and location (Generated from data available from http://ghdx.healthdata.org/gbd-results-tool)

Supplementary Table 2 Table S2: Number, proportion and age-standardized rates of cancer deaths attributable to high fasting plasma glucose (per 100,000) in 1990 and 2019, by location (Generated from data available from http://ghdx.healthdata.org/gbd-results-tool)

Supplementary Table 3 Number, proportion and age-standardized rates of disability-adjusted-life-years (DALYs) due to cancers attributable to high fasting plasma glucose (per 100,000) in 2019, by sex and location (Generated from data available from http://ghdx.healthdata.org/gbd-results-tool)

Supplementary Table 4 Number, proportion and age-standardized rates of disability-adjusted-life-years (DALYs) due to cancers attributable to high fasting plasma glucose (per 100,000) in 1990 and 2019, by location (Generated from data available from http://ghdx.healthdata.org/gbd-results-tool)

Supplementary Figure 1 The age-standardized death rate of cancers attributable to high fasting plasma glucose in 2019 for the 21 Global Burden of Disease regions, by sex. (Generated from data available from http://ghdx.healthdata.org/gbd-results-tool).

Supplementary Figure 2 The percentage change in the age-standardized death rate of cancers attributable to high fasting plasma glucose from 1990 to 2019 for the 21 Global Burden of Disease regions, by sex. (Generated from data available from http://ghdx.healthdata.org/gbd-results-tool).

Supplementary Figure 3 The age-standardized DALY rate of cancers attributable to high fasting plasma glucose in 2019 for the 21 Global Burden of Disease regions, by sex. DALY= disability-adjusted-life-years. (Generated from data available from http://ghdx.healthdata.org/gbd-results-tool).

Supplementary Figure 4 The percentage change in the age-standardized DALY rate of cancers attributable to high fasting plasma glucose from 1990 to 2019 for the 21 Global Burden of Disease regions, by sex. DALY=disability-adjusted-life-years. (Generated from data available from http://ghdx.healthdata.org/gbd-results-tool).

Supplementary Figure 5 Number of cancer deaths attributable to high fasting plasma glucose from 1990 to 2019 for the 21 Global Burden of Disease regions. (Generated from data available from http://ghdx.healthdata.org/gbd-results-tool).

Supplementary Figure 6 Number of cancer DALYs attributable to high fasting plasma glucose from 1990 to 2019 for the 21 Global Burden of Disease regions. DALY=disability-adjusted-life-years. (Generated from data available from http://ghdx.healthdata.org/gbd-results-tool).

Supplementary Figure 7 Population attributable fraction (PAF) of DALYs due to cancers attributable to high fasting plasma glucose in 2019, by country. DALY=disability-adjusted-life-years. (Generated from data available from http://ghdx.healthdata.org/gbd-results-tool).

Supplementary Figure 8 Age-standardized rate of DALYs due to cancers that were attributable to high fasting plasma glucose in 2019, by country. (Generated from data available from http://ghdx.healthdata.org/gbd-results-tool).

Supplementary Figure 9 Age-standardized DALY rates of cancers attributable to high fasting plasma glucose by Socio-demographic Index for 204 countries and territories in 2019; Expected values are shown as the black line. Each point shows observed age-standardized DALY rate for specified country in 2019. DALY=disability-adjusted-life-years. (Generated from data available from http://ghdx.healthdata.org/gbd-results-tool).

## Abbreviations

HFPG, high fasting plasma glucose; DALY, Disability-adjusted life years; NCD, Non-communicable disease; SDI, Socio-demographic Index; GBD, Global Burden of Disease; PAF, population-attributable fraction; TMREL, theoretical minimum risk exposure level; UI, uncertainty intervals; ICD, International Classification of Disease.
