# Supplementary material for: Global, Regional and National Burden of Cancers Attributable to High Fasting Plasma Glucose in 204 Countries and Territories, 1990-2019
Source: Front Endocrinol (Lausanne). 2022 Jul 19;13:879890. doi: 10.3389/fendo.2022.879890 (PMC9366927; doi:10.3389/fendo.2022.879890)
Supplement: Supplementary Table 1 — Number, proportion and age-standardized rates of cancer deaths attributable to high fasting plasma glucose (per 100,000) in 2019, by sex and location (Generated from data available from http://ghdx.healthdata.org/gbd-results-tool) [file Table_1.doc]

| **Table S1: Number, proportion and age-standardised rates of cancer deaths attributable to high fasting plasma glucose (per 100,000) in 2019, by sex and location**  **(Generated from data available from http://ghdx.healthdata.org/gbd-results-tool)** | | | | | | |
| --- | --- | --- | --- | --- | --- | --- |
|  | **Male** | | | **Female** | | |
|  | **No**  **(95% UI)** | **PAF**  **(95% UI)** | **ASRs per 100,000 (95% UI)** | **No**  **(95% UI)** | **PAF**  **(95% UI)** | **ASRs per 100,000 (95% UI)** |
| **Global** | **224830 (55522 , 481518)** | **3.9 (1 , 8.3)** | **6.3 (1.6 , 13.4)** | **194508 (53409 , 409512)** | **4.5 (1.2 , 9.1)** | **4.4 (1.2 , 9.3)** |
| **High-income North America** | **32337 (8402 , 65679)** | **7 (1.8 , 14.1)** | **11.1 (2.9 , 22.5)** | **29955 (8330 , 61136)** | **7.4 (2 , 15)** | **8.3 (2.3 , 16.8)** |
| **Canada** | **2135 (505 , 4664)** | **4.3 (1 , 9.3)** | **6.5 (1.5 , 14.2)** | **2014 (515 , 4252)** | **4.7 (1.2 , 9.8)** | **5.1 (1.3 , 10.7)** |
| **Greenland** | **5 (1 , 10)** | **5.1 (1.2 , 10.9)** | **14.1 (3.3 , 31.5)** | **2 (1 , 6)** | **3.9 (1 , 8.4)** | **8.2 (2 , 18.6)** |
| **United States of America** | **30197 (7922 , 61175)** | **7.3 (1.9 , 14.7)** | **11.7 (3.1 , 23.6)** | **27938 (7797 , 56879)** | **7.8 (2.1 , 15.6)** | **8.6 (2.4 , 17.6)** |
| **Australasia** | **1333 (324 , 2827)** | **3.7 (0.9 , 7.8)** | **5.5 (1.3 , 11.7)** | **1240 (323 , 2615)** | **4.4 (1.1 , 9.2)** | **4.3 (1.1 , 9)** |
| **Australia** | **1141 (278 , 2420)** | **3.8 (0.9 , 8)** | **5.6 (1.4 , 11.9)** | **1032 (268 , 2186)** | **4.4 (1.2 , 9.2)** | **4.2 (1.1 , 8.9)** |
| **New Zealand** | **191 (47 , 407)** | **3.4 (0.8 , 7.1)** | **5.1 (1.2 , 10.8)** | **208 (54 , 444)** | **4.3 (1.1 , 9.1)** | **4.6 (1.2 , 9.9)** |
| **High-income Asia Pacific** | **12957 (3113 , 28019)** | **4 (1 , 8.5)** | **6 (1.4 , 13)** | **7912 (2045 , 17154)** | **3.4 (0.9 , 7.3)** | **2.6 (0.7 , 5.5)** |
| **Brunei Darussalam** | **23 (6 , 46)** | **8.9 (2.5 , 16.9)** | **27.2 (7.7 , 52.8)** | **22 (6 , 43)** | **8.1 (2.3 , 15.8)** | **16.6 (4.9 , 32.8)** |
| **Japan** | **9821 (2316 , 21130)** | **3.8 (0.9 , 8.2)** | **5.6 (1.3 , 12)** | **5973 (1507 , 13085)** | **3.2 (0.8 , 6.8)** | **2.3 (0.6 , 5.1)** |
| **Singapore** | **205 (49 , 435)** | **5.3 (1.3 , 11.2)** | **6.1 (1.5 , 12.9)** | **158 (42 , 334)** | **5 (1.3 , 10.5)** | **3.9 (1 , 8.3)** |
| **Republic of Korea** | **2908 (696 , 6217)** | **4.6 (1.1 , 9.8)** | **8 (1.9 , 17.1)** | **1759 (466 , 3713)** | **4.3 (1.2 , 9)** | **3.4 (0.9 , 7.2)** |
| **Western Europe** | **38948 (9996 , 80978)** | **5.4 (1.4 , 11.1)** | **9.1 (2.3 , 19)** | **35879 (9913 , 73381)** | **6.3 (1.7 , 12.6)** | **6.3 (1.7 , 12.8)** |
| **Andorra** | **7 (2 , 16)** | **4.6 (1.1 , 9.6)** | **10.2 (2.4 , 22.6)** | **3 (1 , 7)** | **4.1 (1.1 , 8.5)** | **4.1 (1 , 9.3)** |
| **Austria** | **512 (129 , 1090)** | **4.2 (1.1 , 9)** | **6.3 (1.6 , 13.5)** | **510 (135 , 1069)** | **5 (1.3 , 10.4)** | **4.6 (1.2 , 9.7)** |
| **Belgium** | **925 (222 , 1984)** | **4.9 (1.2 , 10.3)** | **8.7 (2.1 , 18.7)** | **815 (214 , 1710)** | **5.5 (1.5 , 11.4)** | **5.7 (1.5 , 11.9)** |
| **Cyprus** | **93 (23 , 191)** | **7.1 (1.8 , 14.5)** | **10 (2.5 , 20.8)** | **70 (19 , 142)** | **7.1 (2 , 14)** | **6.7 (1.9 , 13.7)** |
| **Denmark** | **393 (98 , 833)** | **4 (1 , 8.5)** | **7 (1.7 , 14.8)** | **459 (120 , 973)** | **5.3 (1.4 , 11.2)** | **6.8 (1.8 , 14.4)** |
| **Finland** | **401 (103 , 834)** | **5.2 (1.3 , 10.9)** | **7 (1.8 , 14.6)** | **461 (128 , 949)** | **6.8 (1.9 , 13.6)** | **6 (1.7 , 12.4)** |
| **France** | **3433 (795 , 7592)** | **3 (0.7 , 6.6)** | **5.5 (1.3 , 12.3)** | **2891 (734 , 6292)** | **3.5 (0.9 , 7.3)** | **3.2 (0.8 , 7)** |
| **Germany** | **9527 (2476 , 19676)** | **6.4 (1.7 , 13)** | **10.6 (2.8 , 22.1)** | **9889 (2699 , 19927)** | **8 (2.2 , 15.9)** | **8.3 (2.3 , 16.8)** |
| **Greece** | **1171 (281 , 2519)** | **5.6 (1.3 , 12.1)** | **10.3 (2.4 , 22.2)** | **730 (193 , 1515)** | **5.1 (1.3 , 10.6)** | **4.9 (1.3 , 10.2)** |
| **Iceland** | **15 (4 , 33)** | **4.1 (1 , 8.6)** | **5.8 (1.4 , 12.4)** | **14 (4 , 30)** | **5.1 (1.3 , 10.7)** | **4.3 (1.1 , 9.4)** |
| **Ireland** | **265 (65 , 553)** | **4.7 (1.2 , 9.9)** | **7.5 (1.8 , 15.6)** | **249 (66 , 528)** | **5.2 (1.4 , 10.8)** | **5.9 (1.6 , 12.6)** |
| **Israel** | **367 (93 , 775)** | **4.8 (1.2 , 10.1)** | **6.9 (1.7 , 14.5)** | **417 (114 , 858)** | **6 (1.6 , 12.2)** | **6.1 (1.6 , 12.6)** |
| **Italy** | **6633 (1688 , 13590)** | **6.2 (1.6 , 12.5)** | **9.9 (2.5 , 20.2)** | **5233 (1441 , 10717)** | **6.2 (1.7 , 12.5)** | **5.7 (1.6 , 11.6)** |
| **Luxembourg** | **51 (13 , 106)** | **7.1 (1.9 , 14.5)** | **11.1 (2.9 , 23.1)** | **44 (12 , 92)** | **7.5 (2.1 , 15.2)** | **7.4 (2 , 15.7)** |
| **Malta** | **37 (10 , 78)** | **6.5 (1.6 , 13.3)** | **8.4 (2.2 , 17.6)** | **33 (9 , 69)** | **7.8 (2.2 , 15.5)** | **6.1 (1.7 , 12.7)** |
| **Monaco** | **6 (1 , 14)** | **5 (1.2 , 10.9)** | **13.4 (3.1 , 29.2)** | **6 (1 , 12)** | **5.5 (1.5 , 11.5)** | **9.7 (2.5 , 21.5)** |
| **Netherlands** | **1286 (311 , 2828)** | **4.2 (1 , 9.1)** | **7.9 (1.9 , 17.3)** | **1158 (307 , 2474)** | **4.5 (1.2 , 9.5)** | **5.8 (1.5 , 12.3)** |
| **Norway** | **326 (84 , 674)** | **4.8 (1.2 , 9.9)** | **7.1 (1.8 , 14.7)** | **373 (101 , 779)** | **6.4 (1.7 , 13)** | **6.6 (1.8 , 13.7)** |
| **Portugal** | **1074 (286 , 2221)** | **5.7 (1.5 , 11.8)** | **9.9 (2.6 , 20.6)** | **893 (245 , 1839)** | **6.9 (1.9 , 13.9)** | **5.7 (1.6 , 11.8)** |
| **San Marino** | **3 (1 , 8)** | **4.6 (1.2 , 9.7)** | **10.1 (2.5 , 24.4)** | **2 (1 , 6)** | **4.8 (1.3 , 9.9)** | **5.7 (1.4 , 15.4)** |
| **Spain** | **4861 (1254 , 10207)** | **6.4 (1.7 , 13.4)** | **11.1 (2.8 , 23.4)** | **3091 (851 , 6386)** | **6.1 (1.7 , 12.5)** | **5 (1.4 , 10.3)** |
| **Sweden** | **571 (148 , 1191)** | **4.1 (1.1 , 8.6)** | **5.4 (1.4 , 11.2)** | **674 (179 , 1403)** | **5.4 (1.4 , 11.1)** | **5.4 (1.4 , 11.2)** |
| **Switzerland** | **487 (120 , 1038)** | **4.3 (1.1 , 9.2)** | **5.9 (1.5 , 12.6)** | **495 (134 , 1041)** | **5.5 (1.5 , 11.3)** | **4.7 (1.3 , 9.9)** |
| **United Kingdom** | **6469 (1695 , 13104)** | **6.2 (1.6 , 12.5)** | **10.7 (2.8 , 21.7)** | **7337 (2065 , 14824)** | **8 (2.2 , 16.1)** | **9.7 (2.7 , 19.7)** |
| **Southern Latin America** | **2900 (726 , 6095)** | **4.3 (1.1 , 9.2)** | **8 (2 , 16.8)** | **3134 (827 , 6481)** | **5.2 (1.4 , 10.6)** | **6.3 (1.7 , 13.1)** |
| **Argentina** | **2071 (517 , 4359)** | **4.6 (1.2 , 9.7)** | **8.9 (2.2 , 18.8)** | **2150 (557 , 4476)** | **5.3 (1.4 , 10.9)** | **6.7 (1.7 , 14)** |
| **Chile** | **612 (156 , 1300)** | **3.7 (0.9 , 7.7)** | **5.8 (1.5 , 12.3)** | **800 (222 , 1651)** | **5.3 (1.5 , 10.8)** | **5.8 (1.6 , 12.1)** |
| **Uruguay** | **217 (54 , 466)** | **4 (1 , 8.6)** | **9.4 (2.3 , 20.2)** | **184 (48 , 393)** | **4 (1 , 8.4)** | **5.2 (1.4 , 11.1)** |
| **Eastern Europe** | **6118 (1414 , 13573)** | **2.6 (0.6 , 5.7)** | **4.8 (1.1 , 10.6)** | **5904 (1469 , 12740)** | **2.9 (0.7 , 6.2)** | **2.6 (0.7 , 5.7)** |
| **Belarus** | **261 (56 , 618)** | **2.3 (0.5 , 5.2)** | **4.5 (1 , 10.5)** | **202 (48 , 460)** | **2.4 (0.6 , 5.2)** | **1.9 (0.5 , 4.5)** |
| **Estonia** | **69 (16 , 158)** | **3.5 (0.8 , 7.7)** | **7 (1.6 , 16)** | **70 (18 , 157)** | **3.8 (1 , 8.1)** | **3.8 (1 , 8.5)** |
| **Latvia** | **97 (22 , 221)** | **3.3 (0.8 , 7.2)** | **6.6 (1.5 , 15.1)** | **103 (25 , 230)** | **3.9 (1 , 8)** | **3.8 (0.9 , 8.6)** |
| **Lithuania** | **118 (27 , 267)** | **2.8 (0.6 , 6.1)** | **5.5 (1.3 , 12.5)** | **99 (24 , 216)** | **2.8 (0.7 , 5.8)** | **2.5 (0.6 , 5.6)** |
| **Republic of Moldova** | **127 (31 , 274)** | **3.5 (0.8 , 7.4)** | **5.3 (1.3 , 11.4)** | **104 (26 , 226)** | **4 (1 , 8.4)** | **3 (0.7 , 6.4)** |
| **Russian Federation** | **3966 (921 , 8940)** | **2.6 (0.6 , 5.7)** | **4.7 (1.1 , 10.4)** | **4167 (1059 , 9101)** | **3 (0.7 , 6.3)** | **2.7 (0.7 , 6)** |
| **Ukraine** | **1480 (335 , 3477)** | **2.6 (0.6 , 5.6)** | **5.1 (1.2 , 12)** | **1158 (270 , 2618)** | **2.8 (0.7 , 6)** | **2.4 (0.6 , 5.5)** |
| **Central Europe** | **12038 (3014 , 25845)** | **6.1 (1.5 , 12.6)** | **12.9 (3.2 , 27.8)** | **8638 (2309 , 18569)** | **5.9 (1.6 , 11.9)** | **6.6 (1.8 , 14.2)** |
| **Albania** | **90 (18 , 219)** | **2.9 (0.6 , 6.6)** | **4.3 (0.9 , 10.6)** | **49 (12 , 114)** | **2.9 (0.7 , 6.2)** | **2.1 (0.5 , 4.9)** |
| **Bosnia and Herzegovina** | **424 (105 , 925)** | **7.7 (2 , 15.8)** | **15.9 (3.9 , 34.4)** | **286 (78 , 639)** | **7.2 (2 , 14.4)** | **8.2 (2.2 , 18.4)** |
| **Bulgaria** | **682 (167 , 1535)** | **5.3 (1.3 , 11.2)** | **10.8 (2.6 , 24.4)** | **474 (121 , 1078)** | **5.2 (1.3 , 10.7)** | **5.4 (1.4 , 12.4)** |
| **Croatia** | **514 (130 , 1140)** | **6.3 (1.6 , 13.1)** | **13.5 (3.4 , 30)** | **358 (92 , 789)** | **6.1 (1.7 , 12.4)** | **6.4 (1.7 , 14.2)** |
| **Czechia** | **1547 (422 , 3221)** | **9 (2.5 , 17.7)** | **16.4 (4.5 , 34.1)** | **1145 (315 , 2430)** | **8.5 (2.5 , 16.8)** | **8.9 (2.4 , 19)** |
| **Hungary** | **1255 (317 , 2729)** | **6.8 (1.7 , 14)** | **15.9 (4 , 34.8)** | **995 (259 , 2189)** | **6.3 (1.7 , 13)** | **8.1 (2.1 , 18)** |
| **Montenegro** | **71 (16 , 158)** | **7 (1.7 , 14.8)** | **15.9 (3.7 , 35.7)** | **46 (12 , 97)** | **7.2 (2 , 14.5)** | **8.2 (2.2 , 17.3)** |
| **North Macedonia** | **233 (58 , 517)** | **7 (1.8 , 14.4)** | **15.2 (3.9 , 33.6)** | **151 (41 , 327)** | **7.1 (2 , 14.2)** | **9 (2.4 , 19.3)** |
| **Poland** | **4214 (998 , 9455)** | **6.1 (1.5 , 12.8)** | **14.3 (3.4 , 32)** | **3051 (809 , 6525)** | **5.8 (1.5 , 11.9)** | **7 (1.9 , 15.2)** |
| **Romania** | **1284 (298 , 2861)** | **4.1 (1 , 8.9)** | **8 (1.9 , 17.9)** | **834 (208 , 1815)** | **3.9 (1 , 8.3)** | **3.7 (0.9 , 8.1)** |
| **Serbia** | **1169 (290 , 2571)** | **7.2 (1.9 , 14.8)** | **15.7 (3.9 , 34.5)** | **840 (220 , 1857)** | **6.9 (1.9 , 13.8)** | **9.2 (2.4 , 20.6)** |
| **Slovakia** | **378 (92 , 857)** | **4.8 (1.1 , 10.1)** | **9.9 (2.4 , 22.2)** | **278 (71 , 641)** | **4.7 (1.2 , 9.7)** | **4.9 (1.3 , 11.4)** |
| **Slovenia** | **177 (42 , 396)** | **4.8 (1.2 , 10.2)** | **9.3 (2.2 , 20.7)** | **132 (33 , 291)** | **5 (1.3 , 10.4)** | **4.8 (1.2 , 10.8)** |
| **Central Asia** | **1486 (353 , 3213)** | **3.1 (0.7 , 6.8)** | **5.3 (1.3 , 11.4)** | **1515 (391 , 3137)** | **3.7 (1 , 7.6)** | **3.9 (1 , 8)** |
| **Armenia** | **155 (36 , 348)** | **4.7 (1.1 , 10.2)** | **8.8 (2 , 19.8)** | **133 (34 , 284)** | **5.2 (1.4 , 10.9)** | **5.5 (1.4 , 11.6)** |
| **Azerbaijan** | **223 (46 , 524)** | **3.2 (0.7 , 7.2)** | **5.6 (1.2 , 12.8)** | **188 (48 , 410)** | **3.8 (1 , 7.9)** | **3.9 (1 , 8.5)** |
| **Georgia** | **250 (59 , 549)** | **5.2 (1.3 , 11.2)** | **10.2 (2.4 , 22.5)** | **182 (48 , 385)** | **5.2 (1.3 , 10.8)** | **5.1 (1.3 , 10.8)** |
| **Kazakhstan** | **411 (97 , 911)** | **3.7 (0.9 , 8)** | **6.4 (1.5 , 14)** | **496 (132 , 1037)** | **5 (1.3 , 10.2)** | **5 (1.3 , 10.4)** |
| **Kyrgyzstan** | **38 (8 , 85)** | **1.7 (0.4 , 3.9)** | **2.1 (0.5 , 4.8)** | **44 (11 , 96)** | **2.2 (0.6 , 4.8)** | **1.8 (0.5 , 3.9)** |
| **Mongolia** | **30 (6 , 72)** | **0.9 (0.2 , 2)** | **4 (0.8 , 9.5)** | **22 (5 , 50)** | **0.9 (0.2 , 1.9)** | **2 (0.5 , 4.7)** |
| **Tajikistan** | **63 (14 , 142)** | **2.2 (0.5 , 4.7)** | **3.4 (0.8 , 7.5)** | **79 (20 , 171)** | **3.1 (0.8 , 6.5)** | **3.5 (0.9 , 7.5)** |
| **Turkmenistan** | **35 (8 , 83)** | **1.8 (0.4 , 3.9)** | **2.3 (0.5 , 5.2)** | **43 (11 , 96)** | **2.6 (0.6 , 5.4)** | **2 (0.5 , 4.5)** |
| **Uzbekistan** | **281 (65 , 615)** | **2.6 (0.6 , 5.5)** | **3.7 (0.9 , 7.9)** | **327 (81 , 702)** | **2.9 (0.7 , 6)** | **3.3 (0.8 , 7.1)** |
| **Central Latin America** | **4824 (1249 , 10183)** | **4.2 (1.1 , 8.5)** | **4.7 (1.2 , 9.8)** | **6125 (1687 , 12875)** | **5.3 (1.5 , 10.5)** | **4.9 (1.4 , 10.3)** |
| **Colombia** | **888 (227 , 1971)** | **3.7 (1 , 7.6)** | **3.8 (1 , 8.3)** | **1198 (307 , 2738)** | **4.7 (1.3 , 9.7)** | **4.1 (1 , 9.3)** |
| **Costa Rica** | **131 (34 , 289)** | **4 (1 , 8.1)** | **5.8 (1.5 , 12.7)** | **137 (35 , 308)** | **5 (1.3 , 10.1)** | **4.9 (1.3 , 11.1)** |
| **El Salvador** | **94 (24 , 206)** | **3.5 (0.9 , 7.3)** | **3.7 (0.9 , 8.2)** | **136 (36 , 312)** | **4.2 (1.2 , 8.6)** | **3.9 (1 , 9)** |
| **Guatemala** | **167 (42 , 363)** | **2.9 (0.8 , 5.8)** | **3.6 (0.9 , 7.8)** | **251 (67 , 552)** | **3.7 (1 , 7.3)** | **4.3 (1.2 , 9.5)** |
| **Honduras** | **130 (29 , 302)** | **3.7 (0.9 , 8.5)** | **4.9 (1.1 , 11.6)** | **202 (52 , 469)** | **4.7 (1.2 , 10.3)** | **6.9 (1.8 , 15.8)** |
| **Mexico** | **2509 (662 , 5292)** | **4.7 (1.2 , 9.4)** | **4.9 (1.3 , 10.2)** | **3143 (871 , 6515)** | **5.9 (1.7 , 11.7)** | **5.2 (1.4 , 10.6)** |
| **Nicaragua** | **73 (18 , 159)** | **3.3 (0.9 , 6.8)** | **4.3 (1.1 , 9.3)** | **100 (27 , 207)** | **4.5 (1.2 , 9.1)** | **4.5 (1.2 , 9.3)** |
| **Panama** | **87 (22 , 190)** | **4.1 (1.1 , 8.3)** | **4.4 (1.1 , 9.7)** | **99 (26 , 220)** | **5.4 (1.5 , 10.7)** | **4.6 (1.2 , 10.1)** |
| **Venezuela (Bolivarian Republic of)** | **746 (182 , 1724)** | **4.1 (1 , 8.5)** | **5.8 (1.4 , 13.2)** | **860 (224 , 2008)** | **5.2 (1.5 , 10.7)** | **5.6 (1.5 , 13.2)** |
| **Andean Latin America** | **675 (165 , 1484)** | **2.2 (0.5 , 4.5)** | **2.7 (0.6 , 5.8)** | **1019 (264 , 2245)** | **3 (0.8 , 6.2)** | **3.6 (0.9 , 7.9)** |
| **Bolivia (Plurinational State of)** | **145 (34 , 346)** | **2.2 (0.5 , 4.9)** | **3.9 (0.9 , 9.3)** | **201 (51 , 463)** | **2.7 (0.7 , 5.7)** | **4.7 (1.2 , 10.8)** |
| **Ecuador** | **221 (53 , 489)** | **2.6 (0.6 , 5.3)** | **3.4 (0.8 , 7.5)** | **353 (90 , 756)** | **3.9 (1.1 , 7.9)** | **4.7 (1.2 , 10)** |
| **Peru** | **309 (74 , 727)** | **1.9 (0.5 , 4.1)** | **2.1 (0.5 , 4.8)** | **465 (116 , 1090)** | **2.7 (0.7 , 5.6)** | **2.8 (0.7 , 6.5)** |
| **Caribbean** | **1739 (445 , 3659)** | **4.5 (1.2 , 9.2)** | **7.3 (1.9 , 15.4)** | **1807 (486 , 3849)** | **5.8 (1.6 , 11.7)** | **6.5 (1.7 , 13.8)** |
| **Antigua and Barbuda** | **2 (1 , 5)** | **3.1 (0.8 , 6.3)** | **5.5 (1.5 , 11.2)** | **5 (1 , 10)** | **7.2 (1.9 , 14.7)** | **9 (2.5 , 19.1)** |
| **Barbados** | **15 (4 , 31)** | **3.4 (0.9 , 6.9)** | **6.6 (1.7 , 14.1)** | **28 (7 , 59)** | **7.1 (1.9 , 14.1)** | **9.9 (2.6 , 21.2)** |
| **Belize** | **5 (1 , 10)** | **2.7 (0.7 , 5.8)** | **3.6 (0.9 , 7.9)** | **5 (1 , 12)** | **4.2 (1.2 , 8.6)** | **4.3 (1.2 , 9.1)** |
| **Bermuda** | **5 (1 , 11)** | **4.5 (1.1 , 9.4)** | **8.6 (2.2 , 18.2)** | **4 (1 , 10)** | **6.1 (1.6 , 12.6)** | **5.2 (1.3 , 12)** |
| **Bahamas** | **11 (3 , 23)** | **3.4 (0.9 , 7)** | **6.7 (1.7 , 14.3)** | **16 (4 , 33)** | **6 (1.5 , 12.2)** | **7.6 (2 , 16)** |
| **Cuba** | **925 (225 , 1969)** | **5.8 (1.4 , 12)** | **10.3 (2.5 , 21.8)** | **735 (192 , 1581)** | **6.3 (1.7 , 13.2)** | **6.9 (1.8 , 15)** |
| **Dominica** | **4 (1 , 8)** | **3.6 (1 , 7.1)** | **9.6 (2.6 , 19.6)** | **4 (1 , 9)** | **6.3 (1.7 , 12.6)** | **8.8 (2.3 , 18.8)** |
| **Dominican Republic** | **127 (29 , 304)** | **2.1 (0.5 , 4.6)** | **3.1 (0.7 , 7.2)** | **141 (35 , 329)** | **3 (0.8 , 6.5)** | **3 (0.7 , 7.1)** |
| **Grenada** | **4 (1 , 9)** | **4.2 (1.1 , 8.4)** | **9.3 (2.6 , 18.5)** | **6 (2 , 11)** | **6.7 (1.8 , 13.3)** | **9.7 (2.7 , 19.4)** |
| **Guyana** | **15 (4 , 32)** | **3.9 (1 , 7.9)** | **5.7 (1.5 , 11.9)** | **27 (7 , 59)** | **6.7 (1.8 , 13.2)** | **8.5 (2.3 , 18.4)** |
| **Haiti** | **119 (26 , 294)** | **2.4 (0.6 , 5.4)** | **4.3 (0.9 , 10.4)** | **240 (60 , 549)** | **4.3 (1.1 , 8.9)** | **6.9 (1.8 , 15.7)** |
| **Jamaica** | **111 (28 , 241)** | **4.5 (1.1 , 9.7)** | **7.9 (2 , 17.3)** | **126 (34 , 276)** | **6.6 (1.8 , 13.4)** | **7.9 (2.2 , 17.5)** |
| **Puerto Rico** | **234 (63 , 506)** | **6.2 (1.7 , 12.2)** | **7.1 (1.9 , 15.4)** | **279 (76 , 612)** | **8.9 (2.5 , 17.5)** | **6.5 (1.7 , 14.3)** |
| **Saint Kitts and Nevis** | **2 (1 , 4)** | **3.6 (1 , 7.5)** | **7.7 (2.1 , 15.7)** | **3 (1 , 6)** | **6.7 (1.8 , 13.6)** | **8.9 (2.4 , 19.3)** |
| **Saint Lucia** | **7 (2 , 14)** | **3.6 (1 , 7.3)** | **7.1 (2 , 14.5)** | **10 (3 , 21)** | **8.5 (2.5 , 16.2)** | **8.9 (2.6 , 18.5)** |
| **Saint Vincent and the Grenadines** | **4 (1 , 8)** | **3.2 (0.8 , 6.5)** | **5.9 (1.6 , 12.1)** | **6 (2 , 13)** | **7 (1.9 , 13.7)** | **9.3 (2.6 , 19.3)** |
| **Suriname** | **21 (6 , 44)** | **5.6 (1.5 , 11.2)** | **8.2 (2.2 , 17.1)** | **25 (7 , 53)** | **7.3 (2.1 , 14.4)** | **7.8 (2.2 , 16.4)** |
| **Trinidad and Tobago** | **60 (16 , 132)** | **5.5 (1.5 , 10.9)** | **6.9 (1.9 , 15.2)** | **76 (20 , 167)** | **8.2 (2.3 , 16.1)** | **7.8 (2 , 17.1)** |
| **United States Virgin Islands** | **10 (3 , 21)** | **4.8 (1.2 , 9.8)** | **11.9 (3.1 , 24.9)** | **10 (3 , 22)** | **8.3 (2.3 , 16.5)** | **10 (2.7 , 21)** |
| **Tropical Latin America** | **4455 (1131 , 9401)** | **3.1 (0.8 , 6.4)** | **4.4 (1.1 , 9.2)** | **5306 (1450 , 11124)** | **4.2 (1.1 , 8.6)** | **4 (1.1 , 8.4)** |
| **Brazil** | **4339 (1102 , 9141)** | **3 (0.8 , 6.4)** | **4.4 (1.1 , 9.2)** | **5195 (1417 , 10857)** | **4.2 (1.1 , 8.7)** | **4 (1.1 , 8.4)** |
| **Paraguay** | **116 (27 , 263)** | **3.5 (0.8 , 7.3)** | **4.8 (1.1 , 10.9)** | **111 (28 , 254)** | **4 (1 , 8.2)** | **3.9 (1 , 9)** |
| **East Asia** | **58813 (13023 , 134110)** | **3.3 (0.7 , 7.2)** | **6.6 (1.5 , 14.9)** | **35707 (8731 , 80549)** | **3.5 (0.9 , 7.6)** | **3.3 (0.8 , 7.5)** |
| **China** | **56720 (12510 , 129336)** | **3.2 (0.7 , 7.1)** | **6.6 (1.5 , 14.9)** | **33935 (8194 , 77063)** | **3.5 (0.9 , 7.5)** | **3.3 (0.8 , 7.5)** |
| **Democratic People's Republic of Korea** | **566 (115 , 1331)** | **2.5 (0.5 , 5.7)** | **4.8 (1 , 11)** | **540 (138 , 1234)** | **3 (0.8 , 6.4)** | **2.8 (0.7 , 6.4)** |
| **Taiwan (Province of China)** | **1526 (354 , 3372)** | **4.4 (1.1 , 9.2)** | **8.4 (1.9 , 18.5)** | **1232 (318 , 2707)** | **5.6 (1.5 , 11.5)** | **5.7 (1.5 , 12.5)** |
| **Southeast Asia** | **12833 (2963 , 28299)** | **3.7 (0.9 , 7.8)** | **5.5 (1.3 , 11.9)** | **13278 (3562 , 28856)** | **4.2 (1.1 , 8.7)** | **4.4 (1.2 , 9.5)** |
| **Cambodia** | **322 (73 , 716)** | **4.3 (1 , 9.3)** | **8 (1.9 , 17.5)** | **266 (71 , 578)** | **3.8 (1 , 7.9)** | **4.2 (1.1 , 9)** |
| **Indonesia** | **4112 (899 , 9600)** | **3.5 (0.8 , 7.7)** | **4.9 (1.1 , 11.2)** | **3920 (934 , 9512)** | **3.4 (0.9 , 7.5)** | **3.8 (0.9 , 9.3)** |
| **Lao People's Democratic Republic** | **112 (26 , 256)** | **4.4 (1 , 9.5)** | **6.5 (1.6 , 14.8)** | **112 (30 , 247)** | **4.6 (1.2 , 9.7)** | **5.5 (1.5 , 12)** |
| **Malaysia** | **829 (198 , 1815)** | **5 (1.3 , 10.4)** | **7.2 (1.8 , 15.7)** | **872 (231 , 1910)** | **5.9 (1.6 , 12)** | **7.4 (2 , 16.1)** |
| **Maldives** | **4 (1 , 9)** | **3.5 (0.9 , 7.2)** | **3.3 (0.8 , 7.1)** | **4 (1 , 9)** | **4.5 (1.2 , 9.2)** | **3.6 (1 , 7.8)** |
| **Mauritius** | **58 (16 , 120)** | **7.6 (2.1 , 14.9)** | **7.9 (2.2 , 16.4)** | **71 (19 , 149)** | **9.3 (2.6 , 18)** | **7.4 (2 , 15.6)** |
| **Myanmar** | **1025 (228 , 2489)** | **3.9 (0.9 , 8.9)** | **6 (1.4 , 14.4)** | **1279 (342 , 2706)** | **4.8 (1.3 , 9.9)** | **5.3 (1.4 , 11.1)** |
| **Philippines** | **1191 (276 , 2824)** | **2.9 (0.7 , 6.3)** | **3.9 (0.9 , 9.1)** | **1681 (427 , 3723)** | **4.2 (1.1 , 8.6)** | **4.4 (1.1 , 9.7)** |
| **Sri Lanka** | **463 (120 , 1039)** | **4.9 (1.3 , 9.9)** | **4.5 (1.2 , 9.8)** | **628 (169 , 1384)** | **6.7 (1.9 , 13)** | **4.6 (1.2 , 10.1)** |
| **Seychelles** | **5 (1 , 11)** | **5.1 (1.4 , 10)** | **12.8 (3.5 , 25.8)** | **6 (2 , 12)** | **8.1 (2.3 , 15.8)** | **10.8 (3.1 , 22.3)** |
| **Thailand** | **2263 (525 , 5253)** | **3.5 (0.8 , 7.5)** | **5.2 (1.2 , 12)** | **2127 (535 , 4899)** | **4.2 (1.1 , 8.8)** | **3.8 (1 , 8.8)** |
| **Timor-Leste** | **17 (4 , 40)** | **4 (1 , 8.9)** | **4.9 (1.1 , 11)** | **15 (4 , 33)** | **3.8 (1 , 8.1)** | **3.9 (1 , 8.8)** |
| **Viet Nam** | **2416 (553 , 5401)** | **3.8 (0.9 , 8.4)** | **7.4 (1.7 , 16.3)** | **2278 (614 , 4993)** | **4.7 (1.3 , 9.7)** | **4.6 (1.2 , 10)** |
| **Oceania** | **218 (50 , 495)** | **5.2 (1.2 , 11.1)** | **7.2 (1.7 , 15.9)** | **268 (64 , 606)** | **5.9 (1.4 , 12.2)** | **7.8 (2 , 17.4)** |
| **American Samoa** | **3 (1 , 6)** | **8.8 (2.5 , 16.9)** | **15 (4.2 , 29.3)** | **4 (1 , 8)** | **11.5 (3.5 , 22)** | **15.9 (4.7 , 32.1)** |
| **Cook Islands** | **1 (0 , 3)** | **6.6 (1.5 , 13.6)** | **11.2 (2.7 , 23.7)** | **1 (0 , 3)** | **11.3 (3.1 , 22.1)** | **10.3 (2.7 , 22)** |
| **Micronesia (Federated States of)** | **3 (1 , 8)** | **5.8 (1.4 , 12.5)** | **11.2 (2.6 , 26.7)** | **4 (1 , 10)** | **7.3 (2 , 15.5)** | **12.2 (3.2 , 27.7)** |
| **Fiji** | **23 (7 , 47)** | **6.7 (2 , 13)** | **8.4 (2.5 , 16.6)** | **41 (11 , 89)** | **8.8 (2.5 , 17.4)** | **11 (3.1 , 23.9)** |
| **Guam** | **7 (2 , 15)** | **5.6 (1.3 , 11.9)** | **8.1 (2 , 17.8)** | **6 (2 , 13)** | **6.9 (1.9 , 14)** | **5.8 (1.5 , 12.7)** |
| **Kiribati** | **3 (1 , 6)** | **4.7 (1.2 , 9.7)** | **10.2 (2.5 , 22.1)** | **3 (1 , 6)** | **3.6 (0.9 , 7.7)** | **7.2 (1.8 , 16.1)** |
| **Marshall Islands** | **2 (0 , 5)** | **7.6 (1.9 , 16.5)** | **13.7 (3.2 , 32.2)** | **2 (1 , 5)** | **9.2 (2.7 , 18.4)** | **15.5 (4.5 , 33)** |
| **Nauru** | **0 (0 , 0)** | **5 (1.2 , 11)** | **13 (3 , 30)** | **0 (0 , 1)** | **5.7 (1.5 , 12.1)** | **11.4 (2.9 , 25.8)** |
| **Niue** | **0 (0 , 0)** | **8.7 (2.3 , 17.3)** | **14.1 (3.7 , 28.7)** | **0 (0 , 0)** | **11.3 (3.4 , 21.9)** | **13.2 (3.7 , 28.2)** |
| **Northern Mariana Islands** | **3 (1 , 7)** | **6.9 (1.6 , 14.6)** | **15.2 (3.7 , 31.8)** | **2 (1 , 5)** | **7.1 (2 , 14.3)** | **9.9 (2.7 , 20.6)** |
| **Palau** | **1 (0 , 2)** | **5.5 (1.2 , 11.5)** | **10.2 (2.3 , 21.7)** | **2 (1 , 4)** | **9.9 (2.9 , 19.6)** | **18.5 (5.3 , 38.6)** |
| **Papua New Guinea** | **133 (29 , 322)** | **4.9 (1.1 , 10.9)** | **6.3 (1.4 , 14.8)** | **157 (34 , 363)** | **5.4 (1.2 , 11.4)** | **6.7 (1.5 , 15)** |
| **Samoa** | **3 (1 , 7)** | **4.3 (1.1 , 9)** | **4.8 (1.2 , 10.2)** | **6 (2 , 13)** | **6.9 (1.9 , 14.4)** | **8 (2.1 , 17.8)** |
| **Solomon Islands** | **14 (3 , 37)** | **5 (1.1 , 11.2)** | **10.3 (2.1 , 26.1)** | **18 (4 , 40)** | **4.7 (1.1 , 10.3)** | **10.3 (2.4 , 22.8)** |
| **Tokelau** | **0 (0 , 0)** | **6 (1.4 , 12.6)** | **6.8 (1.6 , 15.7)** | **0 (0 , 0)** | **7.7 (2.2 , 15.6)** | **10.5 (2.8 , 22.8)** |
| **Tonga** | **3 (1 , 8)** | **4.7 (1.1 , 9.7)** | **10 (2.3 , 21.9)** | **3 (1 , 8)** | **6.4 (1.8 , 13.1)** | **8.1 (2.1 , 17.7)** |
| **Tuvalu** | **0 (0 , 1)** | **5.6 (1.3 , 11.9)** | **8.5 (1.9 , 19.4)** | **1 (0 , 1)** | **7.6 (2.2 , 15.6)** | **10.2 (2.7 , 22.3)** |
| **Vanuatu** | **6 (1 , 15)** | **4.9 (1.1 , 10.7)** | **7.8 (1.7 , 17.8)** | **5 (1 , 12)** | **5.4 (1.4 , 11.2)** | **6.8 (1.8 , 15.2)** |
| **North Africa and Middle East** | **11118 (2809 , 23407)** | **4.5 (1.1 , 9.4)** | **5.7 (1.5 , 12)** | **8637 (2369 , 18121)** | **4.9 (1.3 , 9.9)** | **4.5 (1.2 , 9.3)** |
| **Afghanistan** | **220 (54 , 533)** | **2.5 (0.6 , 5.4)** | **4.7 (1.1 , 11.2)** | **290 (75 , 642)** | **2.4 (0.6 , 5)** | **4.9 (1.3 , 10.6)** |
| **Algeria** | **626 (157 , 1369)** | **4.9 (1.3 , 10.1)** | **4.2 (1.1 , 9)** | **610 (166 , 1269)** | **5.6 (1.5 , 11.1)** | **4.2 (1.2 , 8.8)** |
| **Bahrain** | **46 (13 , 94)** | **9.6 (2.8 , 18.2)** | **14.4 (4.1 , 28.5)** | **38 (11 , 74)** | **11.2 (3.5 , 21.1)** | **13.6 (4.2 , 26)** |
| **Egypt** | **1135 (277 , 2623)** | **3.1 (0.8 , 6.6)** | **3.4 (0.8 , 7.9)** | **982 (250 , 2345)** | **4.5 (1.2 , 9.7)** | **3.8 (1 , 8.9)** |
| **Iran (Islamic Republic of)** | **1334 (337 , 2791)** | **3.4 (0.9 , 7.1)** | **4.1 (1 , 8.5)** | **1355 (373 , 2752)** | **4.9 (1.4 , 9.7)** | **4 (1.1 , 8.2)** |
| **Iraq** | **745 (187 , 1598)** | **5.7 (1.4 , 11.8)** | **8.1 (2 , 16.9)** | **689 (184 , 1513)** | **6.1 (1.7 , 12.2)** | **6.2 (1.7 , 13.4)** |
| **Jordan** | **192 (48 , 411)** | **6.1 (1.6 , 12.3)** | **6.8 (1.7 , 14.5)** | **143 (37 , 306)** | **5.7 (1.5 , 11.5)** | **5.4 (1.4 , 11.3)** |
| **Kuwait** | **77 (21 , 162)** | **7.5 (2.1 , 14.6)** | **6.8 (1.9 , 14)** | **45 (12 , 96)** | **7.3 (2 , 14.6)** | **5.3 (1.5 , 10.9)** |
| **Lebanon** | **287 (73 , 607)** | **7.2 (1.9 , 14.5)** | **12.3 (3.1 , 26.1)** | **278 (73 , 599)** | **7.9 (2.2 , 15.9)** | **9.7 (2.6 , 20.9)** |
| **Libya** | **220 (55 , 474)** | **7.1 (1.9 , 14.4)** | **9.7 (2.5 , 20.9)** | **151 (39 , 326)** | **6.4 (1.8 , 13)** | **6.4 (1.7 , 13.8)** |
| **Morocco** | **798 (180 , 1848)** | **5.5 (1.3 , 11.8)** | **5.6 (1.3 , 12.9)** | **707 (181 , 1576)** | **5.3 (1.4 , 10.9)** | **4.5 (1.2 , 9.9)** |
| **Palestine** | **112 (30 , 232)** | **7.3 (1.9 , 14.5)** | **12.7 (3.4 , 26)** | **88 (24 , 184)** | **6.5 (1.8 , 13.1)** | **8.1 (2.2 , 16.7)** |
| **Oman** | **31 (8 , 67)** | **3.9 (1.1 , 8)** | **5.1 (1.4 , 10.7)** | **32 (8 , 66)** | **5.4 (1.5 , 10.9)** | **5.4 (1.5 , 11.3)** |
| **Qatar** | **42 (12 , 88)** | **7.6 (2.2 , 14.7)** | **13.2 (3.8 , 26.2)** | **31 (9 , 62)** | **11.1 (3.4 , 20.5)** | **24.1 (7.7 , 45.5)** |
| **Saudi Arabia** | **357 (93 , 764)** | **4.9 (1.3 , 9.9)** | **4.6 (1.3 , 9.6)** | **323 (87 , 689)** | **5.6 (1.5 , 11.3)** | **5.3 (1.5 , 10.9)** |
| **Sudan** | **324 (81 , 772)** | **3.2 (0.8 , 7)** | **3.7 (1 , 8.8)** | **262 (66 , 598)** | **3.7 (1 , 7.7)** | **3.3 (0.9 , 7.4)** |
| **Syrian Arab Republic** | **201 (47 , 447)** | **4.1 (1 , 8.5)** | **3.5 (0.8 , 7.8)** | **180 (47 , 395)** | **4.7 (1.3 , 9.6)** | **3.6 (0.9 , 7.7)** |
| **Tunisia** | **527 (125 , 1210)** | **8.6 (2.2 , 17.6)** | **9.2 (2.2 , 20.9)** | **259 (67 , 600)** | **6.5 (1.8 , 13.2)** | **4.1 (1.1 , 9.5)** |
| **Turkey** | **3500 (792 , 7801)** | **5.3 (1.2 , 11.5)** | **8.8 (2 , 19.6)** | **1934 (498 , 4191)** | **5 (1.3 , 10.2)** | **4.2 (1.1 , 9.1)** |
| **United Arab Emirates** | **187 (48 , 408)** | **4.7 (1.3 , 9.5)** | **13 (3.6 , 26.9)** | **93 (24 , 197)** | **6.6 (1.8 , 13.2)** | **13.1 (3.7 , 27.1)** |
| **Yemen** | **148 (34 , 359)** | **2.1 (0.5 , 4.7)** | **2.7 (0.6 , 6.5)** | **138 (35 , 323)** | **2.4 (0.6 , 5.2)** | **2.2 (0.6 , 5.1)** |
| **South Asia** | **17121 (4191 , 37358)** | **2.7 (0.7 , 5.7)** | **2.7 (0.7 , 5.9)** | **21869 (5796 , 47107)** | **3.6 (0.9 , 7.5)** | **3.2 (0.9 , 7)** |
| **Bangladesh** | **1043 (217 , 2737)** | **1.7 (0.4 , 3.9)** | **1.6 (0.3 , 4.2)** | **1197 (291 , 2705)** | **2.7 (0.7 , 5.7)** | **2 (0.5 , 4.6)** |
| **Bhutan** | **6 (1 , 13)** | **2.2 (0.5 , 4.9)** | **2.2 (0.5 , 5)** | **7 (2 , 15)** | **3 (0.8 , 6.5)** | **2.6 (0.6 , 5.8)** |
| **India** | **13444 (3353 , 29505)** | **2.9 (0.7 , 6)** | **2.7 (0.7 , 5.8)** | **16776 (4418 , 36498)** | **3.6 (1 , 7.5)** | **3 (0.8 , 6.6)** |
| **Nepal** | **211 (48 , 481)** | **1.9 (0.4 , 4.1)** | **2.2 (0.5 , 5)** | **302 (74 , 681)** | **3 (0.8 , 6.3)** | **2.8 (0.7 , 6.2)** |
| **Pakistan** | **2417 (534 , 5608)** | **2.7 (0.7 , 5.9)** | **4.9 (1.1 , 11.1)** | **3587 (858 , 8358)** | **3.9 (0.9 , 8.4)** | **7.1 (1.7 , 16.4)** |
| **Southern Sub-Saharan Africa** | **1136 (281 , 2407)** | **3 (0.8 , 6.4)** | **5.7 (1.4 , 11.9)** | **1750 (467 , 3601)** | **4.8 (1.3 , 9.7)** | **5.8 (1.5 , 11.9)** |
| **Botswana** | **33 (8 , 74)** | **3.2 (0.8 , 7.1)** | **7.3 (1.7 , 15.9)** | **47 (12 , 106)** | **4.5 (1.2 , 9.5)** | **7 (1.9 , 15.4)** |
| **Lesotho** | **24 (5 , 57)** | **2.1 (0.5 , 4.7)** | **5.5 (1.3 , 12.9)** | **35 (8 , 83)** | **3.3 (0.9 , 7.1)** | **5.2 (1.3 , 12.1)** |
| **Namibia** | **14 (3 , 31)** | **1.8 (0.4 , 3.9)** | **2.8 (0.7 , 6.1)** | **31 (8 , 71)** | **3.8 (0.9 , 7.9)** | **4.1 (1 , 9.2)** |
| **South Africa** | **939 (232 , 1994)** | **3.3 (0.8 , 6.9)** | **5.9 (1.4 , 12.3)** | **1338 (360 , 2786)** | **5.1 (1.4 , 10.3)** | **5.5 (1.5 , 11.4)** |
| **Eswatini** | **14 (3 , 34)** | **2.6 (0.6 , 5.7)** | **7.7 (1.8 , 17.8)** | **18 (4 , 41)** | **4.2 (1.1 , 8.8)** | **5.9 (1.5 , 13.3)** |
| **Zimbabwe** | **112 (28 , 243)** | **2.1 (0.6 , 4.5)** | **4.7 (1.2 , 10.2)** | **280 (73 , 615)** | **4 (1.1 , 8.2)** | **7.8 (2.1 , 17)** |
| **Western Sub-Saharan Africa** | **1762 (440 , 3865)** | **1.8 (0.4 , 3.8)** | **2.5 (0.6 , 5.5)** | **2413 (570 , 5300)** | **2.5 (0.6 , 5.4)** | **2.9 (0.7 , 6.4)** |
| **Benin** | **57 (13 , 128)** | **2.1 (0.5 , 4.5)** | **3.2 (0.8 , 7.1)** | **65 (16 , 139)** | **2.6 (0.7 , 5.4)** | **2.9 (0.7 , 6.1)** |
| **Burkina Faso** | **88 (21 , 202)** | **1.8 (0.4 , 4)** | **2.7 (0.7 , 6.1)** | **116 (29 , 252)** | **2.2 (0.5 , 4.7)** | **2.7 (0.7 , 5.9)** |
| **Cameroon** | **176 (43 , 400)** | **2.4 (0.6 , 5.2)** | **4 (1 , 9)** | **189 (45 , 430)** | **2.7 (0.7 , 5.9)** | **3.6 (0.9 , 8.2)** |
| **Cabo Verde** | **9 (2 , 21)** | **2.2 (0.5 , 4.9)** | **6.1 (1.5 , 13.9)** | **14 (4 , 29)** | **4.7 (1.3 , 9.7)** | **5.6 (1.5 , 11.8)** |
| **Chad** | **67 (16 , 158)** | **1.9 (0.4 , 4.3)** | **2.7 (0.6 , 6.4)** | **52 (13 , 119)** | **1.9 (0.5 , 4)** | **2.4 (0.6 , 5.3)** |
| **CÃ´te d'Ivoire** | **140 (33 , 320)** | **2.1 (0.5 , 4.7)** | **3.5 (0.8 , 8)** | **132 (34 , 292)** | **2.7 (0.7 , 5.7)** | **3.2 (0.8 , 7)** |
| **Gambia** | **9 (2 , 19)** | **1.5 (0.4 , 3.2)** | **2.3 (0.6 , 5)** | **11 (3 , 26)** | **2.4 (0.6 , 5.4)** | **2.5 (0.6 , 5.6)** |
| **Ghana** | **206 (52 , 448)** | **2.4 (0.6 , 5.1)** | **3.7 (1 , 8.1)** | **355 (86 , 777)** | **3.6 (0.9 , 7.8)** | **4.5 (1.1 , 9.7)** |
| **Guinea** | **71 (17 , 162)** | **1.5 (0.4 , 3.3)** | **3 (0.7 , 6.8)** | **67 (17 , 149)** | **1.8 (0.5 , 4)** | **2.7 (0.7 , 6)** |
| **Guinea-Bissau** | **9 (2 , 21)** | **1.9 (0.4 , 4.1)** | **3.6 (0.8 , 8.4)** | **11 (3 , 25)** | **2.2 (0.6 , 4.8)** | **3.2 (0.8 , 7.4)** |
| **Liberia** | **25 (6 , 56)** | **2.4 (0.6 , 5.1)** | **3.2 (0.8 , 7.1)** | **29 (7 , 67)** | **2.9 (0.8 , 6.3)** | **3.5 (0.9 , 7.8)** |
| **Mali** | **102 (24 , 233)** | **2 (0.5 , 4.3)** | **2.9 (0.7 , 6.5)** | **100 (24 , 224)** | **2.4 (0.6 , 5.2)** | **2.7 (0.7 , 6.1)** |
| **Mauritania** | **16 (4 , 37)** | **1.6 (0.4 , 3.5)** | **1.7 (0.4 , 4)** | **27 (7 , 61)** | **2.6 (0.7 , 5.6)** | **3 (0.8 , 6.7)** |
| **Niger** | **44 (9 , 109)** | **1.2 (0.3 , 2.9)** | **1.5 (0.3 , 3.7)** | **45 (10 , 108)** | **1.3 (0.3 , 2.9)** | **1.4 (0.3 , 3.3)** |
| **Nigeria** | **560 (123 , 1316)** | **1.4 (0.3 , 3.4)** | **1.7 (0.4 , 4)** | **984 (216 , 2277)** | **2.4 (0.6 , 5.2)** | **2.6 (0.6 , 5.9)** |
| **Sao Tome and Principe** | **2 (1 , 5)** | **3.2 (0.8 , 6.8)** | **6.1 (1.5 , 13.4)** | **2 (0 , 5)** | **2.7 (0.7 , 6.2)** | **4.1 (1 , 9.8)** |
| **Senegal** | **129 (32 , 292)** | **3 (0.8 , 6.5)** | **4.3 (1.1 , 9.6)** | **147 (37 , 319)** | **3.7 (1 , 7.8)** | **4.2 (1.1 , 9.2)** |
| **Sierra Leone** | **23 (5 , 54)** | **1.2 (0.3 , 2.7)** | **1.6 (0.4 , 3.7)** | **32 (8 , 73)** | **1.7 (0.4 , 3.8)** | **2 (0.5 , 4.6)** |
| **Togo** | **27 (6 , 63)** | **1.5 (0.3 , 3.3)** | **2.5 (0.6 , 5.8)** | **37 (9 , 84)** | **1.8 (0.5 , 4.1)** | **2.1 (0.5 , 4.7)** |
| **Eastern Sub-Saharan Africa** | **1225 (301 , 2708)** | **1.3 (0.3 , 2.9)** | **2 (0.5 , 4.4)** | **1458 (354 , 3212)** | **1.5 (0.4 , 3.2)** | **2 (0.5 , 4.3)** |
| **Burundi** | **32 (7 , 77)** | **1.2 (0.3 , 2.6)** | **1.7 (0.4 , 4.1)** | **31 (7 , 75)** | **1.2 (0.3 , 2.6)** | **1.6 (0.4 , 3.8)** |
| **Comoros** | **4 (1 , 8)** | **1.5 (0.4 , 3.3)** | **1.9 (0.4 , 4.2)** | **5 (1 , 11)** | **1.6 (0.4 , 3.6)** | **2 (0.5 , 4.5)** |
| **Djibouti** | **7 (2 , 17)** | **1.6 (0.4 , 3.6)** | **2.8 (0.7 , 7)** | **6 (1 , 13)** | **1.7 (0.4 , 3.8)** | **2.6 (0.6 , 5.8)** |
| **Eritrea** | **19 (4 , 44)** | **1.3 (0.3 , 2.9)** | **2.3 (0.5 , 5.3)** | **33 (8 , 77)** | **1.5 (0.4 , 3.5)** | **2.5 (0.6 , 5.7)** |
| **Ethiopia** | **234 (53 , 568)** | **1.2 (0.3 , 2.8)** | **1.3 (0.3 , 3.2)** | **259 (61 , 607)** | **1.3 (0.3 , 2.7)** | **1.5 (0.4 , 3.4)** |
| **Kenya** | **139 (33 , 315)** | **1.2 (0.3 , 2.7)** | **1.8 (0.4 , 3.9)** | **161 (37 , 384)** | **1.3 (0.3 , 3)** | **1.6 (0.4 , 3.6)** |
| **Madagascar** | **57 (13 , 139)** | **1.2 (0.3 , 2.6)** | **1.5 (0.3 , 3.5)** | **81 (19 , 198)** | **1.3 (0.3 , 2.9)** | **1.7 (0.4 , 3.9)** |
| **Malawi** | **72 (18 , 160)** | **1.5 (0.4 , 3.2)** | **2.9 (0.7 , 6.4)** | **82 (20 , 180)** | **1.6 (0.4 , 3.6)** | **2.3 (0.6 , 5)** |
| **Mozambique** | **107 (26 , 242)** | **1.7 (0.4 , 3.9)** | **2.9 (0.7 , 6.6)** | **125 (30 , 288)** | **1.7 (0.4 , 3.7)** | **2.4 (0.6 , 5.4)** |
| **Rwanda** | **43 (10 , 105)** | **1.4 (0.3 , 3.1)** | **2.3 (0.5 , 5.4)** | **65 (16 , 146)** | **1.7 (0.4 , 3.6)** | **2.2 (0.5 , 4.8)** |
| **Somalia** | **35 (7 , 91)** | **1 (0.2 , 2.3)** | **1.7 (0.3 , 4.3)** | **45 (10 , 121)** | **1 (0.2 , 2.3)** | **1.4 (0.3 , 3.7)** |
| **South Sudan** | **37 (8 , 88)** | **1.7 (0.4 , 3.7)** | **2.3 (0.5 , 5.4)** | **27 (6 , 65)** | **1.4 (0.3 , 3.1)** | **1.8 (0.4 , 4.1)** |
| **United Republic of Tanzania** | **202 (46 , 484)** | **1.3 (0.3 , 2.9)** | **2.1 (0.5 , 5)** | **237 (58 , 520)** | **1.5 (0.4 , 3.3)** | **2.1 (0.5 , 4.6)** |
| **Uganda** | **155 (37 , 338)** | **1.4 (0.4 , 3)** | **3.2 (0.8 , 7)** | **220 (54 , 484)** | **2.1 (0.5 , 4.5)** | **3.1 (0.8 , 6.8)** |
| **Zambia** | **81 (19 , 183)** | **1.7 (0.4 , 3.7)** | **3.3 (0.8 , 7.3)** | **78 (19 , 178)** | **1.6 (0.4 , 3.6)** | **2.6 (0.6 , 5.8)** |
| **Central Sub-Saharan Africa** | **795 (180 , 2051)** | **2.8 (0.7 , 6.7)** | **4.3 (1 , 10.9)** | **696 (170 , 1597)** | **2.4 (0.6 , 5.2)** | **2.7 (0.7 , 6.2)** |
| **Angola** | **188 (44 , 415)** | **2.9 (0.7 , 6.2)** | **4.8 (1.1 , 10.4)** | **138 (34 , 313)** | **2.2 (0.5 , 4.8)** | **2.7 (0.7 , 6)** |
| **Central African Republic** | **32 (6 , 91)** | **2.4 (0.5 , 5.9)** | **4.1 (0.9 , 10.7)** | **25 (6 , 61)** | **1.9 (0.4 , 4.3)** | **2.4 (0.6 , 5.6)** |
| **Congo** | **52 (12 , 116)** | **3.3 (0.8 , 7.1)** | **5.3 (1.3 , 11.5)** | **54 (12 , 126)** | **3.1 (0.8 , 6.8)** | **4.5 (1.1 , 10.4)** |
| **Democratic Republic of the Congo** | **479 (101 , 1417)** | **2.7 (0.6 , 6.9)** | **3.9 (0.8 , 11.2)** | **443 (105 , 1033)** | **2.3 (0.6 , 5)** | **2.6 (0.6 , 5.9)** |
| **Equatorial Guinea** | **10 (2 , 24)** | **3.6 (0.9 , 7.9)** | **6.5 (1.6 , 15)** | **11 (3 , 28)** | **3.6 (0.9 , 7.9)** | **4.8 (1.1 , 11.8)** |
| **Gabon** | **35 (8 , 79)** | **4.3 (1.1 , 9.4)** | **8.6 (2.1 , 19.2)** | **25 (6 , 58)** | **4 (1 , 8.7)** | **5 (1.2 , 11.4)** |
