# Supplementary material for: Global, Regional and National Burden of Cancers Attributable to High Fasting Plasma Glucose in 204 Countries and Territories, 1990-2019
Source: Front Endocrinol (Lausanne). 2022 Jul 19;13:879890. doi: 10.3389/fendo.2022.879890 (PMC9366927; doi:10.3389/fendo.2022.879890)
Supplement: Supplementary Table 2 — Table S2: Number, proportion and age-standardized rates of cancer deaths attributable to high fasting plasma glucose (per 100,000) in 1990 and 2019, by location (Generated from data available from http://ghdx.healthdata.org/gbd-results-tool) [file Table_2.doc]

| **Table S2: Number, proportion and age-standardised rates of cancer deaths attributable to high fasting plasma glucose (per 100,000) in 1990 and 2019, by location (Generated from data available from http://ghdx.healthdata.org/gbd-results-tool)** | | | | | | | |
| --- | --- | --- | --- | --- | --- | --- | --- |
|  | **1990** | | | **2019** | | | **% change in ASRs per 100,000**  **1990-2019** |
|  | **No**  **(95% UI)** | **PAF**  **(95% UI)** | **ASRs per 100,000 (95% UI)** | **No**  **(95% UI)** | **PAF**  **(95% UI)** | **ASRs per 100,000 (95% UI)** |
| **Global** | **150100 (39209 , 312371)** | **2.6 (0.7 , 5.4)** | **4.1 (1.1 , 8.5)** | **419338 (115729 , 848484)** | **4.2 (1.1 , 8.4)** | **5.3 (1.5 , 10.6)** | **27.8 (20.5 , 38.7)** |
| **High-income North America** | **29867 (7965 , 60987)** | **5 (1.3 , 10.3)** | **8.3 (2.2 , 17)** | **62291 (17929 , 122539)** | **7.2 (2.1 , 14.1)** | **9.5 (2.7 , 18.8)** | **14.6 (7.9 , 27)** |
| **Canada** | **1454 (375 , 3030)** | **2.7 (0.7 , 5.6)** | **4.4 (1.1 , 9.1)** | **4149 (1122 , 8614)** | **4.5 (1.2 , 9.2)** | **5.7 (1.5 , 11.8)** | **30 (17.2 , 48.9)** |
| **Greenland** | **2 (0 , 4)** | **1.7 (0.4 , 3.7)** | **5.6 (1.4 , 12.2)** | **7 (2 , 16)** | **4.6 (1.2 , 9.6)** | **11.2 (3 , 24.4)** | **101.6 (65.9 , 149.2)** |
| **United States of America** | **28411 (7582 , 57837)** | **5.3 (1.4 , 10.8)** | **8.7 (2.3 , 17.8)** | **58134 (16747 , 114100)** | **7.5 (2.2 , 14.7)** | **10 (2.9 , 19.6)** | **14.6 (7.6 , 27.3)** |
| **Australasia** | **962 (249 , 2003)** | **2.5 (0.6 , 5.3)** | **4.1 (1.1 , 8.5)** | **2573 (698 , 5274)** | **4 (1.1 , 8.2)** | **4.8 (1.3 , 9.9)** | **18 (8.3 , 33.5)** |
| **Australia** | **798 (207 , 1666)** | **2.5 (0.7 , 5.3)** | **4.1 (1.1 , 8.5)** | **2173 (588 , 4444)** | **4 (1.1 , 8.3)** | **4.8 (1.3 , 9.9)** | **18.2 (7.5 , 35.3)** |
| **New Zealand** | **163 (42 , 342)** | **2.4 (0.6 , 5.1)** | **4.1 (1.1 , 8.6)** | **400 (111 , 820)** | **3.8 (1 , 7.8)** | **4.8 (1.3 , 9.9)** | **16.9 (7.6 , 32.5)** |
| **High-income Asia Pacific** | **7403 (1910 , 15444)** | **2.5 (0.6 , 5.2)** | **3.8 (1 , 8)** | **20869 (5468 , 43482)** | **3.8 (1 , 7.7)** | **4 (1.1 , 8.4)** | **5.8 (-1.6 , 12.5)** |
| **Brunei Darussalam** | **14 (4 , 27)** | **6.4 (2 , 12.3)** | **18.1 (5.5 , 35.4)** | **45 (14 , 86)** | **8.5 (2.6 , 15.8)** | **19.9 (6.2 , 37.4)** | **9.8 (-7.4 , 33.2)** |
| **Japan** | **6349 (1638 , 13254)** | **2.6 (0.7 , 5.5)** | **3.8 (1 , 8)** | **15794 (4102 , 32973)** | **3.6 (0.9 , 7.3)** | **3.7 (1 , 7.8)** | **-1.8 (-9.4 , 4.6)** |
| **Singapore** | **155 (41 , 319)** | **4.6 (1.2 , 9.3)** | **7.8 (2.1 , 16)** | **363 (98 , 750)** | **5.2 (1.4 , 10.6)** | **4.9 (1.3 , 10.1)** | **-37.4 (-44.1 , -30.6)** |
| **Republic of Korea** | **886 (229 , 1886)** | **1.8 (0.5 , 3.8)** | **3.3 (0.9 , 7)** | **4667 (1218 , 9588)** | **4.5 (1.2 , 9.1)** | **5.3 (1.4 , 10.8)** | **60.1 (42.4 , 82)** |
| **Western Europe** | **37979 (10073 , 77067)** | **3.9 (1 , 7.9)** | **6.3 (1.7 , 12.8)** | **74827 (21400 , 148875)** | **5.8 (1.7 , 11.5)** | **7.5 (2.1 , 15.1)** | **19.6 (13.1 , 31.4)** |
| **Andorra** | **3 (1 , 6)** | **2.7 (0.7 , 5.6)** | **5.3 (1.3 , 12.4)** | **10 (3 , 22)** | **4.4 (1.2 , 9)** | **7 (1.8 , 15.3)** | **33.2 (-3.5 , 84.2)** |
| **Austria** | **519 (135 , 1088)** | **2.5 (0.7 , 5.3)** | **4.2 (1.1 , 8.8)** | **1023 (284 , 2097)** | **4.6 (1.3 , 9.4)** | **5.4 (1.5 , 11)** | **29 (17.9 , 46.2)** |
| **Belgium** | **1052 (261 , 2236)** | **3.5 (0.9 , 7.4)** | **6.6 (1.6 , 14)** | **1740 (486 , 3552)** | **5.1 (1.4 , 10.5)** | **7 (1.9 , 14.4)** | **6.9 (-2.5 , 22.2)** |
| **Cyprus** | **53 (15 , 105)** | **5.6 (1.6 , 11)** | **6.8 (2 , 13.5)** | **163 (45 , 326)** | **7.1 (2 , 14)** | **8.3 (2.3 , 16.6)** | **21.8 (3.9 , 42.9)** |
| **Denmark** | **365 (93 , 778)** | **2.4 (0.6 , 5.1)** | **4.3 (1.1 , 9.2)** | **853 (238 , 1766)** | **4.6 (1.3 , 9.5)** | **6.9 (1.9 , 14.3)** | **58.7 (42.4 , 83.5)** |
| **Finland** | **415 (112 , 846)** | **4 (1.1 , 8.2)** | **5.7 (1.5 , 11.6)** | **862 (248 , 1735)** | **5.9 (1.7 , 11.8)** | **6.4 (1.8 , 13)** | **13.7 (3.3 , 28.9)** |
| **France** | **2822 (705 , 6007)** | **1.9 (0.5 , 4.1)** | **3.2 (0.8 , 7)** | **6325 (1677 , 13273)** | **3.2 (0.9 , 6.7)** | **4.2 (1.1 , 8.9)** | **30.6 (18.5 , 46.9)** |
| **Germany** | **10071 (2730 , 20554)** | **5 (1.3 , 10.2)** | **7.6 (2 , 15.6)** | **19416 (5634 , 38269)** | **7.1 (2 , 13.9)** | **9.4 (2.7 , 18.5)** | **22.7 (10.8 , 40.5)** |
| **Greece** | **780 (194 , 1653)** | **3.3 (0.8 , 7)** | **5 (1.3 , 10.6)** | **1901 (514 , 3921)** | **5.4 (1.5 , 11.1)** | **7.3 (2 , 15.3)** | **46.6 (33.5 , 66)** |
| **Iceland** | **11 (3 , 23)** | **2.4 (0.6 , 5.1)** | **3.6 (0.9 , 7.6)** | **29 (8 , 61)** | **4.5 (1.2 , 9.1)** | **5 (1.4 , 10.5)** | **38.5 (22.8 , 60.1)** |
| **Ireland** | **139 (35 , 295)** | **1.9 (0.5 , 4)** | **3.3 (0.8 , 7)** | **513 (143 , 1049)** | **4.9 (1.4 , 10)** | **6.6 (1.8 , 13.5)** | **99.5 (79.6 , 133.4)** |
| **Israel** | **245 (66 , 509)** | **3.5 (0.9 , 7.1)** | **5 (1.3 , 10.4)** | **784 (224 , 1585)** | **5.4 (1.5 , 10.7)** | **6.5 (1.8 , 13.1)** | **29.2 (18.8 , 45)** |
| **Italy** | **6223 (1671 , 12750)** | **4.1 (1.1 , 8.5)** | **6.8 (1.8 , 13.9)** | **11866 (3408 , 23646)** | **6.2 (1.8 , 12.2)** | **7.5 (2.1 , 14.9)** | **10.8 (3.2 , 22.1)** |
| **Luxembourg** | **24 (6 , 51)** | **2.3 (0.6 , 4.9)** | **4.3 (1.1 , 9.1)** | **95 (28 , 192)** | **7.3 (2.1 , 14.4)** | **9.1 (2.6 , 18.5)** | **111.3 (81.8 , 163.6)** |
| **Malta** | **30 (8 , 61)** | **4.9 (1.3 , 9.9)** | **7 (1.9 , 14.3)** | **70 (20 , 143)** | **7 (2 , 13.9)** | **7.1 (2 , 14.4)** | **0.8 (-10.8 , 18.1)** |
| **Monaco** | **4 (1 , 9)** | **2.8 (0.7 , 5.8)** | **5 (1.2 , 11.1)** | **12 (3 , 25)** | **5.3 (1.5 , 10.7)** | **11.3 (3 , 24.2)** | **127.4 (77.6 , 203.5)** |
| **Netherlands** | **1283 (314 , 2702)** | **3.5 (0.9 , 7.4)** | **6.2 (1.5 , 13.1)** | **2444 (671 , 5095)** | **4.3 (1.2 , 8.9)** | **6.7 (1.8 , 14)** | **8.1 (-3.3 , 25)** |
| **Norway** | **408 (111 , 836)** | **3.9 (1.1 , 8)** | **5.6 (1.5 , 11.5)** | **699 (201 , 1409)** | **5.5 (1.6 , 11.1)** | **6.8 (1.9 , 13.7)** | **21 (13.2 , 33.6)** |
| **Portugal** | **769 (205 , 1577)** | **3.5 (0.9 , 7.3)** | **5.5 (1.5 , 11.3)** | **1967 (572 , 3898)** | **6.2 (1.8 , 12.2)** | **7.5 (2.2 , 15)** | **36.7 (25.1 , 56.1)** |
| **San Marino** | **2 (0 , 4)** | **2.7 (0.7 , 5.7)** | **5 (1.3 , 10.7)** | **5 (1 , 13)** | **4.7 (1.3 , 9.5)** | **7.7 (2 , 19.1)** | **56.1 (2.8 , 130.1)** |
| **Spain** | **3799 (1000 , 7662)** | **4.6 (1.2 , 9.3)** | **6.7 (1.8 , 13.6)** | **7952 (2244 , 16230)** | **6.3 (1.8 , 12.8)** | **7.6 (2.1 , 15.6)** | **13.6 (2.7 , 26.3)** |
| **Sweden** | **649 (174 , 1350)** | **3 (0.8 , 6.2)** | **4 (1.1 , 8.4)** | **1245 (353 , 2520)** | **4.7 (1.3 , 9.5)** | **5.4 (1.5 , 10.9)** | **33.5 (23 , 51.6)** |
| **Switzerland** | **575 (147 , 1182)** | **3.7 (1 , 7.7)** | **5.3 (1.3 , 10.9)** | **982 (275 , 2013)** | **4.9 (1.4 , 9.8)** | **5.2 (1.5 , 10.7)** | **-1.3 (-11.9 , 14.7)** |
| **United Kingdom** | **7707 (2089 , 15742)** | **4.5 (1.2 , 9.3)** | **8.1 (2.2 , 16.6)** | **13806 (4056 , 27143)** | **7 (2 , 13.8)** | **10.1 (3 , 20)** | **25.2 (16 , 42.5)** |
| **Southern Latin America** | **2084 (547 , 4319)** | **2.6 (0.7 , 5.3)** | **4.6 (1.2 , 9.5)** | **6034 (1689 , 12145)** | **4.8 (1.3 , 9.5)** | **7.1 (2 , 14.3)** | **53.7 (42.2 , 73.1)** |
| **Argentina** | **1624 (429 , 3367)** | **2.9 (0.7 , 6)** | **5.1 (1.4 , 10.6)** | **4221 (1168 , 8538)** | **5 (1.4 , 10.1)** | **7.6 (2.1 , 15.5)** | **50.1 (37.9 , 69.9)** |
| **Chile** | **342 (91 , 702)** | **2.1 (0.5 , 4.3)** | **3.7 (1 , 7.5)** | **1412 (405 , 2850)** | **4.5 (1.3 , 8.9)** | **5.8 (1.7 , 11.8)** | **58.7 (43 , 84.1)** |
| **Uruguay** | **117 (29 , 245)** | **1.4 (0.4 , 3)** | **2.9 (0.7 , 6.1)** | **401 (110 , 829)** | **4 (1.1 , 8.2)** | **7 (1.9 , 14.5)** | **139.5 (117.6 , 178)** |
| **Eastern Europe** | **7886 (1951 , 16688)** | **1.8 (0.4 , 3.9)** | **2.8 (0.7 , 5.9)** | **12022 (3190 , 25535)** | **2.7 (0.7 , 5.7)** | **3.4 (0.9 , 7.2)** | **21.7 (9.4 , 37.6)** |
| **Belarus** | **360 (90 , 777)** | **1.8 (0.4 , 3.9)** | **2.8 (0.7 , 5.9)** | **463 (113 , 1052)** | **2.4 (0.6 , 5)** | **2.9 (0.7 , 6.5)** | **3.8 (-18.9 , 33.8)** |
| **Estonia** | **81 (20 , 171)** | **2.3 (0.6 , 4.9)** | **3.9 (1 , 8.3)** | **140 (36 , 306)** | **3.7 (1 , 7.6)** | **5 (1.3 , 10.9)** | **26.9 (-0.8 , 62)** |
| **Latvia** | **117 (30 , 249)** | **2.1 (0.5 , 4.4)** | **3.2 (0.8 , 6.9)** | **200 (52 , 432)** | **3.6 (1 , 7.4)** | **4.8 (1.2 , 10.4)** | **48.6 (23.3 , 82.1)** |
| **Lithuania** | **136 (34 , 291)** | **1.9 (0.5 , 4.1)** | **3 (0.7 , 6.4)** | **216 (54 , 471)** | **2.8 (0.7 , 5.8)** | **3.6 (0.9 , 7.9)** | **20.5 (-2.9 , 49.1)** |
| **Republic of Moldova** | **157 (39 , 335)** | **2.5 (0.6 , 5.3)** | **3.5 (0.9 , 7.4)** | **232 (60 , 485)** | **3.7 (1 , 7.6)** | **3.9 (1 , 8.2)** | **11 (-4.3 , 30)** |
| **Russian Federation** | **4708 (1159 , 10008)** | **1.7 (0.4 , 3.6)** | **2.6 (0.6 , 5.5)** | **8133 (2124 , 17355)** | **2.7 (0.7 , 5.8)** | **3.4 (0.9 , 7.2)** | **30.2 (15.1 , 52)** |
| **Ukraine** | **2327 (581 , 4997)** | **2 (0.5 , 4.3)** | **3.2 (0.8 , 6.9)** | **2638 (676 , 5704)** | **2.7 (0.7 , 5.7)** | **3.4 (0.9 , 7.4)** | **6.8 (-10.1 , 29.1)** |
| **Central Europe** | **8901 (2306 , 18504)** | **3.5 (0.9 , 7.3)** | **6.1 (1.6 , 12.6)** | **20676 (5721 , 42693)** | **6 (1.7 , 12)** | **9.3 (2.6 , 19.2)** | **52.5 (33.6 , 75.8)** |
| **Albania** | **39 (9 , 87)** | **1.5 (0.4 , 3.3)** | **2.1 (0.5 , 4.6)** | **138 (33 , 327)** | **2.9 (0.7 , 6.3)** | **3.1 (0.7 , 7.4)** | **52 (12.6 , 103.3)** |
| **Bosnia and Herzegovina** | **181 (45 , 384)** | **3.1 (0.8 , 6.6)** | **4.8 (1.2 , 10)** | **711 (200 , 1522)** | **7.5 (2.1 , 14.8)** | **11.5 (3.3 , 24.8)** | **142.2 (89 , 218.3)** |
| **Bulgaria** | **521 (137 , 1096)** | **3 (0.8 , 6.3)** | **4.1 (1.1 , 8.6)** | **1156 (309 , 2572)** | **5.3 (1.4 , 10.7)** | **7.8 (2.1 , 17.5)** | **88.2 (49 , 137.8)** |
| **Croatia** | **435 (110 , 897)** | **3.7 (1 , 7.7)** | **7 (1.8 , 14.3)** | **871 (235 , 1905)** | **6.2 (1.7 , 12.5)** | **9.4 (2.5 , 20.6)** | **34.8 (7.3 , 72.3)** |
| **Czechia** | **1455 (390 , 2972)** | **5.1 (1.4 , 10.5)** | **10.4 (2.8 , 21.3)** | **2692 (798 , 5581)** | **8.8 (2.6 , 16.8)** | **12.1 (3.6 , 25.2)** | **16.3 (-5.7 , 47.9)** |
| **Hungary** | **1145 (297 , 2397)** | **3.7 (1 , 7.7)** | **7.8 (2 , 16.3)** | **2250 (619 , 4803)** | **6.6 (1.9 , 13.2)** | **11.2 (3.1 , 24.2)** | **44.6 (19.8 , 80)** |
| **Montenegro** | **40 (10 , 84)** | **4.2 (1.1 , 8.8)** | **6.6 (1.7 , 13.9)** | **117 (31 , 249)** | **7.1 (1.9 , 14.2)** | **11.6 (3.1 , 24.6)** | **76.1 (46.3 , 116.7)** |
| **North Macedonia** | **93 (24 , 191)** | **3.2 (0.8 , 6.7)** | **5.1 (1.4 , 10.5)** | **384 (106 , 826)** | **7.1 (2 , 13.9)** | **11.9 (3.3 , 25.6)** | **131.8 (84.9 , 199.6)** |
| **Poland** | **3032 (781 , 6335)** | **3.6 (0.9 , 7.6)** | **6.9 (1.8 , 14.5)** | **7265 (1933 , 15360)** | **6 (1.6 , 12)** | **10 (2.7 , 21.2)** | **44 (22.6 , 71.4)** |
| **Romania** | **754 (190 , 1618)** | **2.1 (0.5 , 4.6)** | **2.7 (0.7 , 5.7)** | **2118 (541 , 4531)** | **4 (1.1 , 8.4)** | **5.6 (1.4 , 12)** | **109.1 (71.7 , 160.9)** |
| **Serbia** | **723 (184 , 1524)** | **3.8 (1 , 7.9)** | **6.5 (1.7 , 13.7)** | **2009 (554 , 4340)** | **7.1 (2 , 14.1)** | **12.2 (3.3 , 26.3)** | **86.5 (49.1 , 147.3)** |
| **Slovakia** | **333 (85 , 700)** | **3.2 (0.8 , 6.6)** | **5.5 (1.4 , 11.6)** | **657 (174 , 1445)** | **4.7 (1.3 , 9.6)** | **6.9 (1.8 , 15.3)** | **25.9 (-1.8 , 64.2)** |
| **Slovenia** | **150 (38 , 332)** | **3.6 (1 , 7.5)** | **6.1 (1.6 , 13.6)** | **309 (82 , 674)** | **4.9 (1.4 , 10)** | **6.7 (1.8 , 14.7)** | **9.6 (-23.5 , 51.9)** |
| **Central Asia** | **1154 (285 , 2481)** | **1.7 (0.4 , 3.6)** | **2.5 (0.6 , 5.3)** | **3000 (802 , 6151)** | **3.4 (0.9 , 6.9)** | **4.4 (1.2 , 9.1)** | **77.7 (59 , 108.1)** |
| **Armenia** | **105 (26 , 224)** | **2.5 (0.6 , 5.4)** | **3.9 (1 , 8.3)** | **288 (77 , 621)** | **4.9 (1.3 , 10.2)** | **6.9 (1.8 , 14.7)** | **73.9 (46.3 , 114.9)** |
| **Azerbaijan** | **108 (26 , 234)** | **1.5 (0.4 , 3.3)** | **2.2 (0.5 , 4.7)** | **411 (108 , 885)** | **3.5 (0.9 , 7.1)** | **4.7 (1.2 , 10)** | **115.2 (74.5 , 179.3)** |
| **Georgia** | **185 (46 , 398)** | **2.3 (0.6 , 4.8)** | **3 (0.7 , 6.4)** | **433 (114 , 915)** | **5.2 (1.4 , 10.6)** | **7.2 (1.9 , 15.3)** | **141.8 (101.2 , 203.4)** |
| **Kazakhstan** | **479 (121 , 1035)** | **2.1 (0.5 , 4.5)** | **3.9 (1 , 8.3)** | **907 (244 , 1894)** | **4.3 (1.2 , 8.8)** | **5.5 (1.5 , 11.4)** | **42.5 (20.9 , 74.6)** |
| **Kyrgyzstan** | **57 (14 , 122)** | **1.4 (0.3 , 2.9)** | **1.9 (0.5 , 4.1)** | **82 (21 , 178)** | **2 (0.5 , 4.1)** | **1.9 (0.5 , 4.2)** | **2.8 (-11.5 , 22.1)** |
| **Mongolia** | **17 (4 , 38)** | **0.6 (0.1 , 1.3)** | **1.7 (0.4 , 4)** | **52 (13 , 117)** | **0.9 (0.2 , 1.9)** | **2.8 (0.7 , 6.2)** | **63.1 (28.7 , 114.2)** |
| **Tajikistan** | **44 (11 , 95)** | **1.2 (0.3 , 2.6)** | **1.6 (0.4 , 3.5)** | **141 (37 , 305)** | **2.6 (0.7 , 5.3)** | **3.4 (0.9 , 7.3)** | **114.4 (72.4 , 183.3)** |
| **Turkmenistan** | **26 (6 , 55)** | **1 (0.2 , 2.1)** | **1.4 (0.3 , 2.9)** | **79 (21 , 172)** | **2.2 (0.6 , 4.5)** | **2.1 (0.6 , 4.6)** | **56.6 (27.2 , 96.5)** |
| **Uzbekistan** | **134 (33 , 288)** | **1 (0.3 , 2.2)** | **1.2 (0.3 , 2.6)** | **608 (158 , 1292)** | **2.7 (0.7 , 5.6)** | **3.5 (0.9 , 7.4)** | **190.1 (139.7 , 269.6)** |
| **Central Latin America** | **3125 (886 , 6145)** | **3.2 (0.9 , 6.3)** | **4.2 (1.2 , 8.2)** | **10949 (3168 , 22280)** | **4.7 (1.4 , 9.2)** | **4.8 (1.4 , 9.7)** | **14.1 (-0.7 , 32)** |
| **Colombia** | **663 (181 , 1341)** | **2.8 (0.8 , 5.7)** | **4.3 (1.2 , 8.6)** | **2086 (573 , 4631)** | **4.2 (1.2 , 8.4)** | **3.9 (1.1 , 8.7)** | **-8.2 (-29.5 , 16.4)** |
| **Costa Rica** | **54 (15 , 109)** | **2.3 (0.6 , 4.6)** | **3.3 (0.9 , 6.7)** | **268 (75 , 590)** | **4.4 (1.3 , 8.8)** | **5.3 (1.5 , 11.7)** | **59.4 (24.9 , 105.6)** |
| **El Salvador** | **40 (11 , 83)** | **1.5 (0.4 , 3.2)** | **1.4 (0.4 , 3)** | **230 (64 , 511)** | **3.9 (1.1 , 7.8)** | **3.8 (1.1 , 8.5)** | **167.7 (101 , 256.9)** |
| **Guatemala** | **53 (14 , 111)** | **1.3 (0.4 , 2.7)** | **1.8 (0.5 , 3.6)** | **418 (121 , 902)** | **3.3 (1 , 6.4)** | **4 (1.2 , 8.7)** | **129.7 (81.5 , 205.9)** |
| **Honduras** | **48 (13 , 102)** | **2.2 (0.6 , 4.4)** | **2.5 (0.7 , 5.4)** | **331 (89 , 739)** | **4.3 (1.2 , 9.1)** | **6 (1.6 , 13.1)** | **133.7 (72.9 , 214.1)** |
| **Mexico** | **1892 (552 , 3661)** | **4 (1.2 , 7.8)** | **5 (1.5 , 9.6)** | **5652 (1612 , 11411)** | **5.3 (1.6 , 10.3)** | **5 (1.4 , 10.1)** | **1.1 (-13.5 , 16.6)** |
| **Nicaragua** | **28 (8 , 59)** | **1.9 (0.5 , 4)** | **2.1 (0.6 , 4.4)** | **173 (49 , 355)** | **3.9 (1.1 , 7.8)** | **4.4 (1.3 , 9)** | **110 (66.8 , 168.9)** |
| **Panama** | **43 (12 , 88)** | **2.7 (0.7 , 5.4)** | **3 (0.8 , 6.2)** | **186 (51 , 404)** | **4.7 (1.3 , 9.2)** | **4.5 (1.2 , 9.8)** | **47.6 (15.3 , 92.7)** |
| **Venezuela (Bolivarian Republic of)** | **304 (81 , 626)** | **2.4 (0.6 , 5)** | **3.5 (0.9 , 7.1)** | **1606 (442 , 3600)** | **4.6 (1.3 , 9.2)** | **5.7 (1.6 , 12.8)** | **64.8 (25.4 , 117)** |
| **Andean Latin America** | **317 (83 , 669)** | **1.2 (0.3 , 2.4)** | **1.7 (0.5 , 3.6)** | **1694 (462 , 3598)** | **2.6 (0.7 , 5.3)** | **3.1 (0.9 , 6.7)** | **82.6 (51.6 , 126)** |
| **Bolivia (Plurinational State of)** | **66 (17 , 149)** | **1.2 (0.3 , 2.5)** | **2.3 (0.6 , 5.2)** | **346 (88 , 783)** | **2.5 (0.7 , 5.1)** | **4.3 (1.1 , 9.8)** | **83.9 (46.1 , 135.6)** |
| **Ecuador** | **78 (21 , 162)** | **1.3 (0.3 , 2.6)** | **1.7 (0.4 , 3.4)** | **574 (157 , 1205)** | **3.3 (0.9 , 6.5)** | **4.1 (1.1 , 8.6)** | **147.4 (98.6 , 220.9)** |
| **Peru** | **172 (43 , 369)** | **1.1 (0.3 , 2.3)** | **1.6 (0.4 , 3.4)** | **774 (205 , 1798)** | **2.3 (0.6 , 4.8)** | **2.4 (0.6 , 5.7)** | **53.2 (15.2 , 109.6)** |
| **Caribbean** | **1192 (324 , 2420)** | **3.4 (0.9 , 6.8)** | **4.8 (1.3 , 9.7)** | **3546 (1015 , 7217)** | **5.1 (1.5 , 10)** | **6.9 (2 , 14)** | **43.5 (25 , 69.3)** |
| **Antigua and Barbuda** | **2 (1 , 4)** | **2.8 (0.8 , 5.7)** | **3.8 (1.1 , 7.8)** | **7 (2 , 14)** | **5 (1.5 , 10)** | **7.4 (2.2 , 15.3)** | **93.4 (68.1 , 133.1)** |
| **Barbados** | **14 (4 , 29)** | **3.2 (0.9 , 6.3)** | **4.7 (1.3 , 9.5)** | **42 (12 , 89)** | **5.1 (1.5 , 10.1)** | **8.4 (2.4 , 17.7)** | **79.4 (49.7 , 118.3)** |
| **Belize** | **2 (0 , 3)** | **1.7 (0.5 , 3.6)** | **1.8 (0.5 , 3.8)** | **10 (3 , 21)** | **3.4 (0.9 , 6.8)** | **4 (1.1 , 8.2)** | **120.2 (87 , 167.5)** |
| **Bermuda** | **4 (1 , 8)** | **3.3 (0.9 , 6.8)** | **6.9 (1.8 , 14.3)** | **9 (3 , 20)** | **5.1 (1.4 , 10.4)** | **6.6 (1.8 , 14.3)** | **-3 (-17.4 , 21.7)** |
| **Bahamas** | **7 (2 , 15)** | **2.7 (0.7 , 5.6)** | **5 (1.3 , 10.2)** | **26 (7 , 55)** | **4.5 (1.3 , 9.1)** | **7.2 (2.1 , 14.9)** | **44.6 (19 , 78.8)** |
| **Cuba** | **629 (167 , 1291)** | **4.2 (1.1 , 8.7)** | **6.2 (1.6 , 12.7)** | **1660 (454 , 3497)** | **6 (1.7 , 12.1)** | **8.5 (2.3 , 17.9)** | **37.6 (12.6 , 72.3)** |
| **Dominica** | **4 (1 , 8)** | **2.6 (0.7 , 5.3)** | **5 (1.4 , 10.2)** | **8 (2 , 17)** | **4.6 (1.4 , 8.8)** | **9.1 (2.6 , 18.8)** | **83.7 (51.3 , 135.5)** |
| **Dominican Republic** | **40 (10 , 85)** | **1.2 (0.3 , 2.5)** | **1.2 (0.3 , 2.6)** | **269 (69 , 612)** | **2.5 (0.7 , 5.2)** | **3 (0.8 , 6.9)** | **151.4 (83.4 , 241.1)** |
| **Grenada** | **4 (1 , 7)** | **2.8 (0.8 , 5.6)** | **4.8 (1.3 , 9.8)** | **10 (3 , 19)** | **5.3 (1.6 , 10.4)** | **9.4 (2.8 , 18.5)** | **95.9 (71.7 , 134.1)** |
| **Guyana** | **14 (4 , 30)** | **2.8 (0.8 , 5.7)** | **4.2 (1.2 , 8.6)** | **42 (12 , 90)** | **5.4 (1.6 , 10.4)** | **7.2 (2.1 , 15.1)** | **71.1 (33.2 , 123.8)** |
| **Haiti** | **110 (29 , 239)** | **1.9 (0.5 , 4)** | **3.7 (1 , 8)** | **359 (97 , 814)** | **3.4 (1 , 7)** | **5.7 (1.5 , 12.8)** | **52.3 (16.6 , 104.3)** |
| **Jamaica** | **63 (17 , 128)** | **3.1 (0.8 , 6.2)** | **3.5 (0.9 , 7)** | **237 (67 , 500)** | **5.5 (1.6 , 10.9)** | **7.9 (2.2 , 16.8)** | **127.5 (83.7 , 191.4)** |
| **Puerto Rico** | **187 (53 , 373)** | **4.1 (1.2 , 8.2)** | **5.2 (1.5 , 10.3)** | **514 (153 , 1112)** | **7.4 (2.2 , 14.2)** | **6.7 (2 , 14.7)** | **30.2 (1.8 , 67.7)** |
| **Saint Kitts and Nevis** | **3 (1 , 5)** | **3.5 (1 , 6.8)** | **6.9 (1.9 , 13.8)** | **5 (1 , 10)** | **4.9 (1.4 , 9.7)** | **8.4 (2.5 , 17.3)** | **21.9 (3.3 , 48.6)** |
| **Saint Lucia** | **5 (2 , 11)** | **3.7 (1.1 , 7.2)** | **6.6 (1.9 , 12.7)** | **17 (5 , 34)** | **5.6 (1.7 , 10.7)** | **8.1 (2.5 , 16.3)** | **23 (3.8 , 48.7)** |
| **Saint Vincent and the Grenadines** | **3 (1 , 7)** | **3.1 (0.9 , 6.2)** | **5 (1.4 , 9.9)** | **10 (3 , 20)** | **4.8 (1.4 , 9.2)** | **7.6 (2.3 , 15.3)** | **51.9 (31.7 , 81.2)** |
| **Suriname** | **9 (3 , 19)** | **3.1 (0.9 , 6.3)** | **3.8 (1.1 , 7.6)** | **46 (13 , 94)** | **6.4 (1.9 , 12.3)** | **8 (2.3 , 16.2)** | **110 (76.6 , 161)** |
| **Trinidad and Tobago** | **49 (14 , 96)** | **4.3 (1.2 , 8.4)** | **6.2 (1.8 , 12.2)** | **136 (39 , 296)** | **6.7 (2 , 12.9)** | **7.4 (2.1 , 16)** | **19.1 (-9.4 , 60.4)** |
| **United States Virgin Islands** | **4 (1 , 9)** | **3.2 (0.9 , 6.5)** | **5.3 (1.5 , 11.4)** | **20 (6 , 41)** | **6.1 (1.8 , 12)** | **10.9 (3.1 , 22)** | **104.1 (69.2 , 154.9)** |
| **Tropical Latin America** | **3052 (828 , 6187)** | **2.6 (0.7 , 5.2)** | **3.7 (1 , 7.6)** | **9761 (2712 , 19987)** | **3.6 (1 , 7.2)** | **4.2 (1.2 , 8.5)** | **11.5 (5 , 19.9)** |
| **Brazil** | **3018 (820 , 6118)** | **2.6 (0.7 , 5.2)** | **3.8 (1 , 7.7)** | **9534 (2646 , 19518)** | **3.6 (1 , 7.2)** | **4.2 (1.2 , 8.5)** | **9.7 (3.4 , 18.1)** |
| **Paraguay** | **34 (9 , 72)** | **1.7 (0.4 , 3.5)** | **1.6 (0.4 , 3.5)** | **227 (59 , 508)** | **3.7 (1 , 7.5)** | **4.3 (1.1 , 9.6)** | **163 (96.7 , 248.5)** |
| **East Asia** | **25734 (6560 , 55611)** | **1.7 (0.4 , 3.7)** | **3.2 (0.8 , 7)** | **94519 (24177 , 204701)** | **3.4 (0.9 , 7.1)** | **4.7 (1.2 , 10.3)** | **45.8 (21.2 , 76.6)** |
| **China** | **24887 (6343 , 53939)** | **1.7 (0.4 , 3.7)** | **3.3 (0.8 , 7)** | **90655 (23078 , 197161)** | **3.3 (0.9 , 7)** | **4.7 (1.2 , 10.2)** | **44.2 (18.4 , 76.1)** |
| **Democratic People's Republic of Korea** | **327 (82 , 748)** | **1.4 (0.4 , 3)** | **2.2 (0.6 , 5)** | **1106 (281 , 2435)** | **2.7 (0.7 , 5.8)** | **3.5 (0.9 , 7.7)** | **57.2 (25.6 , 96.2)** |
| **Taiwan (Province of China)** | **520 (137 , 1085)** | **2.5 (0.7 , 5.2)** | **3.5 (0.9 , 7.4)** | **2758 (732 , 5951)** | **4.8 (1.3 , 9.8)** | **6.9 (1.8 , 14.9)** | **94.8 (54.5 , 153.2)** |
| **Southeast Asia** | **5797 (1472 , 12380)** | **2 (0.5 , 4.2)** | **2.6 (0.7 , 5.6)** | **26110 (7020 , 55023)** | **3.9 (1.1 , 8)** | **4.8 (1.3 , 10.1)** | **85.4 (58.3 , 119.3)** |
| **Cambodia** | **76 (18 , 173)** | **1.2 (0.3 , 2.7)** | **2 (0.5 , 4.4)** | **588 (156 , 1251)** | **4.1 (1.1 , 8.3)** | **5.6 (1.5 , 11.7)** | **185.2 (123.6 , 271.6)** |
| **Indonesia** | **1558 (392 , 3381)** | **1.6 (0.4 , 3.5)** | **1.8 (0.5 , 4)** | **8032 (2038 , 17890)** | **3.5 (0.9 , 7.3)** | **4.3 (1.1 , 9.5)** | **133.6 (88.5 , 183.8)** |
| **Lao People's Democratic Republic** | **63 (15 , 144)** | **2.1 (0.5 , 4.5)** | **3.4 (0.8 , 7.6)** | **223 (59 , 490)** | **4.5 (1.2 , 9.2)** | **6 (1.6 , 13)** | **77.1 (36.6 , 134.9)** |
| **Malaysia** | **332 (88 , 687)** | **3 (0.8 , 6.1)** | **4.2 (1.1 , 8.6)** | **1701 (461 , 3665)** | **5.4 (1.6 , 10.9)** | **7.3 (2 , 15.4)** | **75.3 (34.3 , 131.5)** |
| **Maldives** | **2 (0 , 4)** | **1.8 (0.5 , 3.9)** | **2.6 (0.7 , 5.8)** | **8 (2 , 18)** | **4 (1.1 , 7.9)** | **3.5 (1 , 7.2)** | **30.6 (3.3 , 75.3)** |
| **Mauritius** | **24 (6 , 49)** | **3.6 (1 , 7.3)** | **3.6 (1 , 7.2)** | **128 (39 , 264)** | **8.5 (2.6 , 16.1)** | **7.6 (2.3 , 15.5)** | **108.8 (68.7 , 173)** |
| **Myanmar** | **774 (188 , 1807)** | **2.3 (0.6 , 5)** | **3.6 (0.9 , 8.3)** | **2304 (618 , 5071)** | **4.4 (1.2 , 8.9)** | **5.6 (1.5 , 12.2)** | **54 (18.2 , 117.9)** |
| **Philippines** | **927 (239 , 1941)** | **2.5 (0.6 , 5.1)** | **3.8 (1 , 7.9)** | **2871 (758 , 6284)** | **3.5 (1 , 7.2)** | **4.1 (1.1 , 9)** | **9.6 (-10.7 , 37.7)** |
| **Sri Lanka** | **164 (45 , 335)** | **1.9 (0.5 , 4)** | **1.8 (0.5 , 3.7)** | **1091 (323 , 2385)** | **5.8 (1.8 , 11.1)** | **4.5 (1.4 , 9.9)** | **152.3 (89.4 , 242.6)** |
| **Seychelles** | **3 (1 , 5)** | **2.9 (0.8 , 5.9)** | **4.5 (1.2 , 9.4)** | **11 (3 , 22)** | **6.3 (1.9 , 12)** | **11.6 (3.6 , 22.6)** | **156.8 (119.7 , 219.7)** |
| **Thailand** | **991 (255 , 2120)** | **2 (0.5 , 4.3)** | **3.2 (0.8 , 6.9)** | **4390 (1140 , 9981)** | **3.8 (1 , 7.8)** | **4.4 (1.2 , 10)** | **36.7 (3.2 , 86.9)** |
| **Timor-Leste** | **4 (1 , 8)** | **1.2 (0.3 , 2.8)** | **1.7 (0.4 , 3.8)** | **32 (8 , 71)** | **3.9 (1.1 , 8.1)** | **4.4 (1.1 , 9.6)** | **157 (84.4 , 255.5)** |
| **Viet Nam** | **871 (215 , 1914)** | **1.9 (0.5 , 4.2)** | **2.3 (0.6 , 5)** | **4694 (1220 , 10093)** | **4.2 (1.1 , 8.5)** | **5.7 (1.5 , 12.1)** | **147 (93.5 , 223.8)** |
| **Oceania** | **111 (28 , 245)** | **3.2 (0.9 , 6.8)** | **4.2 (1.1 , 9.2)** | **486 (131 , 1052)** | **5.5 (1.6 , 11.2)** | **7.4 (2.1 , 15.9)** | **77.4 (47.4 , 121)** |
| **American Samoa** | **2 (1 , 4)** | **6.3 (1.8 , 12.4)** | **10.1 (2.9 , 20.3)** | **7 (2 , 13)** | **10.1 (3.2 , 18.6)** | **15.5 (4.9 , 29.9)** | **52.7 (26.3 , 91.5)** |
| **Cook Islands** | **1 (0 , 2)** | **4.8 (1.3 , 9.8)** | **7.9 (2 , 16.4)** | **3 (1 , 5)** | **8.3 (2.4 , 15.9)** | **10.8 (3 , 22.1)** | **36.9 (10.5 , 76.8)** |
| **Micronesia (Federated States of)** | **2 (1 , 5)** | **2.8 (0.7 , 5.8)** | **4.8 (1.2 , 10.7)** | **7 (2 , 17)** | **6.6 (1.9 , 13.4)** | **11.7 (3.2 , 26.5)** | **141.3 (71.7 , 255.6)** |
| **Fiji** | **16 (5 , 34)** | **3.9 (1.1 , 8)** | **5.2 (1.5 , 10.9)** | **64 (19 , 133)** | **7.9 (2.5 , 15)** | **9.6 (2.9 , 19.7)** | **83.5 (40.2 , 160)** |
| **Guam** | **4 (1 , 8)** | **4 (1 , 8.4)** | **6 (1.6 , 13)** | **13 (4 , 27)** | **6.1 (1.7 , 12.4)** | **6.8 (1.9 , 14.5)** | **13.3 (-8.8 , 44.7)** |
| **Kiribati** | **2 (0 , 4)** | **2.2 (0.6 , 4.7)** | **4.9 (1.3 , 10.4)** | **5 (1 , 11)** | **4.1 (1.1 , 8.3)** | **8.3 (2.2 , 17.8)** | **70.5 (30 , 130)** |
| **Marshall Islands** | **1 (0 , 3)** | **4.6 (1.3 , 9.8)** | **8.1 (2.2 , 17.8)** | **5 (1 , 10)** | **8.4 (2.6 , 16.6)** | **14.6 (4.1 , 32)** | **80.6 (39.9 , 138)** |
| **Nauru** | **0 (0 , 0)** | **3 (0.8 , 6.3)** | **7.1 (1.8 , 16.2)** | **0 (0 , 1)** | **5.3 (1.5 , 11.1)** | **12 (3.2 , 26.3)** | **69.6 (34.7 , 123.2)** |
| **Niue** | **0 (0 , 0)** | **5.7 (1.6 , 11.2)** | **7.6 (2.2 , 15.6)** | **0 (0 , 1)** | **9.9 (3 , 19)** | **13.5 (4 , 27.4)** | **78.1 (38.4 , 134.6)** |
| **Northern Mariana Islands** | **1 (0 , 2)** | **3.6 (0.9 , 7.6)** | **8.8 (2.2 , 19.1)** | **6 (2 , 11)** | **7 (1.9 , 14)** | **12.5 (3.4 , 25.3)** | **42.3 (20.2 , 75.1)** |
| **Palau** | **1 (0 , 2)** | **4.2 (1.1 , 8.5)** | **8.5 (2.3 , 18.6)** | **3 (1 , 6)** | **7.7 (2.3 , 15)** | **14.6 (4.2 , 29.9)** | **70.9 (27.5 , 132.4)** |
| **Papua New Guinea** | **62 (15 , 145)** | **3.1 (0.8 , 6.7)** | **3.6 (0.9 , 8.4)** | **291 (75 , 655)** | **5.1 (1.4 , 10.6)** | **6.5 (1.7 , 14.1)** | **77.7 (38.6 , 133.6)** |
| **Samoa** | **3 (1 , 7)** | **3.2 (0.9 , 6.4)** | **3.9 (1.1 , 8.3)** | **9 (3 , 19)** | **5.7 (1.7 , 11.4)** | **6.4 (1.9 , 13.6)** | **62.8 (29.9 , 113)** |
| **Solomon Islands** | **4 (1 , 11)** | **2 (0.5 , 4.4)** | **3.6 (0.8 , 8.8)** | **32 (8 , 72)** | **4.9 (1.3 , 10.1)** | **10.4 (2.6 , 23.2)** | **186.5 (114.5 , 309.6)** |
| **Tokelau** | **0 (0 , 0)** | **3.8 (1 , 7.7)** | **4.7 (1.2 , 10)** | **0 (0 , 0)** | **6.9 (2 , 13.5)** | **8.7 (2.4 , 18.7)** | **82.9 (43.9 , 141.1)** |
| **Tonga** | **3 (1 , 6)** | **3.3 (0.9 , 6.9)** | **5.4 (1.4 , 11.6)** | **7 (2 , 15)** | **5.4 (1.5 , 10.8)** | **8.9 (2.4 , 18.8)** | **63.4 (27.5 , 114.5)** |
| **Tuvalu** | **0 (0 , 1)** | **3.2 (0.9 , 6.7)** | **5 (1.3 , 10.9)** | **1 (0 , 2)** | **6.6 (2 , 13.1)** | **9.4 (2.6 , 20.3)** | **87.5 (43.7 , 156)** |
| **Vanuatu** | **2 (0 , 5)** | **2.6 (0.7 , 5.8)** | **3.5 (0.8 , 8.3)** | **12 (3 , 26)** | **5.1 (1.4 , 10.5)** | **7.3 (1.9 , 16.1)** | **107 (53.9 , 210)** |
| **North Africa and Middle East** | **4023 (1032 , 8604)** | **2.2 (0.6 , 4.7)** | **2.6 (0.7 , 5.6)** | **19755 (5517 , 40246)** | **4.7 (1.3 , 9.3)** | **5.1 (1.4 , 10.3)** | **93.9 (68.3 , 133.3)** |
| **Afghanistan** | **175 (42 , 410)** | **1.5 (0.4 , 3.3)** | **2.6 (0.7 , 6.1)** | **510 (140 , 1146)** | **2.4 (0.7 , 5)** | **4.8 (1.4 , 10.4)** | **82.2 (39.9 , 137.8)** |
| **Algeria** | **239 (62 , 526)** | **2.3 (0.6 , 4.7)** | **2.4 (0.6 , 5.2)** | **1236 (353 , 2587)** | **5.2 (1.5 , 10.3)** | **4.2 (1.2 , 8.7)** | **74.8 (36.2 , 134.3)** |
| **Bahrain** | **17 (5 , 34)** | **6.5 (1.8 , 12.8)** | **13.3 (3.7 , 26.5)** | **84 (25 , 166)** | **10.3 (3.3 , 18.9)** | **13.7 (4.3 , 26.5)** | **3.2 (-19.8 , 42.5)** |
| **Egypt** | **312 (84 , 651)** | **1.3 (0.4 , 2.8)** | **1.2 (0.3 , 2.4)** | **2116 (568 , 4855)** | **3.7 (1 , 7.5)** | **3.6 (1 , 8.1)** | **204.4 (122.5 , 323.9)** |
| **Iran (Islamic Republic of)** | **389 (100 , 844)** | **1.5 (0.4 , 3.1)** | **1.8 (0.5 , 3.9)** | **2689 (777 , 5382)** | **4 (1.2 , 8)** | **4.1 (1.2 , 8.1)** | **122.7 (92.7 , 174)** |
| **Iraq** | **267 (69 , 590)** | **3.4 (0.9 , 7.1)** | **3.7 (1 , 8.1)** | **1434 (410 , 3032)** | **5.9 (1.7 , 11.6)** | **7 (2 , 14.5)** | **91.4 (45 , 155)** |
| **Jordan** | **45 (12 , 95)** | **3.3 (0.9 , 6.6)** | **4 (1.1 , 8.3)** | **335 (93 , 696)** | **5.9 (1.7 , 11.6)** | **6.1 (1.7 , 12.6)** | **52.6 (23.9 , 95.8)** |
| **Kuwait** | **20 (6 , 40)** | **4 (1.1 , 8)** | **4.3 (1.2 , 8.4)** | **123 (36 , 248)** | **7.4 (2.2 , 14.4)** | **6.2 (1.9 , 12.4)** | **45.8 (23 , 82.4)** |
| **Lebanon** | **108 (28 , 232)** | **3.7 (1 , 7.6)** | **5.3 (1.4 , 11.3)** | **564 (160 , 1168)** | **7.5 (2.2 , 14.7)** | **10.9 (3.1 , 22.4)** | **104 (65.1 , 189.3)** |
| **Libya** | **70 (18 , 156)** | **3.5 (0.9 , 7.3)** | **4.2 (1.1 , 9.3)** | **370 (102 , 777)** | **6.8 (2 , 13.5)** | **8.1 (2.3 , 16.8)** | **93.5 (48.4 , 172.1)** |
| **Morocco** | **266 (66 , 583)** | **2.4 (0.6 , 5.1)** | **2.1 (0.5 , 4.5)** | **1504 (398 , 3276)** | **5.4 (1.5 , 11.1)** | **5 (1.3 , 10.9)** | **143.5 (87.7 , 221.9)** |
| **Palestine** | **37 (9 , 83)** | **3.3 (0.9 , 7)** | **4.7 (1.2 , 10.3)** | **201 (57 , 407)** | **6.9 (2 , 13.5)** | **10 (2.9 , 20)** | **113.6 (64.6 , 198.5)** |
| **Oman** | **12 (3 , 27)** | **2.1 (0.5 , 4.4)** | **2.2 (0.6 , 4.9)** | **62 (18 , 127)** | **4.5 (1.3 , 9)** | **5.2 (1.5 , 10.4)** | **136.4 (82.7 , 226.9)** |
| **Qatar** | **6 (2 , 12)** | **4.4 (1.3 , 8.8)** | **9 (2.6 , 18.6)** | **73 (22 , 148)** | **8.8 (2.8 , 16.4)** | **15.8 (5.1 , 30.3)** | **74.7 (32.4 , 147.6)** |
| **Saudi Arabia** | **118 (31 , 264)** | **2.8 (0.8 , 5.8)** | **2.4 (0.6 , 5.3)** | **680 (191 , 1416)** | **5.2 (1.5 , 10.2)** | **4.9 (1.4 , 10)** | **103.6 (56.5 , 184.5)** |
| **Sudan** | **129 (32 , 314)** | **1.5 (0.4 , 3.4)** | **1.5 (0.4 , 3.7)** | **586 (165 , 1318)** | **3.4 (1 , 7.1)** | **3.6 (1 , 7.9)** | **132.3 (80.4 , 223.4)** |
| **Syrian Arab Republic** | **84 (22 , 188)** | **1.8 (0.5 , 3.9)** | **1.8 (0.5 , 4)** | **381 (101 , 828)** | **4.3 (1.2 , 8.6)** | **3.5 (1 , 7.4)** | **92.9 (40.2 , 172)** |
| **Tunisia** | **164 (39 , 355)** | **4 (1 , 8.5)** | **3.6 (0.9 , 7.9)** | **785 (206 , 1769)** | **7.8 (2.1 , 15.4)** | **6.5 (1.7 , 14.7)** | **80.2 (29.4 , 161.7)** |
| **Turkey** | **1488 (356 , 3300)** | **2.7 (0.7 , 5.9)** | **4.4 (1.1 , 9.8)** | **5434 (1367 , 11696)** | **5.2 (1.4 , 10.6)** | **6.4 (1.6 , 13.7)** | **43.1 (9.7 , 91.7)** |
| **United Arab Emirates** | **21 (6 , 44)** | **3.3 (0.9 , 6.6)** | **8.8 (2.4 , 18.2)** | **280 (77 , 588)** | **5.2 (1.5 , 10.4)** | **12.8 (3.8 , 25.7)** | **46.3 (13.2 , 99.4)** |
| **Yemen** | **53 (13 , 125)** | **1.2 (0.3 , 2.6)** | **1.2 (0.3 , 2.9)** | **286 (74 , 644)** | **2.3 (0.6 , 4.8)** | **2.5 (0.7 , 5.4)** | **97.7 (55.9 , 169.9)** |
| **South Asia** | **7297 (1868 , 15669)** | **1.4 (0.4 , 3)** | **1.5 (0.4 , 3.2)** | **38990 (10922 , 80737)** | **3.1 (0.9 , 6.3)** | **3 (0.8 , 6.2)** | **95.6 (66.1 , 137.3)** |
| **Bangladesh** | **531 (121 , 1202)** | **1 (0.2 , 2.3)** | **1.2 (0.3 , 2.9)** | **2240 (545 , 5302)** | **2.1 (0.6 , 4.4)** | **1.8 (0.4 , 4.3)** | **45.9 (10.7 , 91.5)** |
| **Bhutan** | **2 (1 , 5)** | **1 (0.2 , 2.3)** | **1 (0.2 , 2.3)** | **12 (3 , 27)** | **2.6 (0.7 , 5.4)** | **2.4 (0.6 , 5.2)** | **134.1 (76.4 , 217.5)** |
| **India** | **5294 (1380 , 11410)** | **1.4 (0.4 , 3)** | **1.4 (0.4 , 3)** | **30220 (8320 , 62897)** | **3.2 (0.9 , 6.5)** | **2.9 (0.8 , 5.9)** | **102.1 (69.6 , 148.4)** |
| **Nepal** | **83 (19 , 201)** | **0.9 (0.2 , 2)** | **1 (0.2 , 2.4)** | **514 (128 , 1128)** | **2.4 (0.7 , 5)** | **2.5 (0.6 , 5.4)** | **149 (78.4 , 248.2)** |
| **Pakistan** | **1386 (348 , 3039)** | **1.9 (0.5 , 4.1)** | **2.6 (0.6 , 5.6)** | **6003 (1555 , 12977)** | **3.3 (0.9 , 6.9)** | **6 (1.6 , 12.8)** | **131.7 (81.6 , 215.5)** |
| **Southern Sub-Saharan Africa** | **853 (220 , 1861)** | **2.3 (0.6 , 4.9)** | **3.6 (0.9 , 7.8)** | **2886 (811 , 5789)** | **3.9 (1.1 , 7.8)** | **5.8 (1.6 , 11.5)** | **61.7 (38.2 , 88.7)** |
| **Botswana** | **15 (4 , 34)** | **2.1 (0.5 , 4.4)** | **3.2 (0.8 , 7)** | **80 (21 , 173)** | **3.9 (1.1 , 7.9)** | **7.1 (1.9 , 15.2)** | **122 (62.1 , 207.6)** |
| **Lesotho** | **17 (4 , 38)** | **1.6 (0.4 , 3.5)** | **2 (0.5 , 4.4)** | **59 (15 , 135)** | **2.7 (0.7 , 5.6)** | **5.3 (1.4 , 11.9)** | **167.1 (80.7 , 279)** |
| **Namibia** | **12 (3 , 25)** | **1.9 (0.5 , 4)** | **1.8 (0.5 , 3.9)** | **45 (12 , 99)** | **2.8 (0.8 , 5.8)** | **3.5 (0.9 , 7.6)** | **98.3 (53.7 , 161.2)** |
| **South Africa** | **685 (173 , 1541)** | **2.5 (0.7 , 5.2)** | **3.7 (0.9 , 8.3)** | **2277 (634 , 4600)** | **4.2 (1.2 , 8.3)** | **5.6 (1.6 , 11.3)** | **53.2 (30.4 , 81.2)** |
| **Eswatini** | **8 (2 , 18)** | **2 (0.5 , 4.2)** | **3.4 (0.9 , 7.3)** | **32 (8 , 73)** | **3.3 (0.9 , 6.7)** | **6.5 (1.7 , 14.7)** | **91.8 (40.7 , 165.4)** |
| **Zimbabwe** | **116 (32 , 243)** | **1.9 (0.5 , 4.1)** | **3.5 (1 , 7.3)** | **392 (108 , 835)** | **3.2 (0.9 , 6.4)** | **6.6 (1.8 , 13.9)** | **88.9 (48.4 , 147.1)** |
| **Western Sub-Saharan Africa** | **1060 (280 , 2311)** | **1.3 (0.3 , 2.7)** | **1.4 (0.4 , 3.1)** | **4174 (1131 , 8853)** | **2.1 (0.6 , 4.4)** | **2.7 (0.7 , 5.7)** | **88.1 (60.1 , 122.8)** |
| **Benin** | **30 (8 , 65)** | **1.4 (0.4 , 2.9)** | **1.7 (0.4 , 3.6)** | **121 (33 , 261)** | **2.3 (0.6 , 4.7)** | **3 (0.8 , 6.4)** | **81.2 (46.2 , 131.9)** |
| **Burkina Faso** | **60 (15 , 133)** | **1.4 (0.4 , 3)** | **1.7 (0.4 , 3.7)** | **204 (54 , 439)** | **2 (0.6 , 4.2)** | **2.7 (0.7 , 5.8)** | **62.3 (33.3 , 101.4)** |
| **Cameroon** | **66 (17 , 146)** | **1.4 (0.4 , 2.9)** | **1.8 (0.5 , 4)** | **365 (93 , 813)** | **2.6 (0.7 , 5.3)** | **3.8 (1 , 8.4)** | **106.5 (60.1 , 171.2)** |
| **Cabo Verde** | **3 (1 , 7)** | **1.3 (0.3 , 2.8)** | **1.4 (0.4 , 3)** | **23 (6 , 48)** | **3.2 (0.9 , 6.6)** | **5.8 (1.6 , 12.3)** | **310.7 (219.2 , 426.1)** |
| **Chad** | **33 (9 , 73)** | **1.3 (0.3 , 2.7)** | **1.3 (0.3 , 2.8)** | **119 (30 , 263)** | **1.9 (0.5 , 4.1)** | **2.6 (0.7 , 5.6)** | **98.7 (60 , 152.3)** |
| **CÃ´te d'Ivoire** | **56 (14 , 122)** | **1.2 (0.3 , 2.6)** | **1.9 (0.5 , 4.2)** | **272 (72 , 594)** | **2.4 (0.7 , 4.9)** | **3.4 (0.9 , 7.2)** | **74.5 (41.9 , 120.1)** |
| **Gambia** | **3 (1 , 7)** | **0.9 (0.2 , 2)** | **1 (0.3 , 2.3)** | **20 (5 , 43)** | **1.9 (0.5 , 3.9)** | **2.4 (0.6 , 5.1)** | **133.4 (74.6 , 212.2)** |
| **Ghana** | **101 (27 , 224)** | **1.4 (0.4 , 2.9)** | **2 (0.5 , 4.4)** | **561 (152 , 1200)** | **3 (0.8 , 6.3)** | **4.1 (1.1 , 8.8)** | **109.1 (60.2 , 169.1)** |
| **Guinea** | **44 (11 , 95)** | **0.9 (0.2 , 2)** | **1.5 (0.4 , 3.2)** | **138 (36 , 302)** | **1.6 (0.4 , 3.4)** | **2.9 (0.8 , 6.2)** | **92.6 (50.9 , 147.3)** |
| **Guinea-Bissau** | **7 (2 , 17)** | **1.2 (0.3 , 2.6)** | **2 (0.5 , 4.7)** | **20 (5 , 45)** | **2 (0.5 , 4.3)** | **3.4 (0.9 , 7.5)** | **65.7 (27.5 , 120)** |
| **Liberia** | **21 (6 , 47)** | **1.8 (0.5 , 3.9)** | **2.1 (0.6 , 4.7)** | **54 (14 , 120)** | **2.7 (0.7 , 5.5)** | **3.3 (0.9 , 7.3)** | **55.1 (18.9 , 106.4)** |
| **Mali** | **55 (14 , 120)** | **1.2 (0.3 , 2.5)** | **1.6 (0.4 , 3.4)** | **202 (55 , 437)** | **2.2 (0.6 , 4.5)** | **2.8 (0.8 , 6)** | **75 (39.5 , 118.1)** |
| **Mauritania** | **14 (3 , 30)** | **1.2 (0.3 , 2.4)** | **1.5 (0.4 , 3.3)** | **43 (11 , 94)** | **2.1 (0.6 , 4.3)** | **2.4 (0.6 , 5.1)** | **56 (23 , 102.3)** |
| **Niger** | **14 (3 , 32)** | **0.6 (0.1 , 1.3)** | **0.7 (0.2 , 1.5)** | **89 (22 , 202)** | **1.3 (0.3 , 2.7)** | **1.4 (0.4 , 3.3)** | **114.6 (71.9 , 168.9)** |
| **Nigeria** | **454 (115 , 1025)** | **1.3 (0.3 , 2.8)** | **1.2 (0.3 , 2.7)** | **1544 (392 , 3374)** | **1.9 (0.5 , 4.1)** | **2.2 (0.6 , 4.8)** | **78.9 (34.5 , 129.5)** |
| **Sao Tome and Principe** | **1 (0 , 3)** | **1.8 (0.5 , 3.7)** | **2.3 (0.6 , 5)** | **4 (1 , 9)** | **3 (0.8 , 6.3)** | **5 (1.3 , 10.8)** | **113.9 (71 , 167.3)** |
| **Senegal** | **70 (18 , 150)** | **2.1 (0.6 , 4.4)** | **2.5 (0.7 , 5.4)** | **275 (74 , 597)** | **3.4 (0.9 , 6.9)** | **4.3 (1.2 , 9.1)** | **68 (35.9 , 117.4)** |
| **Sierra Leone** | **15 (4 , 35)** | **0.8 (0.2 , 1.8)** | **0.9 (0.2 , 2.1)** | **55 (14 , 122)** | **1.5 (0.4 , 3.1)** | **1.8 (0.5 , 4)** | **101.1 (53 , 169.1)** |
| **Togo** | **13 (3 , 28)** | **0.9 (0.2 , 2)** | **1.3 (0.3 , 2.8)** | **64 (16 , 141)** | **1.7 (0.4 , 3.5)** | **2.2 (0.6 , 4.9)** | **72.4 (34.9 , 119.3)** |
| **Eastern Sub-Saharan Africa** | **821 (212 , 1797)** | **0.8 (0.2 , 1.8)** | **1.3 (0.3 , 2.9)** | **2682 (707 , 5689)** | **1.4 (0.4 , 2.9)** | **2 (0.5 , 4.2)** | **49.5 (32.8 , 71)** |
| **Burundi** | **32 (8 , 71)** | **0.9 (0.2 , 2)** | **1.5 (0.4 , 3.4)** | **63 (15 , 147)** | **1.2 (0.3 , 2.5)** | **1.7 (0.4 , 3.9)** | **8.7 (-20.8 , 47.5)** |
| **Comoros** | **3 (1 , 6)** | **1 (0.3 , 2.3)** | **1.3 (0.3 , 3)** | **8 (2 , 18)** | **1.6 (0.4 , 3.3)** | **1.9 (0.5 , 4.1)** | **45.6 (11.1 , 106.4)** |
| **Djibouti** | **2 (0 , 4)** | **0.9 (0.2 , 1.9)** | **1.5 (0.4 , 3.5)** | **12 (3 , 29)** | **1.7 (0.4 , 3.5)** | **2.7 (0.7 , 6.1)** | **75.6 (36.4 , 126.5)** |
| **Eritrea** | **9 (2 , 22)** | **0.7 (0.2 , 1.6)** | **1.2 (0.3 , 2.8)** | **52 (12 , 115)** | **1.4 (0.4 , 3.1)** | **2.4 (0.6 , 5.3)** | **101.9 (48.7 , 181.5)** |
| **Ethiopia** | **232 (57 , 530)** | **0.8 (0.2 , 2)** | **1.4 (0.3 , 3.1)** | **494 (121 , 1116)** | **1.2 (0.3 , 2.6)** | **1.4 (0.4 , 3.2)** | **1.7 (-18.2 , 32.2)** |
| **Kenya** | **58 (14 , 134)** | **0.8 (0.2 , 1.9)** | **0.8 (0.2 , 1.9)** | **300 (77 , 666)** | **1.3 (0.3 , 2.7)** | **1.6 (0.4 , 3.6)** | **96.4 (64.8 , 140.4)** |
| **Madagascar** | **50 (13 , 110)** | **0.8 (0.2 , 1.8)** | **1.1 (0.3 , 2.5)** | **138 (35 , 331)** | **1.3 (0.3 , 2.7)** | **1.6 (0.4 , 3.6)** | **36.8 (2.7 , 77)** |
| **Malawi** | **51 (13 , 110)** | **0.8 (0.2 , 1.8)** | **1.6 (0.4 , 3.5)** | **154 (41 , 327)** | **1.5 (0.4 , 3.2)** | **2.5 (0.7 , 5.4)** | **54.4 (26 , 91.2)** |
| **Mozambique** | **59 (15 , 129)** | **1 (0.3 , 2.1)** | **1.2 (0.3 , 2.7)** | **232 (60 , 515)** | **1.7 (0.5 , 3.6)** | **2.6 (0.7 , 5.7)** | **107.8 (55.1 , 177.8)** |
| **Rwanda** | **43 (11 , 95)** | **0.9 (0.2 , 2)** | **1.7 (0.5 , 3.8)** | **108 (27 , 241)** | **1.5 (0.4 , 3.2)** | **2.2 (0.6 , 4.8)** | **27.5 (-0.3 , 68.6)** |
| **Somalia** | **23 (5 , 54)** | **0.7 (0.2 , 1.4)** | **1.1 (0.3 , 2.7)** | **80 (18 , 205)** | **1 (0.2 , 2.2)** | **1.5 (0.3 , 3.8)** | **28.6 (-7.9 , 75.2)** |
| **South Sudan** | **33 (8 , 77)** | **1.1 (0.3 , 2.5)** | **1.6 (0.4 , 3.7)** | **64 (16 , 147)** | **1.6 (0.4 , 3.3)** | **2.1 (0.5 , 4.7)** | **29.3 (-2 , 71.3)** |
| **United Republic of Tanzania** | **97 (25 , 219)** | **0.7 (0.2 , 1.5)** | **1.1 (0.3 , 2.4)** | **439 (113 , 955)** | **1.4 (0.4 , 3)** | **2.1 (0.5 , 4.6)** | **89.3 (58.5 , 126.1)** |
| **Uganda** | **85 (22 , 184)** | **1 (0.3 , 2.2)** | **1.5 (0.4 , 3.3)** | **375 (100 , 806)** | **1.7 (0.5 , 3.6)** | **3.1 (0.8 , 6.7)** | **104.6 (63.8 , 162)** |
| **Zambia** | **45 (12 , 101)** | **1 (0.3 , 2.1)** | **1.9 (0.5 , 4.2)** | **160 (42 , 351)** | **1.6 (0.4 , 3.5)** | **2.9 (0.8 , 6.3)** | **49.9 (21 , 88.7)** |
| **Central Sub-Saharan Africa** | **481 (116 , 1225)** | **1.7 (0.4 , 4)** | **2.6 (0.6 , 6.4)** | **1492 (370 , 3423)** | **2.6 (0.7 , 5.7)** | **3.4 (0.8 , 7.6)** | **29.7 (3.4 , 66.4)** |
| **Angola** | **77 (18 , 180)** | **1.6 (0.4 , 3.5)** | **2.4 (0.6 , 5.4)** | **325 (83 , 723)** | **2.6 (0.7 , 5.3)** | **3.6 (0.9 , 7.8)** | **48.3 (16.4 , 100.8)** |
| **Central African Republic** | **25 (6 , 62)** | **1.5 (0.4 , 3.5)** | **2.5 (0.6 , 5.8)** | **57 (13 , 148)** | **2.1 (0.6 , 4.9)** | **3.1 (0.8 , 7.5)** | **23.8 (-3.7 , 56.8)** |
| **Congo** | **34 (8 , 79)** | **2 (0.5 , 4.4)** | **3.6 (0.9 , 8.2)** | **106 (27 , 236)** | **3.1 (0.9 , 6.7)** | **4.8 (1.3 , 10.4)** | **32.6 (4.5 , 69.6)** |
| **Democratic Republic of the Congo** | **320 (74 , 862)** | **1.7 (0.4 , 4.3)** | **2.5 (0.6 , 6.7)** | **922 (214 , 2246)** | **2.5 (0.7 , 5.7)** | **3.1 (0.7 , 7.4)** | **22.3 (-8.3 , 63.4)** |
| **Equatorial Guinea** | **4 (1 , 9)** | **1.6 (0.4 , 3.7)** | **2.2 (0.5 , 5.1)** | **21 (5 , 51)** | **3.6 (1 , 7.6)** | **5.5 (1.4 , 12.7)** | **153.2 (65 , 294.2)** |
| **Gabon** | **21 (5 , 50)** | **2.6 (0.7 , 5.8)** | **4.2 (1.1 , 9.6)** | **60 (15 , 134)** | **4.2 (1.1 , 8.7)** | **6.6 (1.7 , 14.5)** | **56.8 (22.3 , 96.7)** |
