# Supplementary material for: Global, Regional and National Burden of Cancers Attributable to High Fasting Plasma Glucose in 204 Countries and Territories, 1990-2019
Source: Front Endocrinol (Lausanne). 2022 Jul 19;13:879890. doi: 10.3389/fendo.2022.879890 (PMC9366927; doi:10.3389/fendo.2022.879890)
Supplement: Supplementary Table 3 — Number, proportion and age-standardized rates of disability-adjusted-life-years (DALYs) due to cancers attributable to high fasting plasma glucose (per 100,000) in 2019, by sex and location (Generated from data available from http://ghdx.healthdata.org/gbd-results-tool) [file Table_3.doc]

| **Table S3: Number, proportion and age-standardised rates of disability-adjusted-life-years (DALYs) due to cancers attributable to high fasting plasma glucose (per 100,000) in 2019, by sex and location**  **(Generated from data available from http://ghdx.healthdata.org/gbd-results-tool)** | | | | | | |
| --- | --- | --- | --- | --- | --- | --- |
|  | **Male** | | | **Female** | | |
|  | **No**  **(95% UI)** | **PAF**  **(95% UI)** | **ASRs per 100,000 (95% UI)** | **No**  **(95% UI)** | **PAF**  **(95% UI)** | **ASRs per 100,000 (95% UI)** |
| **Global** | **4599744 (1118147 , 9896338)** | **3.3 (0.8 , 7)** | **120.4 (29.5 , 257.7)** | **3981039 (1085159 , 8399117)** | **3.6 (1 , 7.5)** | **91 (24.8 , 192.1)** |
| **High-income North America** | **628566 (161646 , 1283548)** | **6.3 (1.6 , 12.8)** | **215.5 (55.5 , 439.9)** | **574972 (159912 , 1167095)** | **6.7 (1.9 , 13.5)** | **171.3 (47.3 , 348)** |
| **Canada** | **39056 (9245 , 84985)** | **3.8 (0.9 , 8.2)** | **119.3 (28.2 , 260.8)** | **36921 (9446 , 77956)** | **4.1 (1.1 , 8.6)** | **101 (25.9 , 213.6)** |
| **Greenland** | **106 (24 , 241)** | **4.4 (1 , 9.7)** | **279.9 (63.8 , 626)** | **54 (13 , 126)** | **3.3 (0.8 , 7.2)** | **167.8 (40.7 , 384.9)** |
| **United States of America** | **589393 (152876 , 1198802)** | **6.6 (1.7 , 13.3)** | **227.5 (58.8 , 463.5)** | **537988 (150360 , 1090384)** | **7 (1.9 , 14.2)** | **179.8 (50 , 364.3)** |
| **Australasia** | **23145 (5538 , 49652)** | **3.2 (0.8 , 6.7)** | **97.8 (23.3 , 209.8)** | **21548 (5611 , 45538)** | **3.7 (1 , 7.7)** | **82.2 (21.2 , 174.6)** |
| **Australia** | **19794 (4774 , 42450)** | **3.2 (0.8 , 6.9)** | **99.2 (24 , 214)** | **17767 (4644 , 37544)** | **3.7 (1 , 7.7)** | **80.2 (20.8 , 170.6)** |
| **New Zealand** | **3351 (802 , 7175)** | **2.9 (0.7 , 6.1)** | **90.3 (21.6 , 193.9)** | **3782 (966 , 8049)** | **3.6 (0.9 , 7.6)** | **92.3 (23.5 , 195.8)** |
| **High-income Asia Pacific** | **217990 (51863 , 467884)** | **3.7 (0.9 , 7.8)** | **107.6 (25.7 , 231.4)** | **120426 (31232 , 258432)** | **3.1 (0.8 , 6.5)** | **49.7 (12.7 , 105.9)** |
| **Brunei Darussalam** | **536 (147 , 1089)** | **7 (1.9 , 13.4)** | **460.9 (128.9 , 906.5)** | **532 (149 , 1071)** | **6.4 (1.9 , 12.7)** | **339.6 (95 , 677.1)** |
| **Japan** | **157832 (37216 , 339930)** | **3.5 (0.9 , 7.6)** | **99.9 (23.8 , 216)** | **85578 (21685 , 184794)** | **2.9 (0.7 , 6.1)** | **45.3 (11.5 , 97.6)** |
| **Singapore** | **4085 (972 , 8692)** | **4.7 (1.1 , 9.8)** | **109.8 (26.3 , 232.7)** | **3187 (826 , 6783)** | **4.3 (1.1 , 9.1)** | **79 (20.5 , 168)** |
| **Republic of Korea** | **55538 (13183 , 119069)** | **3.9 (0.9 , 8.4)** | **138.6 (33.1 , 295.8)** | **31129 (8147 , 66250)** | **3.7 (1 , 7.7)** | **62.6 (16.3 , 133.6)** |
| **Western Europe** | **695190 (175857 , 1458458)** | **4.9 (1.2 , 10.2)** | **170.9 (43.1 , 358.9)** | **598019 (163290 , 1220499)** | **5.5 (1.5 , 11.2)** | **123.6 (33.5 , 254.3)** |
| **Andorra** | **132 (31 , 295)** | **4 (1 , 8.5)** | **188.5 (44.6 , 420.6)** | **57 (14 , 131)** | **3.4 (0.9 , 7.2)** | **81.9 (20.6 , 187.8)** |
| **Austria** | **9525 (2372 , 20413)** | **3.9 (1 , 8.2)** | **120.9 (29.9 , 259.3)** | **8744 (2332 , 18546)** | **4.4 (1.1 , 9.2)** | **91 (24 , 193)** |
| **Belgium** | **16570 (3863 , 35660)** | **4.4 (1 , 9.5)** | **162.1 (37.5 , 350.3)** | **13648 (3626 , 28390)** | **4.8 (1.3 , 10.1)** | **112.8 (29.6 , 237.6)** |
| **Cyprus** | **1754 (432 , 3626)** | **6.4 (1.6 , 13.1)** | **182.7 (45.5 , 378.4)** | **1295 (347 , 2638)** | **6 (1.6 , 12)** | **124.9 (33.5 , 256.2)** |
| **Denmark** | **7112 (1773 , 15145)** | **3.7 (0.9 , 7.8)** | **128.8 (31.9 , 274.3)** | **8012 (2080 , 17124)** | **4.7 (1.2 , 10)** | **131 (33.6 , 280.7)** |
| **Finland** | **7183 (1828 , 15093)** | **4.8 (1.2 , 9.9)** | **128.4 (32.6 , 270.9)** | **7875 (2183 , 16184)** | **6.1 (1.7 , 12.5)** | **119.8 (32.6 , 249.9)** |
| **France** | **63114 (14465 , 139763)** | **2.8 (0.6 , 6.1)** | **107.8 (24.3 , 239.8)** | **46625 (11700 , 102648)** | **3 (0.8 , 6.4)** | **63.7 (16 , 140.4)** |
| **Germany** | **171743 (43974 , 358552)** | **5.8 (1.5 , 12)** | **202 (51.2 , 423.3)** | **165107 (45330 , 334829)** | **7.1 (1.9 , 14.2)** | **163.1 (44.3 , 333.1)** |
| **Greece** | **20741 (4840 , 44922)** | **5.3 (1.2 , 11.5)** | **201.6 (46.8 , 439.3)** | **11884 (3103 , 25131)** | **4.5 (1.2 , 9.4)** | **95.7 (24.7 , 203.8)** |
| **Iceland** | **286 (70 , 614)** | **3.6 (0.9 , 7.7)** | **107.7 (26.3 , 232.3)** | **242 (63 , 526)** | **4.3 (1.1 , 9.1)** | **85.5 (22.1 , 186.5)** |
| **Ireland** | **4751 (1175 , 10047)** | **4.2 (1 , 8.7)** | **132 (32.7 , 279.3)** | **4463 (1178 , 9497)** | **4.4 (1.2 , 9.1)** | **113.2 (29.9 , 241.3)** |
| **Israel** | **6757 (1677 , 14359)** | **4.2 (1.1 , 8.9)** | **127.5 (31.5 , 271.3)** | **7216 (1914 , 14935)** | **5 (1.3 , 10.3)** | **114.6 (30.3 , 238)** |
| **Italy** | **115261 (29078 , 237667)** | **5.6 (1.4 , 11.5)** | **183 (45.8 , 378.7)** | **86050 (23370 , 175726)** | **5.5 (1.5 , 11.1)** | **112.9 (30.6 , 231.3)** |
| **Luxembourg** | **947 (242 , 1983)** | **6.2 (1.6 , 12.8)** | **205.1 (52.3 , 429.5)** | **751 (203 , 1593)** | **6.3 (1.8 , 12.8)** | **142 (38.3 , 303)** |
| **Malta** | **688 (177 , 1449)** | **5.8 (1.5 , 12)** | **155.4 (39.6 , 327.8)** | **600 (164 , 1263)** | **6.8 (1.9 , 13.8)** | **123.2 (32.9 , 261.4)** |
| **Monaco** | **110 (25 , 242)** | **4.6 (1.1 , 10)** | **254.2 (58.1 , 565.6)** | **94 (24 , 210)** | **4.9 (1.3 , 10.2)** | **190.3 (48.7 , 430.8)** |
| **Netherlands** | **23001 (5497 , 50529)** | **3.8 (0.9 , 8.2)** | **141.3 (33.7 , 311)** | **20360 (5417 , 43500)** | **4 (1 , 8.4)** | **113.3 (30 , 243.3)** |
| **Norway** | **5765 (1461 , 12047)** | **4.3 (1.1 , 9)** | **126.4 (32.1 , 264.3)** | **6316 (1708 , 13246)** | **5.5 (1.5 , 11.3)** | **125.5 (33.8 , 262.5)** |
| **Portugal** | **19724 (5161 , 40924)** | **5.2 (1.3 , 10.7)** | **194.1 (49.9 , 407.8)** | **14756 (4028 , 30643)** | **6 (1.6 , 12.2)** | **112.5 (30.7 , 233.2)** |
| **San Marino** | **55 (13 , 135)** | **4.2 (1.1 , 8.8)** | **182.9 (43 , 448.6)** | **37 (8 , 100)** | **4 (1.1 , 8.5)** | **110 (25.5 , 304.9)** |
| **Spain** | **87627 (22318 , 185961)** | **5.8 (1.5 , 12.2)** | **211.5 (53 , 448.9)** | **50648 (13725 , 105639)** | **5.3 (1.4 , 10.8)** | **98.9 (26.7 , 207.5)** |
| **Sweden** | **9765 (2509 , 20538)** | **3.8 (1 , 7.9)** | **96.5 (24.6 , 203.7)** | **11329 (3029 , 23493)** | **4.8 (1.3 , 10)** | **103.9 (27.6 , 217.5)** |
| **Switzerland** | **8601 (2124 , 18461)** | **3.9 (1 , 8.3)** | **108.4 (26.7 , 233)** | **8475 (2261 , 17793)** | **4.8 (1.3 , 10)** | **92.7 (24.8 , 195.6)** |
| **United Kingdom** | **113373 (29309 , 231236)** | **5.7 (1.5 , 11.6)** | **194.6 (49.9 , 399.3)** | **122914 (34540 , 251361)** | **7 (2 , 14.2)** | **185.5 (51.3 , 380)** |
| **Southern Latin America** | **58087 (14471 , 122747)** | **3.8 (0.9 , 8.1)** | **156.6 (38.9 , 330.4)** | **58793 (15259 , 122255)** | **4.3 (1.1 , 8.9)** | **126.3 (32.6 , 263.5)** |
| **Argentina** | **41958 (10405 , 89920)** | **4 (1 , 8.6)** | **175.9 (43.7 , 376.3)** | **40808 (10304 , 85029)** | **4.4 (1.1 , 9)** | **135.8 (34 , 283.3)** |
| **Chile** | **11908 (3041 , 25359)** | **3.3 (0.8 , 7)** | **109.1 (27.8 , 232.4)** | **14803 (4038 , 30627)** | **4.4 (1.2 , 9.2)** | **110.9 (30.2 , 229.2)** |
| **Uruguay** | **4218 (1038 , 9152)** | **3.6 (0.9 , 7.8)** | **186.4 (45.5 , 405.9)** | **3180 (812 , 6874)** | **3.4 (0.9 , 7.2)** | **104 (26.4 , 225)** |
| **Eastern Europe** | **140018 (32567 , 313186)** | **2.2 (0.5 , 5)** | **103.8 (24 , 232.1)** | **123689 (30684 , 269158)** | **2.5 (0.6 , 5.4)** | **58.9 (14.5 , 129.5)** |
| **Belarus** | **6017 (1283 , 14351)** | **2 (0.5 , 4.6)** | **96.8 (20.9 , 230.4)** | **4236 (991 , 9812)** | **2.1 (0.5 , 4.5)** | **44.2 (10.3 , 102.7)** |
| **Estonia** | **1363 (311 , 3144)** | **3.1 (0.8 , 7)** | **137.5 (31.4 , 316.9)** | **1244 (317 , 2781)** | **3.4 (0.9 , 7.2)** | **79.8 (20.1 , 179.5)** |
| **Latvia** | **1997 (452 , 4651)** | **3 (0.7 , 6.5)** | **137.2 (31 , 319.3)** | **1925 (473 , 4424)** | **3.5 (0.9 , 7.3)** | **81.9 (19.7 , 188.7)** |
| **Lithuania** | **2424 (538 , 5535)** | **2.5 (0.6 , 5.5)** | **114 (25.3 , 260.9)** | **1840 (447 , 4081)** | **2.4 (0.6 , 5.2)** | **55.2 (13.1 , 123.8)** |
| **Republic of Moldova** | **3122 (744 , 6799)** | **3.1 (0.7 , 6.7)** | **126.1 (30.2 , 273.4)** | **2373 (573 , 5209)** | **3.5 (0.9 , 7.3)** | **69.8 (16.9 , 154.1)** |
| **Russian Federation** | **89169 (20163 , 203601)** | **2.2 (0.5 , 4.9)** | **97.3 (22.4 , 220.7)** | **85700 (21051 , 188935)** | **2.5 (0.6 , 5.4)** | **59.2 (14.3 , 131.9)** |
| **Ukraine** | **35925 (8154 , 84716)** | **2.2 (0.5 , 4.9)** | **121.2 (27.4 , 284.6)** | **26372 (6192 , 59971)** | **2.4 (0.6 , 5.3)** | **59.1 (13.9 , 135.4)** |
| **Central Europe** | **254582 (62263 , 551215)** | **5.5 (1.4 , 11.7)** | **270 (66.1 , 584.3)** | **166484 (44113 , 359220)** | **5.1 (1.4 , 10.5)** | **138.5 (36.8 , 299.9)** |
| **Albania** | **1806 (362 , 4448)** | **2.5 (0.5 , 5.8)** | **85.3 (17.2 , 211.3)** | **960 (233 , 2342)** | **2.3 (0.6 , 5)** | **42.6 (10.3 , 105.1)** |
| **Bosnia and Herzegovina** | **9154 (2183 , 20348)** | **7.1 (1.8 , 14.7)** | **330.2 (79.5 , 728.1)** | **5791 (1550 , 13045)** | **6.3 (1.8 , 12.7)** | **171.1 (45.5 , 385.1)** |
| **Bulgaria** | **15095 (3703 , 34588)** | **4.9 (1.2 , 10.4)** | **243.6 (59.2 , 559.2)** | **9617 (2424 , 21995)** | **4.4 (1.1 , 9.2)** | **123.2 (30.4 , 284.1)** |
| **Croatia** | **10195 (2510 , 23061)** | **5.7 (1.4 , 12)** | **267.2 (65.5 , 606.2)** | **6377 (1632 , 14289)** | **5.4 (1.5 , 11.1)** | **127.8 (32.3 , 290.1)** |
| **Czechia** | **30926 (8437 , 65309)** | **8.3 (2.2 , 16.4)** | **325.2 (88.3 , 689.4)** | **20930 (5710 , 45207)** | **7.5 (2.2 , 15.1)** | **177.6 (48.2 , 387.9)** |
| **Hungary** | **26773 (6640 , 58763)** | **6.2 (1.6 , 12.9)** | **337.5 (83.3 , 739.7)** | **19075 (4977 , 42766)** | **5.6 (1.5 , 11.6)** | **172.7 (44.1 , 391.4)** |
| **Montenegro** | **1564 (357 , 3524)** | **6.4 (1.5 , 13.8)** | **338.3 (77.5 , 761.6)** | **967 (260 , 2065)** | **6.1 (1.6 , 12.5)** | **178.2 (47.6 , 382)** |
| **North Macedonia** | **5316 (1303 , 11956)** | **6.4 (1.6 , 13.4)** | **327.5 (80.7 , 736.2)** | **3296 (866 , 7260)** | **6.1 (1.7 , 12.3)** | **190.5 (49.7 , 421)** |
| **Poland** | **88618 (20605 , 200516)** | **5.6 (1.4 , 11.9)** | **292.1 (68.1 , 661)** | **58122 (15350 , 125736)** | **5.2 (1.4 , 10.7)** | **146.3 (38.5 , 320.2)** |
| **Romania** | **28333 (6429 , 63823)** | **3.7 (0.9 , 8.1)** | **178.7 (40.6 , 402.9)** | **16634 (4159 , 36211)** | **3.3 (0.8 , 7.1)** | **82.1 (20.3 , 180.9)** |
| **Serbia** | **25266 (6127 , 56430)** | **6.7 (1.7 , 13.8)** | **338.2 (81.7 , 758.9)** | **17017 (4381 , 38295)** | **6.1 (1.7 , 12.3)** | **196.2 (50.8 , 443.9)** |
| **Slovakia** | **8125 (1949 , 18763)** | **4.2 (1 , 9)** | **200.4 (48.3 , 460.1)** | **5445 (1364 , 12630)** | **4 (1.1 , 8.5)** | **102.1 (25.7 , 236.7)** |
| **Slovenia** | **3411 (782 , 7667)** | **4.4 (1.1 , 9.4)** | **178 (40.8 , 401)** | **2254 (564 , 5054)** | **4.4 (1.2 , 9.3)** | **96.2 (23.6 , 221.4)** |
| **Central Asia** | **37454 (8654 , 82011)** | **2.6 (0.6 , 5.7)** | **114.2 (27.4 , 246.4)** | **38008 (9562 , 79629)** | **3 (0.8 , 6.3)** | **88.2 (22.4 , 183.8)** |
| **Armenia** | **3479 (792 , 7892)** | **4.2 (1 , 9.2)** | **188.7 (43.2 , 426.6)** | **2903 (718 , 6252)** | **4.6 (1.2 , 9.5)** | **121.1 (30.1 , 262.1)** |
| **Azerbaijan** | **5965 (1205 , 14013)** | **2.8 (0.6 , 6.3)** | **128.3 (26.8 , 297.6)** | **4938 (1245 , 10851)** | **3.2 (0.8 , 6.8)** | **89.6 (22.8 , 194.6)** |
| **Georgia** | **6029 (1399 , 13346)** | **4.8 (1.1 , 10.4)** | **244 (56.6 , 541.8)** | **4080 (1058 , 8777)** | **4.6 (1.2 , 9.7)** | **123.1 (31.8 , 265.2)** |
| **Kazakhstan** | **10127 (2318 , 22765)** | **3.2 (0.7 , 7.1)** | **139.5 (32.7 , 308.7)** | **11999 (3146 , 25564)** | **4.2 (1.1 , 8.7)** | **114.6 (30.1 , 243.6)** |
| **Kyrgyzstan** | **948 (208 , 2140)** | **1.4 (0.3 , 3.2)** | **47.1 (10.4 , 105.5)** | **1071 (267 , 2391)** | **1.8 (0.5 , 3.9)** | **40.3 (10.1 , 89.2)** |
| **Mongolia** | **743 (151 , 1794)** | **0.7 (0.2 , 1.7)** | **77.5 (15.7 , 186)** | **522 (123 , 1218)** | **0.7 (0.2 , 1.6)** | **40.9 (9.8 , 94.7)** |
| **Tajikistan** | **1589 (354 , 3651)** | **1.7 (0.4 , 3.7)** | **68.8 (15.9 , 155.4)** | **2125 (535 , 4663)** | **2.5 (0.6 , 5.1)** | **77.7 (19.9 , 168.7)** |
| **Turkmenistan** | **936 (202 , 2194)** | **1.4 (0.3 , 3.2)** | **51 (11.2 , 118.6)** | **1182 (280 , 2667)** | **2.1 (0.5 , 4.5)** | **51.2 (12.3 , 116)** |
| **Uzbekistan** | **7638 (1746 , 16874)** | **2 (0.5 , 4.4)** | **78.8 (18.2 , 170.4)** | **9188 (2235 , 19839)** | **2.3 (0.6 , 4.9)** | **74.4 (18.4 , 158.8)** |
| **Central Latin America** | **101228 (25693 , 214658)** | **3.5 (0.9 , 7)** | **93.8 (23.9 , 199.1)** | **135340 (36594 , 286641)** | **4.2 (1.2 , 8.6)** | **106 (28.8 , 224.3)** |
| **Colombia** | **17343 (4271 , 39048)** | **3 (0.8 , 6.2)** | **73 (18.1 , 164.1)** | **24614 (5959 , 56905)** | **3.8 (1 , 7.7)** | **85.6 (20.7 , 198.5)** |
| **Costa Rica** | **2578 (647 , 5695)** | **3.3 (0.9 , 6.8)** | **109.6 (27.6 , 241.9)** | **2704 (679 , 6200)** | **4 (1 , 8.2)** | **97.7 (24.4 , 223.3)** |
| **El Salvador** | **1892 (468 , 4170)** | **3 (0.8 , 6.2)** | **76.4 (18.8 , 169.1)** | **2819 (727 , 6645)** | **3.4 (0.9 , 7)** | **83.7 (21.6 , 197.7)** |
| **Guatemala** | **3410 (854 , 7503)** | **2.2 (0.6 , 4.5)** | **69.6 (17.5 , 152.8)** | **5674 (1514 , 12632)** | **2.8 (0.8 , 5.7)** | **93.5 (25.1 , 207)** |
| **Honduras** | **2823 (634 , 6626)** | **3.2 (0.7 , 7.4)** | **100.6 (22.7 , 235)** | **4656 (1191 , 10770)** | **3.7 (1 , 8.2)** | **144.7 (37.3 , 333.7)** |
| **Mexico** | **53404 (13937 , 113262)** | **3.8 (1 , 7.7)** | **98.8 (26 , 208.9)** | **71521 (19291 , 150345)** | **4.8 (1.3 , 9.6)** | **112.8 (30.6 , 237.6)** |
| **Nicaragua** | **1538 (379 , 3385)** | **2.7 (0.7 , 5.6)** | **81.4 (20 , 177.6)** | **2179 (591 , 4600)** | **3.5 (0.9 , 7.2)** | **90.3 (24.7 , 188.8)** |
| **Panama** | **1697 (428 , 3798)** | **3.4 (0.9 , 7)** | **85.1 (21.6 , 190.3)** | **2018 (523 , 4588)** | **4.1 (1.1 , 8.4)** | **95.3 (24.7 , 216.6)** |
| **Venezuela (Bolivarian Republic of)** | **16543 (3942 , 38628)** | **3.6 (0.9 , 7.6)** | **120.2 (28.9 , 279.6)** | **19155 (4793 , 45272)** | **4.2 (1.1 , 8.6)** | **122.5 (30.8 , 288.9)** |
| **Andean Latin America** | **12631 (3052 , 28137)** | **1.7 (0.4 , 3.5)** | **48.2 (11.7 , 107.4)** | **20161 (5115 , 43926)** | **2.3 (0.6 , 4.7)** | **69.9 (17.8 , 152.3)** |
| **Bolivia (Plurinational State of)** | **2862 (655 , 6848)** | **1.8 (0.4 , 4)** | **71 (16.5 , 169.5)** | **4326 (1071 , 10117)** | **2.1 (0.5 , 4.6)** | **94.1 (23.5 , 218.3)** |
| **Ecuador** | **4104 (982 , 9185)** | **2 (0.5 , 4.1)** | **58.7 (14.1 , 131.1)** | **6899 (1773 , 15016)** | **2.9 (0.8 , 6)** | **88.4 (22.7 , 192.4)** |
| **Peru** | **5665 (1317 , 13327)** | **1.5 (0.3 , 3.1)** | **37.5 (8.7 , 88.1)** | **8936 (2219 , 21011)** | **2 (0.5 , 4.2)** | **54.3 (13.5 , 127.5)** |
| **Caribbean** | **34667 (8874 , 73803)** | **3.9 (1 , 7.9)** | **142.9 (36.6 , 303.8)** | **37694 (9898 , 80812)** | **4.6 (1.2 , 9.3)** | **137.4 (36 , 294.6)** |
| **Antigua and Barbuda** | **47 (13 , 98)** | **2.9 (0.8 , 5.9)** | **100.1 (27.1 , 207.1)** | **100 (26 , 217)** | **6.1 (1.6 , 12.7)** | **184.6 (48.1 , 400.4)** |
| **Barbados** | **281 (72 , 607)** | **3.1 (0.8 , 6.5)** | **124.6 (32 , 268.6)** | **549 (140 , 1188)** | **6.1 (1.6 , 12.3)** | **203.7 (51.7 , 442.3)** |
| **Belize** | **104 (25 , 233)** | **2.3 (0.5 , 5)** | **74.8 (18.2 , 166.4)** | **131 (35 , 280)** | **3.2 (0.9 , 6.6)** | **94.1 (25.5 , 199.3)** |
| **Bermuda** | **89 (22 , 193)** | **4 (1 , 8.5)** | **153.1 (38.3 , 331.2)** | **70 (18 , 161)** | **5.1 (1.3 , 10.6)** | **94.7 (24 , 219.8)** |
| **Bahamas** | **244 (59 , 530)** | **3 (0.8 , 6.3)** | **137.3 (33.2 , 297)** | **377 (94 , 822)** | **4.9 (1.2 , 10.1)** | **171.9 (43.1 , 371)** |
| **Cuba** | **17759 (4199 , 38904)** | **5.1 (1.3 , 10.7)** | **198.6 (47 , 433.6)** | **14023 (3615 , 30496)** | **5.3 (1.4 , 11)** | **139.7 (35.9 , 303.8)** |
| **Dominica** | **84 (23 , 175)** | **3.5 (0.9 , 6.9)** | **187.7 (51 , 391.6)** | **82 (21 , 180)** | **5.1 (1.3 , 10.4)** | **179.6 (45.9 , 395.9)** |
| **Dominican Republic** | **2719 (599 , 6519)** | **1.8 (0.4 , 3.9)** | **61.5 (13.7 , 146.9)** | **3027 (745 , 7320)** | **2.3 (0.6 , 5)** | **62.7 (15.4 , 151.5)** |
| **Grenada** | **98 (28 , 200)** | **3.8 (1 , 7.8)** | **182.4 (51.4 , 371.7)** | **122 (33 , 250)** | **5.6 (1.5 , 11.2)** | **207 (56.7 , 426.2)** |
| **Guyana** | **359 (91 , 802)** | **3.5 (0.9 , 7.2)** | **119.9 (30.9 , 259.9)** | **695 (184 , 1555)** | **5.5 (1.5 , 11)** | **198.9 (52.9 , 439.8)** |
| **Haiti** | **2710 (581 , 6847)** | **1.9 (0.5 , 4.2)** | **85.7 (18.6 , 213.7)** | **6514 (1571 , 15258)** | **3.2 (0.8 , 7)** | **163.8 (40.5 , 377.1)** |
| **Jamaica** | **2337 (568 , 5179)** | **4.3 (1.1 , 9)** | **164.1 (40.2 , 362.5)** | **2732 (711 , 6135)** | **5.4 (1.4 , 10.9)** | **179.4 (46.7 , 402.6)** |
| **Puerto Rico** | **4379 (1165 , 9648)** | **5.8 (1.6 , 11.5)** | **141.2 (37.2 , 312.7)** | **5144 (1364 , 11415)** | **7.9 (2.2 , 15.7)** | **137.2 (36 , 305.8)** |
| **Saint Kitts and Nevis** | **49 (13 , 103)** | **3.4 (0.9 , 7.1)** | **152.9 (42.2 , 314)** | **63 (16 , 139)** | **5.8 (1.5 , 12)** | **183.7 (48.4 , 399)** |
| **Saint Lucia** | **138 (37 , 292)** | **3.3 (0.9 , 6.7)** | **136.3 (37.2 , 285.7)** | **219 (62 , 459)** | **6.9 (2 , 13.5)** | **192.3 (54.4 , 402.4)** |
| **Saint Vincent and the Grenadines** | **82 (22 , 171)** | **3 (0.8 , 6.1)** | **117.4 (31.3 , 242.7)** | **133 (35 , 281)** | **5.7 (1.5 , 11.3)** | **201 (53.5 , 423.7)** |
| **Suriname** | **473 (123 , 1011)** | **5 (1.3 , 10.1)** | **168.8 (44.4 , 356.9)** | **558 (153 , 1195)** | **5.8 (1.6 , 11.6)** | **170.6 (47.1 , 365.1)** |
| **Trinidad and Tobago** | **1330 (345 , 2973)** | **5.2 (1.4 , 10.4)** | **144.8 (37.5 , 324.5)** | **1670 (425 , 3737)** | **7 (1.9 , 13.7)** | **171.4 (43.2 , 384.2)** |
| **United States Virgin Islands** | **208 (53 , 442)** | **4.6 (1.2 , 9.3)** | **242.8 (61.8 , 516.3)** | **208 (56 , 440)** | **7.3 (2 , 14.7)** | **199.6 (52.7 , 427.2)** |
| **Tropical Latin America** | **91977 (23055 , 195478)** | **2.5 (0.6 , 5.3)** | **84.9 (21.4 , 180.1)** | **111940 (30272 , 236209)** | **3.4 (0.9 , 7)** | **83.8 (22.7 , 176.8)** |
| **Brazil** | **89542 (22438 , 190013)** | **2.5 (0.6 , 5.3)** | **84.7 (21.3 , 179.2)** | **109575 (29660 , 231134)** | **3.4 (0.9 , 7)** | **83.9 (22.7 , 176.9)** |
| **Paraguay** | **2434 (550 , 5554)** | **2.8 (0.7 , 6.1)** | **94.1 (21.3 , 213.2)** | **2366 (571 , 5540)** | **3 (0.8 , 6.3)** | **82.3 (20 , 191.8)** |
| **East Asia** | **1265113 (274434 , 2932381)** | **2.8 (0.6 , 6.2)** | **126.1 (27.8 , 288.8)** | **769417 (188633 , 1731263)** | **3.1 (0.8 , 6.6)** | **69.7 (17.1 , 156.3)** |
| **China** | **1221361 (263678 , 2821347)** | **2.8 (0.6 , 6.2)** | **125.7 (27.6 , 289.3)** | **733853 (177397 , 1672295)** | **3.1 (0.8 , 6.6)** | **68.9 (16.7 , 156.3)** |
| **Democratic People's Republic of Korea** | **13890 (2800 , 33243)** | **2.1 (0.5 , 4.9)** | **102.3 (20.8 , 241.5)** | **12250 (3081 , 27629)** | **2.5 (0.6 , 5.5)** | **65.5 (16.4 , 147.2)** |
| **Taiwan (Province of China)** | **29862 (6883 , 66811)** | **3.6 (0.9 , 7.5)** | **160.9 (37.2 , 357.6)** | **23314 (5898 , 51628)** | **4.7 (1.2 , 9.7)** | **109.5 (27.7 , 243.2)** |
| **Southeast Asia** | **281216 (64076 , 629204)** | **2.9 (0.7 , 6.3)** | **105.1 (24.2 , 232.6)** | **300144 (76802 , 655326)** | **3.3 (0.9 , 6.8)** | **91.1 (23.6 , 198.4)** |
| **Cambodia** | **7047 (1568 , 16140)** | **3.3 (0.8 , 7.2)** | **152.9 (34.6 , 342.7)** | **6151 (1626 , 13513)** | **2.8 (0.7 , 6)** | **88.6 (23.5 , 193.3)** |
| **Indonesia** | **93997 (20854 , 224112)** | **2.9 (0.7 , 6.4)** | **95.4 (20.9 , 222.8)** | **97615 (23609 , 232565)** | **2.8 (0.7 , 6.1)** | **83.3 (20.1 , 200.2)** |
| **Lao People's Democratic Republic** | **2493 (575 , 5809)** | **3.3 (0.8 , 7.2)** | **126.9 (30 , 291.8)** | **2750 (711 , 6253)** | **3.4 (0.9 , 7.4)** | **119.4 (31.3 , 266.9)** |
| **Malaysia** | **16903 (3976 , 37970)** | **3.8 (0.9 , 8.1)** | **132.8 (31.6 , 294.5)** | **19122 (4868 , 42803)** | **4.7 (1.2 , 9.7)** | **144.8 (37.2 , 320.8)** |
| **Maldives** | **76 (19 , 165)** | **2.3 (0.6 , 4.8)** | **54.8 (13.6 , 117.9)** | **86 (22 , 187)** | **3.3 (0.9 , 6.9)** | **66.7 (17.3 , 143.6)** |
| **Mauritius** | **1238 (331 , 2639)** | **6.6 (1.8 , 12.9)** | **153.8 (41.9 , 323.3)** | **1583 (420 , 3397)** | **7.8 (2.2 , 15.4)** | **164.2 (43.4 , 351.8)** |
| **Myanmar** | **22331 (4803 , 55038)** | **3 (0.7 , 6.9)** | **116.6 (25.7 , 283.8)** | **29141 (7798 , 62584)** | **3.7 (1 , 7.7)** | **111.2 (29.6 , 236.5)** |
| **Philippines** | **28137 (6406 , 67851)** | **2.2 (0.5 , 4.8)** | **80.2 (18.5 , 190.4)** | **39629 (9706 , 90295)** | **3.1 (0.8 , 6.4)** | **93.8 (23.1 , 210)** |
| **Sri Lanka** | **9712 (2396 , 22181)** | **4 (1 , 8.1)** | **84.9 (21.6 , 193.3)** | **13138 (3422 , 29362)** | **5.5 (1.5 , 10.8)** | **91.4 (24 , 204.6)** |
| **Seychelles** | **119 (30 , 247)** | **4.2 (1.1 , 8.3)** | **239 (62.3 , 486.7)** | **122 (33 , 259)** | **6.2 (1.7 , 12.3)** | **212.5 (58.2 , 449.4)** |
| **Thailand** | **44270 (10190 , 106104)** | **2.6 (0.6 , 5.8)** | **96 (21.9 , 227.9)** | **41574 (10181 , 98434)** | **3.4 (0.9 , 7)** | **74.4 (18.3 , 176.1)** |
| **Timor-Leste** | **366 (78 , 835)** | **3.1 (0.7 , 6.9)** | **92.4 (20.3 , 210.3)** | **336 (84 , 759)** | **2.9 (0.7 , 6.4)** | **81.7 (20.4 , 184)** |
| **Viet Nam** | **54158 (12002 , 125146)** | **3 (0.7 , 6.7)** | **143.1 (32.8 , 322.9)** | **48503 (12469 , 107652)** | **3.6 (0.9 , 7.5)** | **93.3 (24.4 , 205.4)** |
| **Oceania** | **5625 (1288 , 13039)** | **4.2 (1 , 9.1)** | **153.1 (35 , 347.3)** | **8203 (1870 , 19066)** | **4.9 (1.1 , 10.4)** | **204.3 (48 , 462.2)** |
| **American Samoa** | **75 (20 , 150)** | **8.2 (2.3 , 16.1)** | **318.8 (86.3 , 626.1)** | **94 (27 , 193)** | **10.3 (3.1 , 19.9)** | **365.5 (105 , 744.1)** |
| **Cook Islands** | **29 (7 , 61)** | **6.4 (1.5 , 13.1)** | **231.5 (55.3 , 497.5)** | **28 (7 , 62)** | **10.2 (2.7 , 20.2)** | **226.9 (58.8 , 496)** |
| **Micronesia (Federated States of)** | **86 (18 , 217)** | **5 (1.2 , 11)** | **247 (54.9 , 601.4)** | **116 (28 , 277)** | **6.4 (1.7 , 13.9)** | **291.2 (73.9 , 684.4)** |
| **Fiji** | **555 (159 , 1170)** | **5.4 (1.6 , 10.5)** | **163.3 (47.5 , 330.4)** | **1080 (287 , 2392)** | **7.3 (2 , 14.9)** | **260.4 (70.3 , 572.5)** |
| **Guam** | **158 (38 , 352)** | **4.9 (1.2 , 10.5)** | **169 (40.7 , 373.6)** | **126 (33 , 279)** | **5.8 (1.6 , 12)** | **129.8 (33.9 , 287.8)** |
| **Kiribati** | **73 (17 , 167)** | **3.7 (0.9 , 7.9)** | **230.3 (56.1 , 512.4)** | **77 (18 , 175)** | **3.1 (0.7 , 6.7)** | **179.9 (43.4 , 408.9)** |
| **Marshall Islands** | **56 (13 , 138)** | **6.5 (1.6 , 14.3)** | **301.7 (69.3 , 731.8)** | **76 (21 , 171)** | **8 (2.3 , 16.4)** | **387.7 (111.1 , 849.2)** |
| **Nauru** | **6 (1 , 14)** | **4 (0.9 , 8.8)** | **286.5 (62.8 , 680.2)** | **7 (2 , 17)** | **4.7 (1.2 , 10.1)** | **272.4 (67.3 , 621.4)** |
| **Niue** | **3 (1 , 6)** | **8 (2.1 , 16.2)** | **296.5 (76.1 , 625.5)** | **4 (1 , 8)** | **10.2 (3 , 20)** | **303.4 (84.6 , 665.4)** |
| **Northern Mariana Islands** | **83 (19 , 177)** | **6.2 (1.5 , 13.2)** | **304.5 (72.1 , 646.7)** | **58 (16 , 123)** | **6.3 (1.7 , 12.9)** | **213.9 (58.3 , 453.1)** |
| **Palau** | **25 (5 , 55)** | **4.8 (1.1 , 10.2)** | **222.6 (49.5 , 478.4)** | **45 (13 , 97)** | **8.9 (2.6 , 17.7)** | **409.6 (116 , 859.9)** |
| **Papua New Guinea** | **3514 (752 , 8586)** | **4 (0.9 , 8.8)** | **137.8 (29.7 , 332.5)** | **5108 (1100 , 12121)** | **4.6 (1 , 9.9)** | **182.3 (40 , 421.5)** |
| **Samoa** | **74 (19 , 165)** | **3.5 (0.9 , 7.4)** | **102.5 (26.3 , 223.6)** | **150 (39 , 342)** | **5.9 (1.6 , 12.6)** | **195.8 (51.1 , 445.3)** |
| **Solomon Islands** | **387 (75 , 1046)** | **3.9 (0.9 , 9.2)** | **236.8 (48.1 , 623.1)** | **610 (126 , 1396)** | **4.2 (0.9 , 9.3)** | **305.6 (66.9 , 692.5)** |
| **Tokelau** | **1 (0 , 2)** | **5.3 (1.3 , 11.4)** | **139.1 (32.6 , 327.9)** | **2 (0 , 4)** | **6.7 (1.8 , 13.6)** | **247.5 (62.9 , 544.2)** |
| **Tonga** | **74 (17 , 163)** | **3.9 (0.9 , 8.2)** | **204.2 (47.2 , 448.9)** | **81 (21 , 179)** | **5.6 (1.5 , 11.7)** | **190.7 (49 , 424.1)** |
| **Tuvalu** | **9 (2 , 20)** | **4.8 (1.1 , 10.2)** | **181.5 (41.1 , 411.7)** | **13 (3 , 30)** | **6.6 (1.8 , 13.7)** | **241.9 (62.6 , 540.4)** |
| **Vanuatu** | **153 (32 , 360)** | **4 (0.9 , 8.8)** | **167 (35.2 , 391.3)** | **141 (35 , 326)** | **4.4 (1.1 , 9.4)** | **161.1 (41.2 , 364.3)** |
| **North Africa and Middle East** | **252907 (62201 , 542268)** | **3.7 (0.9 , 7.8)** | **117.7 (29.4 , 249.3)** | **209243 (55272 , 441450)** | **3.9 (1 , 7.9)** | **96.5 (26 , 202.7)** |
| **Afghanistan** | **5298 (1255 , 13154)** | **1.7 (0.4 , 3.8)** | **97.8 (23.5 , 238.2)** | **8347 (2072 , 19157)** | **1.8 (0.5 , 3.8)** | **116.5 (30 , 259.9)** |
| **Algeria** | **13105 (3202 , 29132)** | **3.9 (1 , 8.1)** | **79.2 (19.4 , 175.5)** | **14494 (3784 , 30828)** | **4.3 (1.1 , 8.8)** | **86.7 (23.1 , 181.8)** |
| **Bahrain** | **1078 (298 , 2255)** | **7.6 (2.2 , 14.7)** | **238 (67 , 476.4)** | **944 (277 , 1895)** | **8.9 (2.6 , 17.2)** | **259.4 (79.7 , 503.3)** |
| **Egypt** | **29315 (7113 , 68027)** | **2.6 (0.7 , 5.6)** | **80.3 (19.6 , 185.7)** | **25609 (6451 , 61594)** | **3.7 (1 , 7.8)** | **84.5 (21.5 , 202)** |
| **Iran (Islamic Republic of)** | **28070 (7005 , 58977)** | **2.8 (0.7 , 5.9)** | **80 (20 , 167.5)** | **30876 (8400 , 63399)** | **3.9 (1.1 , 7.9)** | **84.1 (23.1 , 171.4)** |
| **Iraq** | **17039 (4294 , 37318)** | **4.4 (1.1 , 9.2)** | **160.6 (40.5 , 346.9)** | **18488 (4656 , 41516)** | **4.7 (1.2 , 9.6)** | **147.9 (38.1 , 326)** |
| **Jordan** | **4396 (1057 , 9611)** | **4.8 (1.2 , 9.8)** | **134.2 (33.4 , 287.7)** | **3500 (885 , 7576)** | **4.3 (1.1 , 8.9)** | **112.2 (28.9 , 240.6)** |
| **Kuwait** | **1613 (429 , 3441)** | **5.9 (1.6 , 11.6)** | **123 (33.2 , 257.8)** | **1134 (298 , 2473)** | **5.6 (1.5 , 11.5)** | **105.7 (28.5 , 221.7)** |
| **Lebanon** | **5577 (1388 , 11955)** | **6.1 (1.6 , 12.4)** | **237.9 (59.2 , 510.6)** | **5959 (1553 , 13041)** | **6.4 (1.7 , 13.1)** | **209.4 (54.7 , 457.2)** |
| **Libya** | **4966 (1193 , 11009)** | **5.7 (1.5 , 11.8)** | **201.1 (49.4 , 436.1)** | **3871 (986 , 8648)** | **5.1 (1.4 , 10.7)** | **148.3 (38.4 , 323)** |
| **Morocco** | **18729 (4197 , 43669)** | **4.9 (1.1 , 10.6)** | **118.4 (26.8 , 274.8)** | **19351 (4713 , 44732)** | **4.5 (1.1 , 9.6)** | **112.8 (28.1 , 259.5)** |
| **Palestine** | **2581 (669 , 5383)** | **5.6 (1.5 , 11.4)** | **243.5 (64.6 , 502)** | **2107 (549 , 4470)** | **4.9 (1.3 , 10.1)** | **174.1 (45.5 , 366.8)** |
| **Oman** | **714 (183 , 1622)** | **2.7 (0.7 , 5.8)** | **91.8 (24.7 , 196.2)** | **786 (203 , 1659)** | **4.1 (1.1 , 8.5)** | **112.9 (29.8 , 234.8)** |
| **Qatar** | **1093 (295 , 2362)** | **5.8 (1.6 , 11.5)** | **217.8 (62.3 , 437.5)** | **881 (256 , 1799)** | **8.9 (2.6 , 16.9)** | **438.3 (137 , 851)** |
| **Saudi Arabia** | **9328 (2344 , 20429)** | **3.8 (1 , 7.7)** | **92.1 (24.7 , 195.2)** | **9684 (2501 , 21216)** | **4.5 (1.2 , 9.3)** | **118.2 (32.1 , 247.2)** |
| **Sudan** | **7059 (1708 , 17046)** | **2.3 (0.6 , 5)** | **73.8 (18.3 , 176.5)** | **6834 (1645 , 16121)** | **2.7 (0.7 , 5.8)** | **75.8 (18.6 , 174.3)** |
| **Syrian Arab Republic** | **4626 (1063 , 10449)** | **3.3 (0.8 , 7.1)** | **72.1 (16.8 , 161.4)** | **4385 (1112 , 9811)** | **3.7 (1 , 7.6)** | **72.1 (18.5 , 158.3)** |
| **Tunisia** | **11294 (2610 , 26478)** | **7.5 (1.9 , 15.7)** | **182.4 (42.6 , 425.8)** | **5869 (1462 , 13856)** | **5.3 (1.4 , 10.9)** | **89.4 (22.4 , 210.9)** |
| **Turkey** | **77923 (17338 , 175702)** | **4.6 (1.1 , 10.1)** | **186.3 (41.6 , 418.1)** | **39316 (10132 , 86512)** | **4 (1.1 , 8.3)** | **84.2 (21.7 , 185.2)** |
| **United Arab Emirates** | **5503 (1364 , 12359)** | **3.6 (1 , 7.4)** | **241.8 (65.4 , 505)** | **2930 (755 , 6328)** | **5.2 (1.4 , 10.7)** | **277.9 (75.9 , 576.3)** |
| **Yemen** | **3343 (748 , 8226)** | **1.5 (0.4 , 3.5)** | **54 (12.4 , 131.5)** | **3668 (881 , 8626)** | **1.8 (0.5 , 3.9)** | **51.7 (12.8 , 122.5)** |
| **South Asia** | **391188 (95070 , 858370)** | **2.1 (0.5 , 4.5)** | **56.5 (13.8 , 123.6)** | **540448 (139031 , 1184439)** | **2.9 (0.7 , 6.1)** | **73.8 (19.1 , 161.4)** |
| **Bangladesh** | **21874 (4459 , 57879)** | **1.3 (0.3 , 3)** | **32.3 (6.7 , 85.2)** | **29310 (6822 , 67722)** | **2.1 (0.5 , 4.5)** | **45.1 (10.8 , 103)** |
| **Bhutan** | **117 (26 , 277)** | **1.7 (0.4 , 3.8)** | **41.9 (9.6 , 98.2)** | **150 (36 , 348)** | **2.4 (0.6 , 5.1)** | **54.7 (13.1 , 126.2)** |
| **India** | **305269 (75149 , 674905)** | **2.3 (0.6 , 4.8)** | **55 (13.6 , 120.9)** | **404764 (106013 , 886509)** | **2.9 (0.8 , 6.2)** | **68 (17.7 , 148.9)** |
| **Nepal** | **4609 (1034 , 10593)** | **1.4 (0.3 , 3.2)** | **43.7 (9.8 , 99.5)** | **7488 (1774 , 17348)** | **2.4 (0.6 , 5.2)** | **62.5 (15.1 , 143.6)** |
| **Pakistan** | **59320 (12624 , 140771)** | **1.9 (0.5 , 4.2)** | **104.2 (22.8 , 242.8)** | **98737 (23128 , 233444)** | **3.1 (0.7 , 6.7)** | **171.2 (40.6 , 402.2)** |
| **Southern Sub-Saharan Africa** | **25713 (6237 , 55318)** | **2.5 (0.6 , 5.3)** | **113.1 (27.7 , 239.6)** | **37648 (9837 , 77990)** | **3.7 (1 , 7.6)** | **116.7 (30.8 , 240.9)** |
| **Botswana** | **787 (182 , 1788)** | **2.6 (0.6 , 5.8)** | **144.2 (34.1 , 319.4)** | **1085 (269 , 2517)** | **3.4 (0.9 , 7.3)** | **142.6 (36.1 , 324.4)** |
| **Lesotho** | **600 (126 , 1458)** | **1.7 (0.4 , 3.9)** | **118 (25.8 , 279.9)** | **823 (193 , 1992)** | **2.6 (0.7 , 5.7)** | **110.2 (25.9 , 265)** |
| **Namibia** | **298 (74 , 674)** | **1.4 (0.3 , 2.9)** | **54 (13.5 , 120.4)** | **711 (168 , 1680)** | **2.9 (0.7 , 6.1)** | **88.1 (21.2 , 207.4)** |
| **South Africa** | **21030 (5041 , 45157)** | **2.7 (0.7 , 5.8)** | **115.7 (28.3 , 247.2)** | **27919 (7285 , 58521)** | **4 (1.1 , 8.2)** | **109 (28.7 , 227.1)** |
| **Eswatini** | **345 (79 , 835)** | **2 (0.5 , 4.5)** | **157.3 (36.6 , 375.4)** | **399 (97 , 948)** | **3.2 (0.8 , 6.7)** | **118 (28.8 , 277.4)** |
| **Zimbabwe** | **2653 (664 , 5851)** | **1.7 (0.4 , 3.7)** | **96.2 (24.3 , 209)** | **6711 (1722 , 14981)** | **3.1 (0.8 , 6.4)** | **166.5 (43.3 , 369.9)** |
| **Western Sub-Saharan Africa** | **35949 (8811 , 80358)** | **1.2 (0.3 , 2.6)** | **45.2 (11.2 , 99.2)** | **56066 (12895 , 124295)** | **1.8 (0.4 , 3.8)** | **59.1 (13.9 , 129.7)** |
| **Benin** | **1172 (272 , 2684)** | **1.4 (0.3 , 3)** | **58.3 (13.7 , 131.1)** | **1443 (354 , 3215)** | **1.7 (0.4 , 3.7)** | **57.9 (14.4 , 126.7)** |
| **Burkina Faso** | **1871 (428 , 4345)** | **1.2 (0.3 , 2.6)** | **50.5 (11.8 , 115.7)** | **2677 (641 , 5962)** | **1.5 (0.4 , 3.2)** | **55.1 (13.6 , 120.1)** |
| **Cameroon** | **3780 (902 , 8756)** | **1.7 (0.4 , 3.7)** | **74.3 (18 , 168.9)** | **4301 (1003 , 10044)** | **1.9 (0.5 , 4.2)** | **73 (17.3 , 168.2)** |
| **Cabo Verde** | **173 (41 , 395)** | **1.7 (0.4 , 3.8)** | **106.3 (25.2 , 241.4)** | **238 (63 , 503)** | **3.6 (1 , 7.5)** | **101.1 (27 , 213.7)** |
| **Chad** | **1393 (313 , 3321)** | **1.3 (0.3 , 2.8)** | **50.2 (11.5 , 119.2)** | **1219 (292 , 2800)** | **1.3 (0.3 , 2.7)** | **49.1 (11.8 , 113.8)** |
| **CÃ´te d'Ivoire** | **3055 (691 , 7078)** | **1.5 (0.4 , 3.3)** | **64.4 (15 , 146.5)** | **3066 (748 , 6872)** | **1.8 (0.5 , 3.9)** | **64.1 (16.2 , 142.1)** |
| **Gambia** | **184 (44 , 407)** | **0.9 (0.2 , 2.1)** | **42.8 (10.3 , 94.8)** | **246 (60 , 572)** | **1.7 (0.4 , 3.8)** | **51.4 (12.6 , 118.8)** |
| **Ghana** | **4469 (1113 , 9892)** | **1.7 (0.4 , 3.8)** | **69.7 (17.6 , 152)** | **8364 (2001 , 18716)** | **2.7 (0.6 , 5.8)** | **93.8 (22.5 , 207.5)** |
| **Guinea** | **1459 (337 , 3297)** | **1 (0.3 , 2.3)** | **55.7 (13 , 126.2)** | **1532 (370 , 3465)** | **1.2 (0.3 , 2.8)** | **57.7 (14.1 , 129.9)** |
| **Guinea-Bissau** | **200 (43 , 497)** | **1.3 (0.3 , 3)** | **68.5 (15.2 , 167.8)** | **277 (64 , 646)** | **1.6 (0.4 , 3.5)** | **70.4 (16.5 , 162.2)** |
| **Liberia** | **513 (119 , 1180)** | **1.7 (0.4 , 3.6)** | **57 (13.5 , 129.4)** | **691 (161 , 1601)** | **2.1 (0.5 , 4.6)** | **71.8 (17.1 , 163.2)** |
| **Mali** | **2085 (494 , 4789)** | **1.3 (0.3 , 2.8)** | **51.8 (12.2 , 118.1)** | **2383 (573 , 5331)** | **1.7 (0.4 , 3.7)** | **58.1 (14 , 130.4)** |
| **Mauritania** | **296 (66 , 703)** | **1.2 (0.3 , 2.6)** | **30.4 (6.8 , 71.7)** | **581 (138 , 1343)** | **1.9 (0.5 , 4.1)** | **58.4 (14.1 , 133.2)** |
| **Niger** | **918 (193 , 2268)** | **0.8 (0.2 , 1.8)** | **27.6 (6 , 68)** | **1057 (238 , 2594)** | **0.9 (0.2 , 1.9)** | **27.5 (6.3 , 67.3)** |
| **Nigeria** | **10658 (2349 , 25708)** | **0.9 (0.2 , 2.2)** | **30.2 (6.6 , 72.1)** | **23082 (4926 , 54084)** | **1.7 (0.4 , 3.7)** | **52.3 (11.4 , 121.4)** |
| **Sao Tome and Principe** | **47 (11 , 106)** | **2.2 (0.5 , 4.9)** | **107.4 (26 , 237.7)** | **44 (11 , 109)** | **1.9 (0.5 , 4.4)** | **82.4 (19.9 , 202.9)** |
| **Senegal** | **2601 (624 , 5970)** | **2.2 (0.6 , 4.9)** | **78.2 (19.2 , 178.1)** | **3312 (825 , 7337)** | **2.7 (0.7 , 5.8)** | **86.8 (21.7 , 189.7)** |
| **Sierra Leone** | **467 (102 , 1119)** | **0.8 (0.2 , 1.8)** | **28.9 (6.4 , 68.8)** | **713 (167 , 1643)** | **1.1 (0.3 , 2.6)** | **41.1 (9.7 , 94.4)** |
| **Togo** | **608 (134 , 1436)** | **1.1 (0.2 , 2.4)** | **45.7 (10.4 , 107.1)** | **840 (197 , 1931)** | **1.3 (0.3 , 2.9)** | **41.9 (10 , 96.2)** |
| **Eastern Sub-Saharan Africa** | **26907 (6505 , 60237)** | **0.9 (0.2 , 1.9)** | **38.2 (9.4 , 84.2)** | **35185 (8445 , 78530)** | **1 (0.2 , 2.2)** | **41.4 (10 , 91.2)** |
| **Burundi** | **734 (169 , 1799)** | **0.8 (0.2 , 1.7)** | **33.9 (8 , 81.8)** | **788 (172 , 1951)** | **0.8 (0.2 , 1.8)** | **35.3 (7.8 , 86.9)** |
| **Comoros** | **73 (17 , 170)** | **1.1 (0.3 , 2.4)** | **34.8 (8.2 , 80.9)** | **111 (26 , 255)** | **1.2 (0.3 , 2.8)** | **41.9 (9.7 , 95.7)** |
| **Djibouti** | **155 (35 , 410)** | **1.2 (0.3 , 2.6)** | **53.4 (12.4 , 137.3)** | **147 (34 , 353)** | **1.2 (0.3 , 2.7)** | **53.7 (12.8 , 124.8)** |
| **Eritrea** | **488 (109 , 1125)** | **0.9 (0.2 , 2)** | **47.2 (10.9 , 109)** | **871 (201 , 2040)** | **1.2 (0.3 , 2.6)** | **55.8 (13.1 , 128.7)** |
| **Ethiopia** | **4683 (1035 , 11482)** | **0.7 (0.2 , 1.7)** | **24.2 (5.4 , 59.1)** | **6156 (1426 , 14658)** | **0.8 (0.2 , 1.8)** | **30.6 (7.2 , 72.3)** |
| **Kenya** | **3150 (748 , 7284)** | **0.9 (0.2 , 2)** | **33.4 (7.8 , 75.4)** | **4041 (925 , 9747)** | **1 (0.2 , 2.3)** | **34 (7.9 , 81)** |
| **Madagascar** | **1346 (302 , 3342)** | **0.8 (0.2 , 1.8)** | **28.4 (6.4 , 68.9)** | **2200 (504 , 5469)** | **1 (0.2 , 2.2)** | **37.1 (8.5 , 90.6)** |
| **Malawi** | **1581 (379 , 3521)** | **0.8 (0.2 , 1.9)** | **54.8 (13.5 , 121.8)** | **1828 (437 , 4124)** | **1 (0.2 , 2.3)** | **46.5 (11.2 , 103.9)** |
| **Mozambique** | **2399 (569 , 5508)** | **1.1 (0.3 , 2.5)** | **55.8 (13.6 , 126.3)** | **2958 (700 , 6951)** | **1.1 (0.3 , 2.4)** | **49 (11.7 , 113.6)** |
| **Rwanda** | **957 (211 , 2374)** | **0.9 (0.2 , 2.1)** | **42.6 (9.6 , 103.1)** | **1540 (366 , 3522)** | **1.2 (0.3 , 2.6)** | **44.8 (10.7 , 100.3)** |
| **Somalia** | **868 (176 , 2288)** | **0.6 (0.1 , 1.5)** | **33.9 (7.1 , 87.9)** | **1182 (250 , 3152)** | **0.7 (0.2 , 1.6)** | **30.5 (6.6 , 81.8)** |
| **South Sudan** | **790 (172 , 1913)** | **1.1 (0.2 , 2.5)** | **43.3 (9.5 , 103.8)** | **672 (153 , 1658)** | **1 (0.2 , 2.1)** | **37.4 (8.9 , 88.8)** |
| **United Republic of Tanzania** | **4296 (963 , 10485)** | **0.8 (0.2 , 1.8)** | **40 (9.1 , 96.7)** | **5402 (1306 , 11901)** | **1 (0.2 , 2.2)** | **43 (10.5 , 94.2)** |
| **Uganda** | **3520 (818 , 7839)** | **0.9 (0.2 , 2)** | **62.3 (14.9 , 136.5)** | **5323 (1261 , 11868)** | **1.5 (0.4 , 3.3)** | **67.5 (16.3 , 149.3)** |
| **Zambia** | **1848 (420 , 4211)** | **1.1 (0.3 , 2.4)** | **64.4 (15.2 , 145.4)** | **1937 (467 , 4515)** | **1.1 (0.3 , 2.5)** | **55.4 (13.5 , 127.5)** |
| **Central Sub-Saharan Africa** | **19592 (4259 , 51540)** | **2.2 (0.5 , 5.2)** | **86.6 (19.7 , 223.6)** | **17611 (4145 , 40917)** | **1.8 (0.4 , 3.9)** | **59.6 (14.4 , 137.5)** |
| **Angola** | **4654 (1071 , 10604)** | **2.2 (0.5 , 4.7)** | **97.3 (22.8 , 213.1)** | **3588 (867 , 8364)** | **1.6 (0.4 , 3.6)** | **57.3 (14.2 , 130.2)** |
| **Central African Republic** | **893 (170 , 2544)** | **1.9 (0.4 , 4.6)** | **90.4 (18.6 , 248.8)** | **695 (155 , 1732)** | **1.5 (0.3 , 3.4)** | **55.4 (12.6 , 134.4)** |
| **Congo** | **1241 (290 , 2850)** | **2.6 (0.6 , 5.9)** | **105.5 (25.2 , 235.1)** | **1397 (306 , 3373)** | **2.4 (0.6 , 5.4)** | **99.6 (22.6 , 235.3)** |
| **Democratic Republic of the Congo** | **11745 (2379 , 35312)** | **2.1 (0.5 , 5.5)** | **78 (16.6 , 229.1)** | **11101 (2593 , 26231)** | **1.7 (0.4 , 3.8)** | **56 (13.1 , 130.4)** |
| **Equatorial Guinea** | **229 (51 , 551)** | **2.7 (0.7 , 6.1)** | **125.1 (29.4 , 295.5)** | **265 (59 , 685)** | **2.7 (0.7 , 6)** | **97.2 (22.2 , 244.5)** |
| **Gabon** | **829 (189 , 1924)** | **3.6 (0.9 , 8)** | **173.6 (41 , 398)** | **565 (137 , 1325)** | **3.1 (0.8 , 6.8)** | **102.6 (25.1 , 240.1)** |
