# Supplementary material for: Global, Regional and National Burden of Cancers Attributable to High Fasting Plasma Glucose in 204 Countries and Territories, 1990-2019
Source: Front Endocrinol (Lausanne). 2022 Jul 19;13:879890. doi: 10.3389/fendo.2022.879890 (PMC9366927; doi:10.3389/fendo.2022.879890)
Supplement: Supplementary Table 4 — Number, proportion and age-standardized rates of disability-adjusted-life-years (DALYs) due to cancers attributable to high fasting plasma glucose (per 100,000) in 1990 and 2019, by location (Generated from data available from http://ghdx.healthdata.org/gbd-results-tool) [file Table_4.doc]

| **Table S4: Number, proportion and age-standardised rates of disability-adjusted-life-years (DALYs) due to cancers attributable to high fasting plasma glucose (per 100,000) in 1990 and 2019, by location**  **(Generated from data available from http://ghdx.healthdata.org/gbd-results-tool)** | | | | | | | |
| --- | --- | --- | --- | --- | --- | --- | --- |
|  | **1990** | | | **2019** | | | **% change in ASRs per 100,000**  **1990-2019** |
|  | **No**  **(95% UI)** | **PAF**  **(95% UI)** | **ASRs per 100,000 (95% UI)** | **No**  **(95% UI)** | **PAF**  **(95% UI)** | **ASRs per 100,000 (95% UI)** |
| **Global** | **3287639 (846221 , 6862885)** | **2 (0.5 , 4.1)** | **83.7 (21.8 , 174.2)** | **8580783 (2357076 , 17568241)** | **3.4 (0.9 , 6.9)** | **104.2 (28.7 , 212.9)** | **24.5 (16.4 , 35.6)** |
| **High-income North America** | **630270 (166937 , 1294023)** | **4.5 (1.2 , 9.2)** | **182.7 (48.2 , 376.8)** | **1203538 (343541 , 2366869)** | **6.5 (1.9 , 12.8)** | **191.4 (54.4 , 376.8)** | **4.8 (-1.2 , 16.1)** |
| **Canada** | **28699 (7350 , 60331)** | **2.2 (0.6 , 4.7)** | **86.8 (22.2 , 182.6)** | **75977 (20376 , 157591)** | **3.9 (1 , 8.1)** | **109.3 (29.3 , 226.7)** | **25.9 (13.9 , 43.8)** |
| **Greenland** | **41 (10 , 90)** | **1.4 (0.3 , 3.1)** | **121.4 (28.9 , 268.8)** | **161 (41 , 358)** | **4 (1 , 8.3)** | **227.5 (59.2 , 500.4)** | **87.4 (51.3 , 135)** |
| **United States of America** | **601516 (159712 , 1234381)** | **4.7 (1.2 , 9.7)** | **192.9 (51 , 397.8)** | **1127381 (322479 , 2210422)** | **6.8 (1.9 , 13.3)** | **201.4 (57.5 , 395.3)** | **4.4 (-1.8 , 15.9)** |
| **Australasia** | **18487 (4728 , 38766)** | **2 (0.5 , 4.2)** | **78.2 (19.9 , 164.2)** | **44694 (12088 , 91895)** | **3.4 (0.9 , 7)** | **89.4 (24.2 , 184.5)** | **14.3 (5.2 , 29.1)** |
| **Australia** | **15166 (3869 , 31830)** | **2 (0.5 , 4.2)** | **76.9 (19.7 , 161.3)** | **37561 (10096 , 77569)** | **3.4 (0.9 , 7)** | **89.1 (23.9 , 184)** | **15.7 (5 , 32.7)** |
| **New Zealand** | **3321 (852 , 6977)** | **2 (0.5 , 4.3)** | **84.9 (21.7 , 178.8)** | **7133 (1941 , 14827)** | **3.2 (0.9 , 6.7)** | **91.3 (24.7 , 190.7)** | **7.5 (-0.6 , 20)** |
| **High-income Asia Pacific** | **152591 (39524 , 318975)** | **2.1 (0.5 , 4.4)** | **75.2 (19.5 , 157.2)** | **338416 (89233 , 701103)** | **3.4 (0.9 , 7)** | **75.9 (19.9 , 157.3)** | **1 (-4.6 , 7)** |
| **Brunei Darussalam** | **316 (93 , 633)** | **4.5 (1.4 , 8.7)** | **357.1 (107.4 , 703.2)** | **1068 (324 , 2092)** | **6.7 (2 , 12.7)** | **379 (117.5 , 725.8)** | **6.1 (-11.2 , 30.2)** |
| **Japan** | **127154 (32488 , 265468)** | **2.2 (0.6 , 4.7)** | **73.9 (18.9 , 154.2)** | **243410 (63441 , 506134)** | **3.3 (0.9 , 6.7)** | **70.2 (18.3 , 147.3)** | **-5.1 (-11 , 0.7)** |
| **Singapore** | **3532 (930 , 7291)** | **3.7 (1 , 7.7)** | **161.9 (42.8 , 333.4)** | **7272 (1964 , 15085)** | **4.5 (1.2 , 9.2)** | **92.8 (25.1 , 192.1)** | **-42.7 (-48.5 , -36.4)** |
| **Republic of Korea** | **21589 (5524 , 45665)** | **1.4 (0.4 , 3.1)** | **71.3 (18.4 , 151.6)** | **86666 (22522 , 179160)** | **3.8 (1 , 7.8)** | **95.7 (24.9 , 197.8)** | **34.2 (19.9 , 52.3)** |
| **Western Europe** | **733025 (191793 , 1504937)** | **3.3 (0.9 , 6.9)** | **125.8 (32.9 , 259.7)** | **1293209 (369006 , 2586197)** | **5.2 (1.4 , 10.4)** | **145.2 (41 , 291.3)** | **15.4 (9 , 27.1)** |
| **Andorra** | **56 (13 , 132)** | **2.2 (0.5 , 4.6)** | **99.9 (23.3 , 237.8)** | **189 (47 , 418)** | **3.8 (1 , 7.8)** | **135.4 (33.4 , 300.3)** | **35.4 (-4.8 , 89.9)** |
| **Austria** | **9891 (2540 , 20863)** | **2.2 (0.6 , 4.6)** | **83.2 (21.1 , 176.3)** | **18269 (4967 , 37737)** | **4.1 (1.1 , 8.4)** | **104.6 (28.4 , 217.4)** | **25.8 (14.6 , 42.6)** |
| **Belgium** | **19915 (4885 , 42559)** | **3 (0.7 , 6.5)** | **128 (31.4 , 274.6)** | **30218 (8249 , 62352)** | **4.6 (1.3 , 9.4)** | **135.2 (36.8 , 280)** | **5.6 (-4.1 , 21.1)** |
| **Cyprus** | **1034 (291 , 2069)** | **4.6 (1.3 , 9.2)** | **123.3 (34.8 , 246.8)** | **3049 (844 , 6113)** | **6.2 (1.8 , 12.2)** | **152.9 (42.4 , 306.5)** | **24 (6.7 , 45.9)** |
| **Denmark** | **7193 (1808 , 15355)** | **2.1 (0.5 , 4.6)** | **90.9 (22.8 , 194.1)** | **15123 (4185 , 31357)** | **4.2 (1.1 , 8.6)** | **129.6 (35.7 , 270.4)** | **42.6 (28.6 , 64.7)** |
| **Finland** | **8098 (2153 , 16701)** | **3.4 (0.9 , 7.1)** | **112.6 (29.7 , 232.5)** | **15058 (4271 , 30522)** | **5.4 (1.5 , 10.8)** | **123.2 (34.7 , 251.4)** | **9.4 (-0.6 , 25.5)** |
| **France** | **52922 (13123 , 114011)** | **1.6 (0.4 , 3.4)** | **64.5 (16 , 139.3)** | **109738 (28751 , 231418)** | **2.9 (0.7 , 6.1)** | **83.9 (21.8 , 177.3)** | **30.2 (18.2 , 46.2)** |
| **Germany** | **192100 (50451 , 393966)** | **4.2 (1.1 , 8.6)** | **151 (39.3 , 310.6)** | **336850 (97624 , 672480)** | **6.3 (1.8 , 12.6)** | **181 (50.9 , 362.9)** | **19.8 (8.4 , 37.6)** |
| **Greece** | **15109 (3741 , 32408)** | **2.8 (0.7 , 6.1)** | **96.8 (24 , 207.9)** | **32625 (8736 , 67737)** | **5 (1.3 , 10.2)** | **144.9 (38.1 , 303.7)** | **49.7 (36 , 69.6)** |
| **Iceland** | **205 (53 , 433)** | **2 (0.5 , 4.3)** | **72 (18.5 , 152.6)** | **528 (146 , 1109)** | **3.9 (1 , 8)** | **96.1 (26.3 , 202.2)** | **33.6 (19.2 , 54.1)** |
| **Ireland** | **2750 (692 , 5840)** | **1.6 (0.4 , 3.5)** | **66.1 (16.5 , 140.7)** | **9214 (2551 , 18974)** | **4.3 (1.2 , 8.8)** | **121.8 (33.7 , 251.4)** | **84.4 (65.8 , 115.5)** |
| **Israel** | **4757 (1283 , 9895)** | **2.9 (0.8 , 6)** | **96.8 (26.1 , 201)** | **13973 (3986 , 28257)** | **4.6 (1.3 , 9.2)** | **120.6 (34.2 , 244.4)** | **24.5 (14 , 40.2)** |
| **Italy** | **126002 (33317 , 262197)** | **3.6 (1 , 7.5)** | **140 (36.9 , 291.7)** | **201311 (57060 , 402358)** | **5.6 (1.6 , 11.2)** | **144.5 (41.2 , 290.7)** | **3.2 (-3.5 , 14.4)** |
| **Luxembourg** | **471 (118 , 997)** | **2 (0.5 , 4.2)** | **85.5 (21.4 , 181.5)** | **1698 (485 , 3481)** | **6.2 (1.8 , 12.4)** | **171.5 (48.8 , 352.6)** | **100.6 (72.6 , 151.4)** |
| **Malta** | **609 (164 , 1251)** | **4.2 (1.1 , 8.5)** | **140.6 (38 , 289)** | **1288 (362 , 2644)** | **6.2 (1.8 , 12.4)** | **137.7 (38.2 , 283.7)** | **-2.1 (-13.5 , 14.6)** |
| **Monaco** | **67 (17 , 152)** | **2.4 (0.6 , 5.1)** | **98.3 (24.2 , 221.3)** | **204 (54 , 438)** | **4.7 (1.3 , 9.7)** | **220.3 (57.1 , 478.5)** | **124.2 (71.3 , 204.1)** |
| **Netherlands** | **24760 (6014 , 52678)** | **3 (0.7 , 6.4)** | **123.2 (29.9 , 262.5)** | **43361 (11814 , 91112)** | **3.9 (1 , 8)** | **125.9 (34.2 , 263.9)** | **2.2 (-9.1 , 19.2)** |
| **Norway** | **7531 (2044 , 15469)** | **3.4 (0.9 , 6.9)** | **111.4 (30.1 , 229.8)** | **12081 (3395 , 24569)** | **4.8 (1.4 , 9.8)** | **125.4 (35 , 256.2)** | **12.6 (5.2 , 25.4)** |
| **Portugal** | **15321 (4016 , 31633)** | **3 (0.8 , 6.1)** | **108 (28.5 , 223.2)** | **34480 (9963 , 68873)** | **5.5 (1.6 , 10.9)** | **149 (43 , 298.7)** | **37.9 (25.8 , 57)** |
| **San Marino** | **31 (8 , 68)** | **2.3 (0.6 , 4.9)** | **91.7 (23.5 , 199.1)** | **92 (23 , 230)** | **4.1 (1.1 , 8.4)** | **144.8 (36.4 , 368.6)** | **58 (1.4 , 140.4)** |
| **Spain** | **74601 (19375 , 152237)** | **3.8 (1 , 7.8)** | **133.3 (34.5 , 273.5)** | **138275 (38266 , 283316)** | **5.6 (1.5 , 11.4)** | **150.1 (41.1 , 306.5)** | **12.6 (1.8 , 26.1)** |
| **Sweden** | **11781 (3153 , 24542)** | **2.6 (0.7 , 5.4)** | **78.8 (20.8 , 164.2)** | **21094 (5902 , 42939)** | **4.3 (1.2 , 8.6)** | **100.1 (27.7 , 204.8)** | **27 (17.4 , 43.9)** |
| **Switzerland** | **11032 (2798 , 22929)** | **3.2 (0.8 , 6.7)** | **107.6 (27.2 , 225.4)** | **17076 (4773 , 34949)** | **4.3 (1.2 , 8.8)** | **99.7 (27.7 , 204.7)** | **-7.3 (-17.3 , 8.6)** |
| **United Kingdom** | **146181 (38825 , 300915)** | **4 (1.1 , 8.2)** | **160.4 (42.6 , 331.9)** | **236287 (67904 , 467039)** | **6.3 (1.8 , 12.5)** | **189.5 (53.9 , 376.9)** | **18.2 (9.9 , 34.3)** |
| **Southern Latin America** | **43770 (11304 , 91388)** | **2.1 (0.5 , 4.4)** | **93.7 (24.2 , 195.3)** | **116880 (32392 , 236478)** | **4.1 (1.1 , 8.2)** | **139.7 (38.6 , 283.2)** | **49 (37.4 , 68.6)** |
| **Argentina** | **34185 (8837 , 71266)** | **2.3 (0.6 , 4.9)** | **104.2 (26.9 , 216.9)** | **82766 (22679 , 168260)** | **4.2 (1.1 , 8.5)** | **153.4 (42 , 312.4)** | **47.2 (34.2 , 67.4)** |
| **Chile** | **7210 (1864 , 14920)** | **1.7 (0.4 , 3.4)** | **73.1 (19 , 151.3)** | **26710 (7535 , 53968)** | **3.9 (1.1 , 7.8)** | **110.1 (31 , 222.5)** | **50.6 (35.9 , 75)** |
| **Uruguay** | **2374 (591 , 5034)** | **1.2 (0.3 , 2.6)** | **59.9 (14.9 , 127.3)** | **7398 (1995 , 15363)** | **3.5 (0.9 , 7.2)** | **139.8 (37.4 , 290.7)** | **133.4 (111.1 , 170.4)** |
| **Eastern Europe** | **191090 (47353 , 410634)** | **1.6 (0.4 , 3.4)** | **66.5 (16.5 , 142.3)** | **263707 (69152 , 559496)** | **2.4 (0.6 , 5)** | **75.9 (19.9 , 161.4)** | **14.2 (2.6 , 30.5)** |
| **Belarus** | **8760 (2141 , 19134)** | **1.6 (0.4 , 3.4)** | **66 (16.1 , 143.5)** | **10253 (2485 , 23447)** | **2 (0.5 , 4.4)** | **64.2 (15.5 , 146.8)** | **-2.8 (-25.5 , 27)** |
| **Estonia** | **1849 (449 , 4000)** | **2 (0.5 , 4.3)** | **89.3 (21.7 , 193.2)** | **2607 (658 , 5702)** | **3.2 (0.9 , 6.8)** | **101.7 (25.5 , 225)** | **13.9 (-12.2 , 47.7)** |
| **Latvia** | **2734 (679 , 5901)** | **1.8 (0.5 , 3.9)** | **75.7 (18.8 , 163.5)** | **3922 (1006 , 8555)** | **3.2 (0.9 , 6.7)** | **102.7 (26.1 , 225.3)** | **35.7 (11.5 , 68.8)** |
| **Lithuania** | **3154 (774 , 6857)** | **1.7 (0.4 , 3.6)** | **69.5 (17.1 , 150.8)** | **4263 (1041 , 9350)** | **2.4 (0.6 , 5.2)** | **77.7 (18.9 , 170.8)** | **11.8 (-10.8 , 39.5)** |
| **Republic of Moldova** | **4071 (1011 , 8654)** | **2.1 (0.5 , 4.5)** | **87.6 (21.7 , 185.4)** | **5496 (1420 , 11631)** | **3.3 (0.9 , 6.7)** | **93.3 (24.3 , 197.5)** | **6.6 (-8.7 , 25.7)** |
| **Russian Federation** | **112967 (27629 , 243941)** | **1.5 (0.4 , 3.2)** | **60.7 (14.8 , 130.5)** | **174869 (45080 , 372029)** | **2.3 (0.6 , 5)** | **73.3 (18.9 , 156.3)** | **20.9 (6.1 , 42.4)** |
| **Ukraine** | **57555 (14220 , 124246)** | **1.7 (0.4 , 3.8)** | **78.9 (19.5 , 170.1)** | **62297 (15906 , 135775)** | **2.3 (0.6 , 4.9)** | **83.3 (21.2 , 181.7)** | **5.5 (-12 , 28.9)** |
| **Central Europe** | **201123 (51524 , 423002)** | **3 (0.8 , 6.2)** | **133.4 (34.3 , 280.2)** | **421066 (115565 , 878373)** | **5.4 (1.5 , 10.9)** | **196.5 (53.6 , 413.1)** | **47.3 (28.6 , 71.4)** |
| **Albania** | **868 (199 , 1955)** | **1.1 (0.3 , 2.5)** | **42.5 (9.9 , 95.6)** | **2765 (650 , 6677)** | **2.5 (0.6 , 5.3)** | **63.1 (14.9 , 152.2)** | **48.6 (8.3 , 100.7)** |
| **Bosnia and Herzegovina** | **4361 (1087 , 9379)** | **2.6 (0.7 , 5.6)** | **103.9 (26 , 221.3)** | **14945 (4091 , 32426)** | **6.8 (1.9 , 13.6)** | **242.3 (66 , 527.1)** | **133.3 (79.4 , 210.5)** |
| **Bulgaria** | **12434 (3223 , 26294)** | **2.6 (0.7 , 5.5)** | **95.8 (24.9 , 203)** | **24712 (6530 , 55687)** | **4.7 (1.3 , 9.7)** | **176.8 (46.4 , 399.3)** | **84.5 (45.4 , 137.4)** |
| **Croatia** | **9543 (2382 , 19984)** | **3.2 (0.8 , 6.7)** | **146.1 (36.7 , 304.6)** | **16571 (4460 , 36905)** | **5.6 (1.5 , 11.4)** | **189 (50.5 , 423.6)** | **29.4 (1.8 , 68.2)** |
| **Czechia** | **31008 (8146 , 64213)** | **4.4 (1.2 , 9.2)** | **223.2 (58.4 , 463.1)** | **51857 (15215 , 109858)** | **8 (2.3 , 15.4)** | **243.7 (70.7 , 518.6)** | **9.2 (-12.6 , 41.3)** |
| **Hungary** | **24516 (6256 , 52099)** | **3.1 (0.8 , 6.6)** | **165.2 (42.3 , 351.5)** | **45848 (12524 , 98867)** | **5.9 (1.6 , 12)** | **242.3 (66.2 , 526.5)** | **46.6 (19.6 , 86.1)** |
| **Montenegro** | **899 (222 , 1919)** | **3.4 (0.8 , 7.2)** | **142.6 (35.2 , 303.9)** | **2531 (662 , 5447)** | **6.3 (1.7 , 12.8)** | **250.7 (65.5 , 538.9)** | **75.9 (44.4 , 117.8)** |
| **North Macedonia** | **2228 (575 , 4669)** | **2.7 (0.7 , 5.6)** | **115.3 (29.9 , 240)** | **8612 (2358 , 19049)** | **6.3 (1.8 , 12.6)** | **256.3 (70.2 , 564.8)** | **122.2 (75.6 , 191.9)** |
| **Poland** | **68679 (17517 , 144993)** | **3.1 (0.8 , 6.6)** | **154.7 (39.4 , 326.2)** | **146739 (38597 , 313868)** | **5.5 (1.5 , 11.1)** | **209.1 (54.7 , 448.3)** | **35.2 (14.4 , 63.2)** |
| **Romania** | **18795 (4655 , 40737)** | **1.8 (0.5 , 3.9)** | **64.4 (16 , 139.4)** | **44967 (11386 , 97511)** | **3.5 (0.9 , 7.4)** | **125.2 (31.6 , 274.2)** | **94.3 (57.4 , 145.2)** |
| **Serbia** | **17186 (4317 , 36808)** | **3.3 (0.8 , 6.9)** | **144.9 (36.9 , 308.7)** | **42283 (11413 , 92401)** | **6.4 (1.8 , 13)** | **262 (70 , 575.4)** | **80.9 (42.1 , 140.7)** |
| **Slovakia** | **7489 (1900 , 15898)** | **2.7 (0.7 , 5.7)** | **123.5 (31.2 , 261.6)** | **13570 (3549 , 30295)** | **4.2 (1.1 , 8.5)** | **144 (37.5 , 322.6)** | **16.6 (-10.5 , 53.6)** |
| **Slovenia** | **3118 (771 , 7117)** | **3 (0.8 , 6.3)** | **126.5 (31.3 , 288.3)** | **5665 (1452 , 12466)** | **4.4 (1.2 , 9)** | **133.1 (33.8 , 293.9)** | **5.2 (-27.8 , 48.4)** |
| **Central Asia** | **30222 (7357 , 65479)** | **1.4 (0.3 , 3)** | **62.1 (15.2 , 134)** | **75461 (19933 , 156357)** | **2.8 (0.8 , 5.7)** | **98.8 (26.4 , 203.1)** | **59 (41.3 , 86.6)** |
| **Armenia** | **2762 (675 , 5924)** | **2.1 (0.5 , 4.5)** | **95.8 (23.8 , 204)** | **6382 (1662 , 13753)** | **4.4 (1.1 , 9.1)** | **149.8 (39 , 323.8)** | **56.3 (29.8 , 93.9)** |
| **Azerbaijan** | **2939 (698 , 6422)** | **1.3 (0.3 , 2.7)** | **55.2 (13.2 , 120.4)** | **10903 (2802 , 23511)** | **3 (0.8 , 6.2)** | **106.8 (28.1 , 229.2)** | **93.4 (58.3 , 147)** |
| **Georgia** | **4809 (1155 , 10485)** | **2 (0.5 , 4.2)** | **74.6 (18 , 161.6)** | **10109 (2623 , 21612)** | **4.7 (1.2 , 9.7)** | **174.4 (45.2 , 373.9)** | **133.8 (91.2 , 197.1)** |
| **Kazakhstan** | **12442 (3075 , 27179)** | **1.8 (0.5 , 3.9)** | **94.5 (23.6 , 205.5)** | **22126 (5872 , 46283)** | **3.7 (1 , 7.6)** | **123.5 (32.8 , 259.3)** | **30.6 (10.2 , 61.2)** |
| **Kyrgyzstan** | **1458 (352 , 3204)** | **1.1 (0.3 , 2.4)** | **47.3 (11.5 , 103.2)** | **2019 (512 , 4398)** | **1.6 (0.4 , 3.4)** | **43.1 (11 , 93.2)** | **-8.9 (-22 , 9)** |
| **Mongolia** | **385 (89 , 899)** | **0.5 (0.1 , 1)** | **37 (8.6 , 86.4)** | **1265 (304 , 2909)** | **0.7 (0.2 , 1.6)** | **56 (13.6 , 126.6)** | **51.2 (16.8 , 103.5)** |
| **Tajikistan** | **1159 (287 , 2519)** | **0.9 (0.2 , 2)** | **40.4 (10 , 87.9)** | **3714 (953 , 8098)** | **2.1 (0.6 , 4.2)** | **73.3 (19.6 , 157.9)** | **81.4 (44.8 , 141.3)** |
| **Turkmenistan** | **701 (170 , 1525)** | **0.8 (0.2 , 1.8)** | **34.4 (8.4 , 74.3)** | **2118 (548 , 4692)** | **1.7 (0.5 , 3.6)** | **50.5 (13.1 , 111.1)** | **46.9 (17.7 , 88)** |
| **Uzbekistan** | **3566 (872 , 7772)** | **0.8 (0.2 , 1.8)** | **30.3 (7.4 , 65.9)** | **16826 (4352 , 35837)** | **2.2 (0.6 , 4.5)** | **75.9 (19.7 , 160.3)** | **150.3 (102.4 , 223.1)** |
| **Central Latin America** | **67307 (18800 , 135265)** | **2.2 (0.6 , 4.5)** | **83.1 (23.3 , 165.2)** | **236568 (67139 , 491403)** | **3.9 (1.1 , 7.6)** | **100.2 (28.5 , 207.5)** | **20.5 (4 , 41)** |
| **Colombia** | **14382 (3860 , 29342)** | **2 (0.6 , 4.2)** | **85 (23 , 172.6)** | **41957 (11225 , 94516)** | **3.4 (1 , 6.8)** | **79.7 (21.3 , 179.5)** | **-6.3 (-29.1 , 20.8)** |
| **Costa Rica** | **1091 (294 , 2211)** | **1.7 (0.5 , 3.4)** | **64.3 (17.5 , 130.4)** | **5282 (1476 , 11783)** | **3.6 (1 , 7.3)** | **103.1 (28.9 , 229.7)** | **60.3 (24.6 , 111.2)** |
| **El Salvador** | **867 (229 , 1810)** | **1 (0.3 , 2.2)** | **29.7 (7.9 , 61.8)** | **4710 (1288 , 10698)** | **3.2 (0.9 , 6.5)** | **80.6 (22 , 183.3)** | **171.4 (100 , 265)** |
| **Guatemala** | **1208 (318 , 2557)** | **0.9 (0.2 , 1.9)** | **34.2 (9.1 , 71.8)** | **9084 (2568 , 19963)** | **2.6 (0.7 , 5)** | **82.7 (23.5 , 180.4)** | **141.6 (87.3 , 227.1)** |
| **Honduras** | **1084 (291 , 2349)** | **1.3 (0.4 , 2.8)** | **53.1 (14.2 , 114.5)** | **7479 (2001 , 16838)** | **3.5 (1 , 7.5)** | **123.9 (33.6 , 276.8)** | **133.3 (75.6 , 213.9)** |
| **Mexico** | **40361 (11627 , 79434)** | **2.7 (0.8 , 5.4)** | **97.3 (28.1 , 189.8)** | **124924 (35442 , 253623)** | **4.3 (1.3 , 8.5)** | **106.2 (30.2 , 214.9)** | **9.1 (-6.8 , 26.6)** |
| **Nicaragua** | **617 (164 , 1278)** | **1.2 (0.3 , 2.5)** | **41.8 (11.2 , 86.6)** | **3717 (1029 , 7749)** | **3.1 (0.9 , 6.2)** | **86.2 (24 , 177.9)** | **106 (64 , 164.8)** |
| **Panama** | **894 (239 , 1852)** | **1.9 (0.5 , 3.9)** | **61.2 (16.3 , 126.5)** | **3715 (1003 , 8202)** | **3.7 (1.1 , 7.5)** | **90.2 (24.4 , 198.9)** | **47.5 (12.8 , 95.6)** |
| **Venezuela (Bolivarian Republic of)** | **6804 (1807 , 14089)** | **1.8 (0.5 , 3.7)** | **71.9 (19.3 , 148.5)** | **35698 (9755 , 81243)** | **3.9 (1.1 , 7.8)** | **121.3 (33.1 , 276.2)** | **68.6 (26.1 , 126.1)** |
| **Andean Latin America** | **6610 (1693 , 13986)** | **0.8 (0.2 , 1.6)** | **33.5 (8.6 , 70.8)** | **32792 (8962 , 70558)** | **2 (0.5 , 4.1)** | **59.5 (16.2 , 127.7)** | **77.7 (45.4 , 121.7)** |
| **Bolivia (Plurinational State of)** | **1474 (370 , 3347)** | **0.9 (0.2 , 1.8)** | **47.2 (11.9 , 106.5)** | **7188 (1791 , 16484)** | **2 (0.5 , 4.2)** | **82.9 (20.9 , 188.9)** | **75.4 (36.8 , 130.3)** |
| **Ecuador** | **1573 (411 , 3319)** | **0.9 (0.2 , 1.8)** | **30.7 (8 , 64.6)** | **11003 (3008 , 23563)** | **2.5 (0.7 , 4.9)** | **74.1 (20.3 , 158.2)** | **141.5 (90.3 , 220.3)** |
| **Peru** | **3562 (888 , 7739)** | **0.7 (0.2 , 1.6)** | **31 (7.7 , 67.2)** | **14601 (3789 , 34378)** | **1.8 (0.5 , 3.6)** | **46.2 (12 , 108.8)** | **49.1 (9.9 , 109.4)** |
| **Caribbean** | **24718 (6633 , 50697)** | **2.5 (0.7 , 5.2)** | **95.5 (25.7 , 195.7)** | **72360 (20252 , 150189)** | **4.2 (1.2 , 8.3)** | **139.5 (39 , 289.3)** | **46.1 (27.1 , 73.5)** |
| **Antigua and Barbuda** | **41 (11 , 84)** | **2.3 (0.7 , 4.8)** | **77.4 (21.4 , 159.5)** | **147 (42 , 308)** | **4.5 (1.3 , 9)** | **144.6 (41.7 , 302)** | **86.8 (60 , 127.9)** |
| **Barbados** | **270 (72 , 547)** | **2.6 (0.7 , 5.3)** | **94.2 (24.7 , 193.1)** | **831 (230 , 1782)** | **4.6 (1.3 , 9.2)** | **167.4 (46.2 , 359.8)** | **77.7 (45.7 , 118.7)** |
| **Belize** | **33 (9 , 69)** | **1.2 (0.3 , 2.5)** | **36 (9.5 , 75.5)** | **235 (64 , 495)** | **2.7 (0.8 , 5.5)** | **84.4 (23.1 , 176.8)** | **134.5 (99 , 186.4)** |
| **Bermuda** | **81 (21 , 170)** | **2.7 (0.7 , 5.6)** | **130.2 (33.7 , 273.4)** | **159 (43 , 346)** | **4.4 (1.2 , 9)** | **120.5 (32.7 , 261.9)** | **-7.4 (-22.4 , 17)** |
| **Bahamas** | **169 (44 , 356)** | **2.2 (0.6 , 4.5)** | **109.2 (29 , 227.6)** | **622 (172 , 1336)** | **3.9 (1.1 , 8)** | **155.5 (43.5 , 331.7)** | **42.4 (15.3 , 79.8)** |
| **Cuba** | **12428 (3229 , 25782)** | **3.3 (0.9 , 7)** | **120.8 (31.4 , 250.8)** | **31782 (8539 , 67523)** | **5.2 (1.4 , 10.6)** | **167.2 (44.8 , 354.6)** | **38.4 (11.7 , 75.6)** |
| **Dominica** | **72 (19 , 149)** | **2.2 (0.6 , 4.5)** | **101 (26.7 , 211.2)** | **165 (47 , 348)** | **4.2 (1.2 , 8)** | **183.3 (51.4 , 386)** | **81.4 (45.1 , 138.2)** |
| **Dominican Republic** | **865 (215 , 1868)** | **0.8 (0.2 , 1.7)** | **23.6 (5.9 , 50.9)** | **5747 (1441 , 13313)** | **2 (0.5 , 4.2)** | **62 (15.7 , 143.2)** | **162.4 (84.8 , 264.6)** |
| **Grenada** | **72 (20 , 147)** | **2.2 (0.6 , 4.5)** | **102.3 (27.5 , 212.2)** | **219 (65 , 437)** | **4.6 (1.3 , 9.1)** | **194 (57.6 , 384.6)** | **89.6 (63.9 , 129.1)** |
| **Guyana** | **346 (93 , 725)** | **2.2 (0.6 , 4.5)** | **91 (24.7 , 188.6)** | **1054 (298 , 2321)** | **4.6 (1.4 , 9.1)** | **161.1 (46.2 , 350.9)** | **76.9 (34.3 , 135.9)** |
| **Haiti** | **2853 (704 , 6331)** | **1.4 (0.3 , 3)** | **84.5 (21.5 , 184.3)** | **9224 (2430 , 21245)** | **2.7 (0.7 , 5.5)** | **127 (33.7 , 289.1)** | **50.2 (10.9 , 107.4)** |
| **Jamaica** | **1279 (339 , 2612)** | **2.5 (0.7 , 5.1)** | **72.9 (19.2 , 149.8)** | **5069 (1423 , 10960)** | **4.8 (1.4 , 9.6)** | **171.9 (48.3 , 371.4)** | **135.6 (88.1 , 206.5)** |
| **Puerto Rico** | **3759 (1033 , 7549)** | **3.5 (1 , 7)** | **103.3 (28.2 , 207.6)** | **9524 (2731 , 20693)** | **6.8 (2 , 13.2)** | **138.7 (39.2 , 302.8)** | **34.3 (3 , 76)** |
| **Saint Kitts and Nevis** | **52 (14 , 105)** | **2.8 (0.8 , 5.7)** | **140.4 (38.7 , 285.2)** | **113 (32 , 237)** | **4.4 (1.3 , 8.8)** | **169 (48.1 , 350)** | **20.4 (-0.6 , 50.5)** |
| **Saint Lucia** | **115 (32 , 228)** | **2.9 (0.8 , 5.8)** | **133.3 (37.6 , 262.7)** | **358 (105 , 735)** | **4.9 (1.5 , 9.5)** | **165 (48.2 , 338.2)** | **23.8 (2.9 , 51.6)** |
| **Saint Vincent and the Grenadines** | **71 (20 , 145)** | **2.3 (0.7 , 4.7)** | **99.8 (28.2 , 203.7)** | **216 (64 , 443)** | **4.2 (1.3 , 8.3)** | **158 (46.6 , 324.6)** | **58.3 (35.3 , 92.2)** |
| **Suriname** | **204 (55 , 419)** | **2.4 (0.6 , 4.9)** | **78.9 (21.4 , 162)** | **1030 (294 , 2150)** | **5.4 (1.6 , 10.5)** | **169.2 (48.4 , 350.5)** | **114.6 (79.1 , 170.3)** |
| **Trinidad and Tobago** | **1095 (311 , 2170)** | **3.5 (1 , 6.9)** | **131.2 (37.2 , 260.2)** | **3000 (835 , 6650)** | **6.1 (1.8 , 11.8)** | **158.3 (44 , 351.3)** | **20.7 (-10.7 , 65)** |
| **United States Virgin Islands** | **90 (24 , 192)** | **2.6 (0.7 , 5.4)** | **106.5 (28.9 , 227.6)** | **416 (117 , 854)** | **5.6 (1.6 , 11.1)** | **219.3 (61.2 , 451.8)** | **106 (67.7 , 159.8)** |
| **Tropical Latin America** | **70014 (18620 , 143727)** | **1.9 (0.5 , 3.9)** | **77.2 (20.7 , 157.6)** | **203917 (56014 , 420429)** | **2.9 (0.8 , 5.9)** | **84 (23.2 , 172.7)** | **8.7 (2.4 , 17.4)** |
| **Brazil** | **69291 (18448 , 142358)** | **1.9 (0.5 , 4)** | **78.4 (21 , 159.9)** | **199117 (54797 , 409767)** | **2.9 (0.8 , 5.9)** | **83.9 (23.2 , 172.5)** | **7 (0.6 , 15.6)** |
| **Paraguay** | **723 (184 , 1556)** | **1.2 (0.3 , 2.4)** | **33.2 (8.5 , 71.1)** | **4800 (1235 , 10841)** | **2.9 (0.8 , 6)** | **87.7 (22.6 , 197.6)** | **164.3 (94.7 , 254.7)** |
| **East Asia** | **623966 (157519 , 1351193)** | **1.3 (0.3 , 2.9)** | **69.9 (17.8 , 150.4)** | **2034530 (517721 , 4451841)** | **2.9 (0.7 , 6.2)** | **95.7 (24.4 , 209.1)** | **37 (12.6 , 69.1)** |
| **China** | **603435 (152503 , 1309632)** | **1.3 (0.3 , 2.9)** | **70.1 (17.9 , 151.3)** | **1955214 (496063 , 4292456)** | **2.9 (0.7 , 6.2)** | **95.2 (24.1 , 209.6)** | **35.7 (10.7 , 69.2)** |
| **Democratic People's Republic of Korea** | **8506 (2064 , 19816)** | **1.1 (0.3 , 2.4)** | **50.8 (12.6 , 115.8)** | **26140 (6473 , 58585)** | **2.3 (0.6 , 5)** | **79.7 (19.7 , 177.2)** | **57.1 (20.6 , 102.4)** |
| **Taiwan (Province of China)** | **12025 (3183 , 25085)** | **2 (0.5 , 4.1)** | **74.3 (19.6 , 154.7)** | **53177 (13917 , 115567)** | **4 (1.1 , 8.1)** | **133.3 (34.9 , 289.5)** | **79.4 (40.5 , 136)** |
| **Southeast Asia** | **137389 (34595 , 293754)** | **1.4 (0.4 , 3)** | **54.3 (13.8 , 116)** | **581360 (154813 , 1235009)** | **3.1 (0.8 , 6.2)** | **96.7 (26.2 , 204.4)** | **78 (52.7 , 109.7)** |
| **Cambodia** | **1824 (433 , 4150)** | **0.8 (0.2 , 1.8)** | **41.6 (9.9 , 94.3)** | **13198 (3429 , 28570)** | **3.1 (0.8 , 6.3)** | **113.3 (30 , 241.6)** | **172.6 (112.1 , 254.6)** |
| **Indonesia** | **39286 (9623 , 85468)** | **1.2 (0.3 , 2.5)** | **39.5 (9.9 , 86.2)** | **191611 (48236 , 425477)** | **2.8 (0.7 , 5.9)** | **88.2 (22.5 , 196.4)** | **123.5 (79.5 , 171.1)** |
| **Lao People's Democratic Republic** | **1559 (372 , 3635)** | **1.4 (0.3 , 3.2)** | **74 (17.7 , 171.7)** | **5243 (1354 , 11742)** | **3.4 (0.9 , 7)** | **122.5 (32 , 270.5)** | **65.5 (24.2 , 121.9)** |
| **Malaysia** | **7355 (1946 , 15367)** | **2.1 (0.6 , 4.4)** | **83.2 (22 , 173.6)** | **36025 (9642 , 79438)** | **4.2 (1.2 , 8.5)** | **138.5 (37.6 , 302.5)** | **66.6 (29.1 , 117.8)** |
| **Maldives** | **41 (10 , 96)** | **1.2 (0.3 , 2.6)** | **50 (12.9 , 112.6)** | **162 (44 , 341)** | **2.7 (0.8 , 5.6)** | **60 (16.2 , 124.9)** | **20 (-7.4 , 69)** |
| **Mauritius** | **555 (147 , 1133)** | **2.8 (0.8 , 5.7)** | **75.7 (20.2 , 153.7)** | **2822 (835 , 5888)** | **7.2 (2.2 , 13.9)** | **157.7 (46.8 , 327.8)** | **108.3 (65.2 , 173.1)** |
| **Myanmar** | **19882 (4701 , 47654)** | **1.7 (0.4 , 3.7)** | **82.9 (20 , 196.1)** | **51471 (13534 , 114588)** | **3.4 (0.9 , 6.9)** | **112.8 (29.8 , 250.5)** | **36 (2 , 92.2)** |
| **Philippines** | **20828 (5245 , 44225)** | **1.6 (0.4 , 3.4)** | **73.1 (18.6 , 153.2)** | **67766 (17581 , 149751)** | **2.6 (0.7 , 5.4)** | **87 (22.9 , 190.9)** | **19.1 (-4.5 , 51.6)** |
| **Sri Lanka** | **3555 (940 , 7432)** | **1.4 (0.4 , 2.8)** | **34.4 (9.3 , 71.2)** | **22850 (6542 , 51232)** | **4.7 (1.4 , 9.2)** | **88.3 (25.4 , 197)** | **156.9 (89.6 , 258.5)** |
| **Seychelles** | **50 (13 , 105)** | **2.1 (0.6 , 4.4)** | **88 (23.4 , 185.4)** | **241 (70 , 481)** | **5 (1.5 , 9.7)** | **223.3 (65.8 , 441.6)** | **153.7 (115.8 , 215.1)** |
| **Thailand** | **22910 (5758 , 49243)** | **1.5 (0.4 , 3.2)** | **65.7 (16.7 , 141.3)** | **85843 (21866 , 198558)** | **2.9 (0.8 , 6.1)** | **83.9 (21.4 , 194.4)** | **27.8 (-4.7 , 77.9)** |
| **Timor-Leste** | **90 (22 , 205)** | **0.8 (0.2 , 1.9)** | **34.5 (8.4 , 78.6)** | **702 (181 , 1573)** | **3 (0.8 , 6.4)** | **86.6 (22.5 , 193.4)** | **151.2 (79.9 , 247.3)** |
| **Viet Nam** | **19271 (4693 , 43231)** | **1.5 (0.4 , 3.2)** | **48.1 (11.7 , 107.4)** | **102662 (26215 , 222941)** | **3.2 (0.9 , 6.7)** | **113.4 (29.1 , 243.7)** | **135.7 (79.6 , 211.2)** |
| **Oceania** | **3055 (760 , 6770)** | **2.5 (0.6 , 5.4)** | **96.6 (24.5 , 212.7)** | **13829 (3717 , 30700)** | **4.6 (1.3 , 9.4)** | **177.4 (48 , 384.6)** | **83.6 (50 , 131.4)** |
| **American Samoa** | **48 (13 , 98)** | **5.2 (1.5 , 10.3)** | **212.4 (60 , 428.3)** | **169 (52 , 333)** | **9.2 (2.9 , 17.2)** | **342.3 (106.6 , 674.3)** | **61.1 (30.3 , 105.3)** |
| **Cook Islands** | **21 (5 , 44)** | **4.1 (1.1 , 8.4)** | **163.8 (41.8 , 345.6)** | **57 (16 , 119)** | **7.9 (2.2 , 15.3)** | **229.6 (63.8 , 478.5)** | **40.2 (10.3 , 85.7)** |
| **Micronesia (Federated States of)** | **52 (13 , 118)** | **2.2 (0.6 , 4.5)** | **109.7 (27.3 , 245.3)** | **202 (50 , 489)** | **5.7 (1.6 , 11.9)** | **268.7 (70.2 , 632.3)** | **145 (69.2 , 264.6)** |
| **Fiji** | **432 (116 , 928)** | **2.9 (0.8 , 6.1)** | **115.6 (32.1 , 244)** | **1636 (482 , 3487)** | **6.6 (2 , 12.6)** | **211.2 (63.1 , 442.2)** | **82.7 (36.2 , 162.7)** |
| **Guam** | **84 (21 , 180)** | **3.2 (0.8 , 6.7)** | **115.6 (29.6 , 247)** | **284 (77 , 616)** | **5.3 (1.4 , 10.7)** | **148.2 (40.4 , 321.6)** | **28.3 (3.7 , 62.9)** |
| **Kiribati** | **48 (12 , 107)** | **1.8 (0.5 , 3.8)** | **120.6 (31.3 , 263.5)** | **150 (38 , 331)** | **3.4 (0.9 , 6.9)** | **199 (51.6 , 435.3)** | **65 (22.5 , 128.2)** |
| **Marshall Islands** | **31 (8 , 69)** | **3.6 (1 , 7.6)** | **183.1 (47.9 , 410)** | **132 (35 , 301)** | **7.3 (2.2 , 14.6)** | **344.3 (95.3 , 772.9)** | **88 (42.7 , 155.5)** |
| **Nauru** | **6 (1 , 14)** | **2.2 (0.6 , 4.8)** | **155 (37.8 , 358)** | **13 (3 , 29)** | **4.4 (1.2 , 9.2)** | **276 (72 , 616.2)** | **78 (40.1 , 137.9)** |
| **Niue** | **4 (1 , 7)** | **4.7 (1.3 , 9.5)** | **163.4 (45.9 , 338.9)** | **6 (2 , 14)** | **9.1 (2.7 , 17.5)** | **297.2 (87.4 , 623.3)** | **81.9 (36.6 , 145.3)** |
| **Northern Mariana Islands** | **31 (7 , 69)** | **2.8 (0.7 , 5.9)** | **179.5 (43.2 , 396)** | **140 (38 , 293)** | **6.2 (1.7 , 12.6)** | **258.8 (70.2 , 531.2)** | **44.2 (20.2 , 81.2)** |
| **Palau** | **18 (5 , 40)** | **3.3 (0.9 , 6.8)** | **182.1 (47.7 , 401.3)** | **71 (20 , 149)** | **6.8 (2 , 13.4)** | **316.8 (90.3 , 659.4)** | **74 (28.6 , 138.6)** |
| **Papua New Guinea** | **1779 (419 , 4113)** | **2.5 (0.6 , 5.4)** | **87.5 (20.9 , 203.3)** | **8622 (2193 , 20065)** | **4.3 (1.1 , 9)** | **158.9 (40.8 , 358.5)** | **81.5 (38.1 , 144.1)** |
| **Samoa** | **78 (21 , 169)** | **2.5 (0.7 , 5.1)** | **88.3 (24.2 , 191.1)** | **223 (64 , 493)** | **4.9 (1.4 , 9.9)** | **148.3 (43 , 324.1)** | **67.9 (30.2 , 126.1)** |
| **Solomon Islands** | **128 (27 , 321)** | **1.5 (0.4 , 3.4)** | **86.6 (18.1 , 216.8)** | **997 (242 , 2281)** | **4.1 (1.1 , 8.7)** | **270.9 (66.7 , 612.7)** | **212.7 (126.5 , 363.8)** |
| **Tokelau** | **1 (0 , 3)** | **3.1 (0.8 , 6.4)** | **104.8 (26 , 227.5)** | **3 (1 , 6)** | **6.1 (1.8 , 12.1)** | **192.4 (52.6 , 424.3)** | **83.5 (39.8 , 147.6)** |
| **Tonga** | **67 (18 , 146)** | **2.7 (0.7 , 5.7)** | **118.8 (31.4 , 258.8)** | **155 (42 , 332)** | **4.6 (1.3 , 9.4)** | **195.5 (53.2 , 418.3)** | **64.6 (26.8 , 119.4)** |
| **Tuvalu** | **8 (2 , 18)** | **2.6 (0.7 , 5.5)** | **115 (29.3 , 254.4)** | **22 (6 , 49)** | **5.8 (1.7 , 11.6)** | **211.6 (58.1 , 466.7)** | **84 (36.8 , 156)** |
| **Vanuatu** | **49 (11 , 120)** | **2 (0.5 , 4.5)** | **75.3 (17.1 , 179.8)** | **294 (75 , 667)** | **4.2 (1.2 , 8.6)** | **164.5 (42.3 , 369.2)** | **118.5 (59.5 , 235.1)** |
| **North Africa and Middle East** | **96924 (24858 , 209667)** | **1.6 (0.4 , 3.4)** | **56.3 (14.6 , 121.1)** | **462151 (127349 , 959468)** | **3.8 (1.1 , 7.6)** | **107 (29.8 , 220.8)** | **90.1 (64.4 , 127.8)** |
| **Afghanistan** | **4290 (1018 , 10173)** | **1.1 (0.3 , 2.5)** | **58.6 (14.2 , 137.1)** | **13645 (3606 , 31320)** | **1.7 (0.5 , 3.6)** | **106.7 (29 , 238.8)** | **82 (36.6 , 143.6)** |
| **Algeria** | **5510 (1427 , 12280)** | **1.5 (0.4 , 3.3)** | **46.5 (12.1 , 103.2)** | **27598 (7625 , 58330)** | **4.1 (1.2 , 8.2)** | **82.8 (23.3 , 173.5)** | **78 (35.6 , 143.8)** |
| **Bahrain** | **376 (104 , 777)** | **4.8 (1.3 , 9.5)** | **248.5 (69.9 , 500.1)** | **2023 (601 , 4073)** | **8.2 (2.6 , 15.3)** | **240.8 (74.5 , 472.1)** | **-3.1 (-26.2 , 34.7)** |
| **Egypt** | **7949 (2090 , 16658)** | **1 (0.3 , 2)** | **26.4 (7 , 55.3)** | **54923 (14509 , 127141)** | **3 (0.8 , 6.2)** | **81.8 (21.8 , 188.4)** | **209.3 (125.9 , 331.7)** |
| **Iran (Islamic Republic of)** | **9532 (2453 , 20653)** | **1 (0.3 , 2.2)** | **37 (9.6 , 79.7)** | **58946 (16763 , 119374)** | **3.3 (0.9 , 6.7)** | **82 (23.5 , 165)** | **121.3 (93.3 , 169.7)** |
| **Iraq** | **6471 (1697 , 14402)** | **2.3 (0.6 , 5)** | **82.9 (21.3 , 184.3)** | **35527 (9893 , 77129)** | **4.6 (1.3 , 9.1)** | **152.7 (43.7 , 324.4)** | **84.2 (36.9 , 148.5)** |
| **Jordan** | **1125 (298 , 2396)** | **2.3 (0.6 , 4.8)** | **85.2 (22.9 , 178.7)** | **7896 (2164 , 16738)** | **4.6 (1.3 , 9.2)** | **123.4 (34.2 , 256.5)** | **44.8 (17.1 , 86.7)** |
| **Kuwait** | **494 (137 , 1005)** | **2.7 (0.7 , 5.5)** | **86 (24.6 , 172.2)** | **2746 (799 , 5615)** | **5.8 (1.7 , 11.3)** | **116.7 (34.4 , 234.9)** | **35.7 (14.6 , 67.3)** |
| **Lebanon** | **2457 (629 , 5317)** | **2.9 (0.8 , 6.1)** | **108.4 (28 , 231.6)** | **11536 (3217 , 23874)** | **6.3 (1.8 , 12.4)** | **222.1 (62 , 459.5)** | **104.9 (61.9 , 187.9)** |
| **Libya** | **1606 (408 , 3568)** | **2.5 (0.6 , 5.3)** | **88.1 (22.6 , 195.9)** | **8838 (2407 , 18898)** | **5.5 (1.6 , 11)** | **175 (48 , 369.2)** | **98.5 (50.3 , 184.8)** |
| **Morocco** | **6717 (1656 , 14590)** | **1.9 (0.5 , 4.1)** | **47.6 (11.7 , 104.2)** | **38079 (9788 , 83922)** | **4.7 (1.3 , 9.8)** | **115.5 (30.4 , 251.7)** | **142.9 (83.3 , 224.5)** |
| **Palestine** | **835 (206 , 1853)** | **2.4 (0.6 , 5.1)** | **97.8 (24.1 , 216.9)** | **4688 (1315 , 9671)** | **5.3 (1.5 , 10.5)** | **205 (58 , 415.5)** | **109.7 (59.7 , 197)** |
| **Oman** | **293 (74 , 667)** | **1.5 (0.4 , 3.2)** | **46.2 (11.8 , 103.7)** | **1500 (420 , 3094)** | **3.3 (1 , 6.7)** | **99.3 (29 , 201.5)** | **115 (67.5 , 200)** |
| **Qatar** | **150 (42 , 314)** | **3.1 (0.9 , 6.3)** | **171.7 (49.9 , 346.8)** | **1974 (583 , 4071)** | **6.9 (2.2 , 13.1)** | **270.4 (84.1 , 525.9)** | **57.4 (18.9 , 120.9)** |
| **Saudi Arabia** | **2783 (731 , 6353)** | **2 (0.5 , 4.2)** | **49.1 (13 , 110.8)** | **19011 (5249 , 40435)** | **4.1 (1.2 , 8.3)** | **102.4 (29.3 , 211.7)** | **108.7 (55.6 , 191.5)** |
| **Sudan** | **3008 (746 , 7202)** | **1 (0.2 , 2.3)** | **32.4 (8 , 78.5)** | **13893 (3754 , 31710)** | **2.5 (0.7 , 5.1)** | **75.3 (20.9 , 170.3)** | **132 (75.2 , 225.5)** |
| **Syrian Arab Republic** | **2016 (517 , 4506)** | **1.2 (0.3 , 2.6)** | **38.5 (9.9 , 86.1)** | **9011 (2326 , 19813)** | **3.5 (1 , 7)** | **71.8 (18.9 , 156.8)** | **86.6 (32.9 , 166)** |
| **Tunisia** | **3610 (865 , 8022)** | **3 (0.7 , 6.5)** | **72.2 (17.5 , 159.2)** | **17163 (4322 , 39392)** | **6.6 (1.8 , 13.2)** | **134.6 (34.3 , 310)** | **86.5 (30.8 , 175.1)** |
| **Turkey** | **35783 (8481 , 80328)** | **2.1 (0.5 , 4.5)** | **98.8 (23.5 , 219.7)** | **117239 (29332 , 256769)** | **4.4 (1.1 , 9.2)** | **132.3 (33.2 , 289.2)** | **33.9 (1.2 , 80.4)** |
| **United Arab Emirates** | **538 (146 , 1148)** | **2.1 (0.6 , 4.4)** | **166.5 (46.8 , 348.9)** | **8432 (2275 , 18166)** | **4 (1.2 , 8.2)** | **247.8 (72.4 , 501)** | **48.8 (13.1 , 105.7)** |
| **Yemen** | **1316 (311 , 3127)** | **0.8 (0.2 , 1.9)** | **26.9 (6.4 , 63.2)** | **7011 (1784 , 16125)** | **1.7 (0.4 , 3.6)** | **52.8 (13.4 , 119.9)** | **96.2 (48.7 , 171.3)** |
| **South Asia** | **184683 (47051 , 397904)** | **1 (0.3 , 2.2)** | **32.5 (8.4 , 69.7)** | **931637 (256236 , 1937835)** | **2.5 (0.7 , 5.1)** | **65.2 (18 , 135.2)** | **100.5 (69.7 , 142.2)** |
| **Bangladesh** | **13079 (3068 , 29459)** | **0.7 (0.2 , 1.6)** | **27.6 (6.4 , 62.2)** | **51184 (12003 , 119204)** | **1.7 (0.4 , 3.5)** | **38.8 (9.2 , 90.4)** | **40.4 (3.6 , 90.5)** |
| **Bhutan** | **56 (14 , 132)** | **0.7 (0.2 , 1.7)** | **22.3 (5.5 , 52.1)** | **267 (68 , 596)** | **2 (0.5 , 4.2)** | **48 (12.3 , 106.7)** | **114.9 (59.1 , 202)** |
| **India** | **135760 (34833 , 294089)** | **1 (0.3 , 2.2)** | **30 (7.8 , 64.4)** | **710033 (193950 , 1495634)** | **2.6 (0.7 , 5.3)** | **61.5 (16.8 , 128.9)** | **105.1 (71.2 , 151.2)** |
| **Nepal** | **2161 (502 , 5093)** | **0.6 (0.1 , 1.5)** | **22.4 (5.2 , 53.9)** | **12096 (2920 , 27211)** | **1.9 (0.5 , 4.1)** | **53.6 (13 , 119.2)** | **139.4 (71.2 , 234)** |
| **Pakistan** | **33627 (8263 , 75165)** | **1.4 (0.3 , 3.1)** | **58.1 (14.4 , 128.9)** | **158057 (40356 , 346915)** | **2.5 (0.7 , 5.3)** | **136.5 (35.4 , 295.5)** | **135.1 (82.9 , 221)** |
| **Southern Sub-Saharan Africa** | **18240 (4641 , 39735)** | **1.7 (0.4 , 3.5)** | **68.7 (17.6 , 149)** | **63361 (17503 , 129140)** | **3.1 (0.9 , 6.2)** | **114.9 (32.2 , 232.2)** | **67.4 (44.4 , 96.6)** |
| **Botswana** | **351 (87 , 801)** | **1.6 (0.4 , 3.4)** | **63.7 (16.1 , 143.1)** | **1872 (479 , 4136)** | **3 (0.8 , 6.2)** | **142.8 (37.9 , 310.4)** | **124.2 (59.8 , 217.9)** |
| **Lesotho** | **378 (95 , 851)** | **1.2 (0.3 , 2.7)** | **39.5 (10.1 , 88.3)** | **1423 (356 , 3285)** | **2.2 (0.6 , 4.5)** | **113.1 (28.7 , 257.9)** | **186.5 (90.3 , 316.1)** |
| **Namibia** | **263 (69 , 580)** | **1.4 (0.4 , 3.1)** | **36.5 (9.6 , 80)** | **1009 (257 , 2292)** | **2.2 (0.6 , 4.5)** | **73.2 (18.9 , 162.7)** | **100.3 (51.4 , 176.1)** |
| **South Africa** | **14496 (3608 , 32213)** | **1.7 (0.4 , 3.7)** | **71.5 (17.9 , 159.9)** | **48948 (13426 , 100110)** | **3.3 (0.9 , 6.7)** | **111.6 (30.6 , 227.1)** | **56.1 (33.3 , 84.7)** |
| **Eswatini** | **183 (47 , 407)** | **1.4 (0.4 , 3.1)** | **66.8 (17.5 , 146.7)** | **744 (190 , 1731)** | **2.5 (0.7 , 5.2)** | **133 (34.4 , 306.1)** | **98.9 (44 , 179.4)** |
| **Zimbabwe** | **2570 (695 , 5450)** | **1.5 (0.4 , 3.1)** | **66.9 (18.5 , 140.8)** | **9364 (2510 , 20238)** | **2.5 (0.7 , 5.1)** | **137.3 (37.1 , 292.7)** | **105.3 (57.3 , 173)** |
| **Western Sub-Saharan Africa** | **22899 (5963 , 50630)** | **0.9 (0.2 , 1.8)** | **27.6 (7.3 , 60.1)** | **92015 (24253 , 198384)** | **1.5 (0.4 , 3.1)** | **52.5 (14.2 , 112.3)** | **90.2 (60 , 125.5)** |
| **Benin** | **605 (156 , 1337)** | **0.9 (0.2 , 1.9)** | **31.6 (8.2 , 70.2)** | **2615 (680 , 5734)** | **1.5 (0.4 , 3.2)** | **57.6 (15.3 , 125)** | **82 (44.1 , 138.9)** |
| **Burkina Faso** | **1321 (332 , 2924)** | **0.9 (0.2 , 2.1)** | **31.9 (8.1 , 70.4)** | **4548 (1157 , 9982)** | **1.3 (0.4 , 2.8)** | **52.9 (13.9 , 114.1)** | **66 (34.9 , 107.9)** |
| **Cameroon** | **1455 (379 , 3246)** | **1 (0.3 , 2)** | **35.1 (9.1 , 77.9)** | **8081 (2043 , 18466)** | **1.8 (0.5 , 3.8)** | **73.3 (18.6 , 164.8)** | **108.9 (58.9 , 180.5)** |
| **Cabo Verde** | **62 (16 , 132)** | **0.9 (0.2 , 2)** | **26.7 (6.8 , 56.9)** | **411 (113 , 871)** | **2.4 (0.7 , 5)** | **102.7 (28.4 , 217.2)** | **284.7 (195.9 , 396.5)** |
| **Chad** | **688 (175 , 1521)** | **0.8 (0.2 , 1.8)** | **25 (6.4 , 55.1)** | **2612 (646 , 5891)** | **1.3 (0.3 , 2.7)** | **49.8 (12.4 , 111.3)** | **99.6 (58 , 156.7)** |
| **CÃ´te d'Ivoire** | **1301 (330 , 2917)** | **0.8 (0.2 , 1.8)** | **36.5 (9.3 , 80)** | **6121 (1578 , 13492)** | **1.6 (0.4 , 3.5)** | **63.9 (16.8 , 139.3)** | **74.9 (39 , 126.4)** |
| **Gambia** | **64 (16 , 147)** | **0.6 (0.1 , 1.2)** | **19.4 (4.9 , 44.4)** | **430 (111 , 943)** | **1.3 (0.3 , 2.7)** | **46.9 (12.1 , 102.4)** | **142.2 (72.8 , 234)** |
| **Ghana** | **2361 (601 , 5333)** | **0.9 (0.2 , 2)** | **39.3 (10.3 , 87.8)** | **12833 (3358 , 28284)** | **2.2 (0.6 , 4.7)** | **82.8 (22 , 178.9)** | **110.6 (56.7 , 174.6)** |
| **Guinea** | **917 (230 , 2041)** | **0.6 (0.2 , 1.4)** | **28.5 (7.1 , 63.1)** | **2991 (767 , 6570)** | **1.1 (0.3 , 2.4)** | **56.7 (14.7 , 124)** | **98.9 (53.9 , 160.6)** |
| **Guinea-Bissau** | **167 (40 , 401)** | **0.8 (0.2 , 1.8)** | **42.2 (10.2 , 99.8)** | **478 (120 , 1109)** | **1.5 (0.4 , 3.1)** | **69 (17.6 , 157.5)** | **63.6 (22.9 , 122.2)** |
| **Liberia** | **445 (115 , 993)** | **1.2 (0.3 , 2.7)** | **40.8 (10.6 , 91)** | **1203 (305 , 2739)** | **1.9 (0.5 , 4)** | **63.9 (16.4 , 142.3)** | **56.5 (17.8 , 115)** |
| **Mali** | **1241 (303 , 2744)** | **0.8 (0.2 , 1.8)** | **31.3 (7.7 , 68.6)** | **4468 (1172 , 9842)** | **1.5 (0.4 , 3.1)** | **54.9 (14.7 , 119.6)** | **75.2 (39 , 122.3)** |
| **Mauritania** | **288 (73 , 638)** | **0.8 (0.2 , 1.8)** | **29.6 (7.5 , 65.2)** | **878 (218 , 1964)** | **1.6 (0.4 , 3.3)** | **44.3 (11.1 , 98.8)** | **49.9 (13 , 102)** |
| **Niger** | **323 (76 , 736)** | **0.3 (0.1 , 0.8)** | **12.8 (3.1 , 29.1)** | **1975 (482 , 4565)** | **0.8 (0.2 , 1.8)** | **27.6 (6.8 , 63.1)** | **115.2 (68.7 , 177)** |
| **Nigeria** | **9545 (2376 , 21731)** | **0.9 (0.2 , 1.9)** | **23 (5.8 , 52.1)** | **33739 (8206 , 74826)** | **1.3 (0.3 , 2.8)** | **42 (10.6 , 92.1)** | **82.3 (34.6 , 138.5)** |
| **Sao Tome and Principe** | **26 (7 , 57)** | **1.1 (0.3 , 2.4)** | **42.3 (10.5 , 91.4)** | **91 (24 , 202)** | **2 (0.5 , 4.4)** | **93.5 (24.5 , 204.1)** | **120.8 (73.3 , 182.3)** |
| **Senegal** | **1503 (388 , 3246)** | **1.4 (0.4 , 2.9)** | **48.8 (12.7 , 105.8)** | **5913 (1564 , 13015)** | **2.5 (0.7 , 5.1)** | **82.5 (21.9 , 181.6)** | **69.3 (31.5 , 126)** |
| **Sierra Leone** | **305 (76 , 700)** | **0.5 (0.1 , 1.2)** | **16.5 (4.1 , 38)** | **1180 (282 , 2654)** | **1 (0.2 , 2.1)** | **34.9 (8.4 , 77.7)** | **111.1 (57.9 , 185.8)** |
| **Togo** | **282 (72 , 638)** | **0.6 (0.2 , 1.3)** | **24.6 (6.4 , 54.7)** | **1448 (354 , 3270)** | **1.2 (0.3 , 2.6)** | **42.9 (10.7 , 95.8)** | **74.5 (32.9 , 127)** |
| **Eastern Sub-Saharan Africa** | **19316 (5036 , 42230)** | **0.5 (0.1 , 1.1)** | **26.6 (7 , 58.5)** | **62092 (16026 , 134535)** | **0.9 (0.3 , 2)** | **39.7 (10.4 , 84.8)** | **49 (30.9 , 72.2)** |
| **Burundi** | **747 (188 , 1680)** | **0.6 (0.1 , 1.3)** | **32.3 (8.2 , 72.2)** | **1522 (367 , 3623)** | **0.8 (0.2 , 1.7)** | **34.4 (8.4 , 81.3)** | **6.6 (-25.3 , 49.9)** |
| **Comoros** | **55 (14 , 129)** | **0.7 (0.2 , 1.6)** | **25.5 (6.4 , 59.5)** | **183 (46 , 406)** | **1.2 (0.3 , 2.5)** | **38.4 (9.7 , 84.3)** | **50.3 (10.6 , 135.7)** |
| **Djibouti** | **38 (9 , 89)** | **0.5 (0.1 , 1.2)** | **29.8 (7.4 , 67.9)** | **301 (73 , 728)** | **1.2 (0.3 , 2.5)** | **53.3 (13.5 , 125.3)** | **79 (34.3 , 139.2)** |
| **Eritrea** | **259 (60 , 604)** | **0.5 (0.1 , 1.1)** | **26.2 (6.4 , 61)** | **1359 (324 , 3054)** | **1 (0.3 , 2.3)** | **51.5 (12.3 , 113.5)** | **96.7 (44.6 , 177.3)** |
| **Ethiopia** | **5925 (1451 , 13651)** | **0.5 (0.1 , 1.3)** | **29.7 (7.3 , 68)** | **10839 (2617 , 24589)** | **0.8 (0.2 , 1.7)** | **27.4 (6.7 , 61.9)** | **-7.6 (-27.3 , 25.3)** |
| **Kenya** | **1272 (310 , 2949)** | **0.5 (0.1 , 1.2)** | **16.2 (4 , 37.5)** | **7191 (1803 , 16296)** | **1 (0.3 , 2.1)** | **33.3 (8.5 , 74.5)** | **106.4 (70.8 , 157.5)** |
| **Madagascar** | **1165 (293 , 2543)** | **0.5 (0.1 , 1.1)** | **23.2 (5.9 , 50.5)** | **3546 (871 , 8566)** | **0.9 (0.2 , 2)** | **32.8 (8.2 , 78.2)** | **41.4 (5.5 , 85.8)** |
| **Malawi** | **1153 (297 , 2521)** | **0.4 (0.1 , 1)** | **31.8 (8.3 , 68.7)** | **3408 (891 , 7293)** | **0.9 (0.2 , 2)** | **49.6 (13.1 , 105.4)** | **56.3 (25.9 , 96.2)** |
| **Mozambique** | **1304 (334 , 2873)** | **0.6 (0.1 , 1.2)** | **23.4 (6 , 51.4)** | **5357 (1379 , 12226)** | **1.1 (0.3 , 2.4)** | **51.4 (13.2 , 115.6)** | **119.4 (62.4 , 195)** |
| **Rwanda** | **1035 (262 , 2352)** | **0.6 (0.1 , 1.4)** | **36.2 (9.3 , 80.2)** | **2497 (611 , 5652)** | **1.1 (0.3 , 2.3)** | **43.4 (10.8 , 96.5)** | **20 (-10.2 , 67.4)** |
| **Somalia** | **581 (139 , 1380)** | **0.4 (0.1 , 1)** | **23.9 (5.8 , 56.8)** | **2050 (457 , 5279)** | **0.7 (0.2 , 1.5)** | **31.4 (7.2 , 80.7)** | **31.4 (-8.7 , 84)** |
| **South Sudan** | **700 (165 , 1667)** | **0.6 (0.2 , 1.5)** | **30.9 (7.4 , 72.9)** | **1462 (358 , 3437)** | **1 (0.3 , 2.2)** | **40.7 (10.2 , 93.7)** | **31.8 (-2.4 , 80.4)** |
| **United Republic of Tanzania** | **2109 (525 , 4822)** | **0.4 (0.1 , 0.9)** | **20.6 (5.2 , 46.3)** | **9698 (2445 , 21392)** | **0.9 (0.2 , 1.9)** | **41.4 (10.6 , 90.4)** | **100.7 (64 , 143.2)** |
| **Uganda** | **1896 (501 , 4213)** | **0.7 (0.2 , 1.5)** | **30.4 (8 , 66.6)** | **8843 (2262 , 19406)** | **1.2 (0.3 , 2.5)** | **64.6 (16.9 , 140)** | **112.6 (65.9 , 182.9)** |
| **Zambia** | **1063 (272 , 2343)** | **0.6 (0.1 , 1.3)** | **38.8 (10 , 86.2)** | **3785 (962 , 8403)** | **1.1 (0.3 , 2.3)** | **59.2 (15.3 , 130.5)** | **52.3 (19.4 , 99.5)** |
| **Central Sub-Saharan Africa** | **11937 (2860 , 30549)** | **1.2 (0.3 , 2.8)** | **53.7 (12.9 , 134.7)** | **37203 (8966 , 86838)** | **2 (0.5 , 4.3)** | **70.9 (17.5 , 162.3)** | **32.1 (2.4 , 71.6)** |
| **Angola** | **1990 (462 , 4691)** | **1.1 (0.3 , 2.4)** | **51.7 (12.2 , 119.6)** | **8243 (2055 , 18779)** | **1.9 (0.5 , 4)** | **74.8 (19.1 , 166.2)** | **44.5 (9.5 , 102.1)** |
| **Central African Republic** | **671 (150 , 1687)** | **1.1 (0.3 , 2.7)** | **55.9 (13.3 , 136.8)** | **1588 (356 , 4194)** | **1.7 (0.4 , 3.8)** | **70.2 (16.5 , 180.4)** | **25.5 (-4.4 , 62.5)** |
| **Congo** | **848 (198 , 2009)** | **1.6 (0.4 , 3.4)** | **78.9 (18.9 , 182.8)** | **2638 (664 , 6021)** | **2.5 (0.7 , 5.4)** | **101.5 (25.9 , 226.9)** | **28.6 (-1.3 , 69.1)** |
| **Democratic Republic of the Congo** | **7845 (1823 , 20997)** | **1.2 (0.3 , 3)** | **51.1 (11.9 , 137.1)** | **22845 (5125 , 56175)** | **1.9 (0.5 , 4.5)** | **64.9 (14.9 , 158.3)** | **27.1 (-7.4 , 71.8)** |
| **Equatorial Guinea** | **96 (22 , 234)** | **1.2 (0.3 , 2.7)** | **47.7 (11.2 , 115.2)** | **494 (119 , 1212)** | **2.7 (0.7 , 5.8)** | **108.2 (26.6 , 258.4)** | **126.8 (42.5 , 256.2)** |
| **Gabon** | **488 (117 , 1166)** | **2 (0.5 , 4.5)** | **87.1 (21.2 , 205.8)** | **1394 (347 , 3180)** | **3.4 (0.9 , 7.2)** | **135.3 (34 , 304.5)** | **55.4 (17.4 , 98.5)** |
