# Supplementary figures and images for: Global, Regional and National Burden of Cancers Attributable to High Fasting Plasma Glucose in 204 Countries and Territories, 1990-2019
Source: Front Endocrinol (Lausanne). 2022 Jul 19;13:879890. doi: 10.3389/fendo.2022.879890 (PMC9366927; doi:10.3389/fendo.2022.879890)

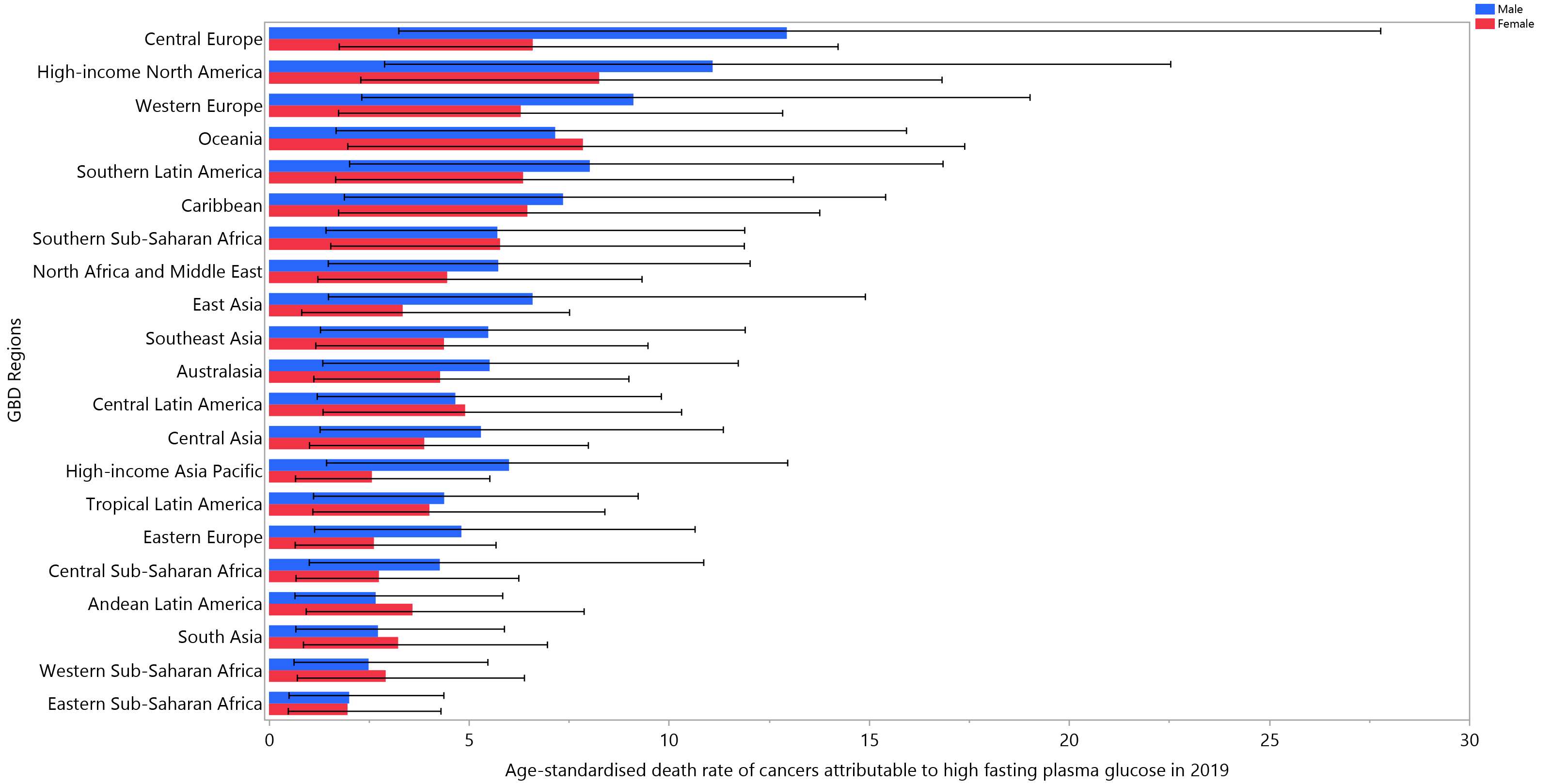

Supplement: Supplementary Figure 1 — The age-standardized death rate of cancers attributable to high fasting plasma glucose in 2019 for the 21 Global Burden of Disease regions, by sex. (Generated from data available from http://ghdx.healthdata.org/gbd-results-tool). [file Image_1.tif]

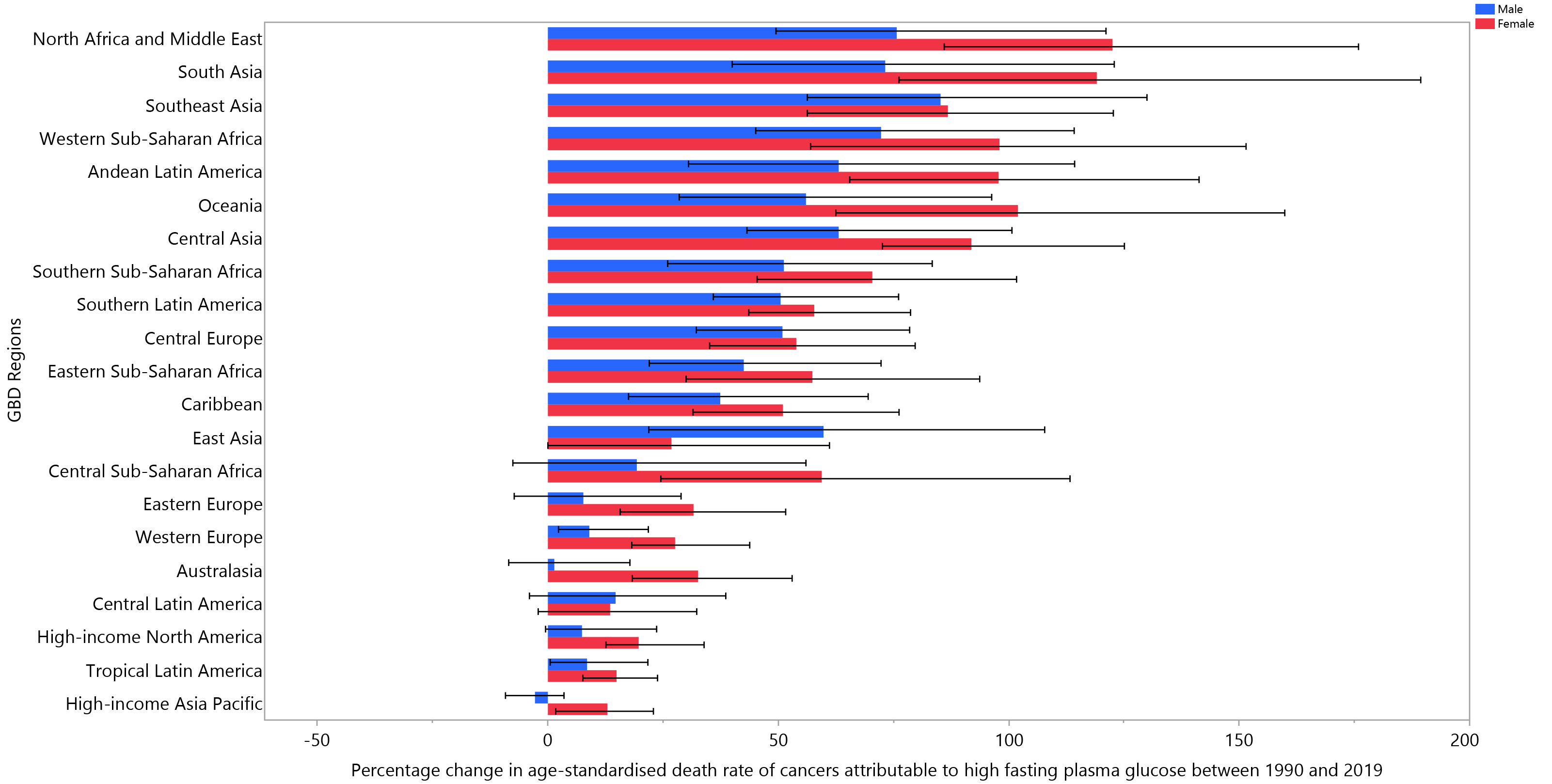

Supplement: Supplementary Figure 2 — The percentage change in the age-standardized death rate of cancers attributable to high fasting plasma glucose from 1990 to 2019 for the 21 Global Burden of Disease regions, by sex. (Generated from data available from http://ghdx.healthdata.org/gbd-results-tool). [file Image_2.tif]

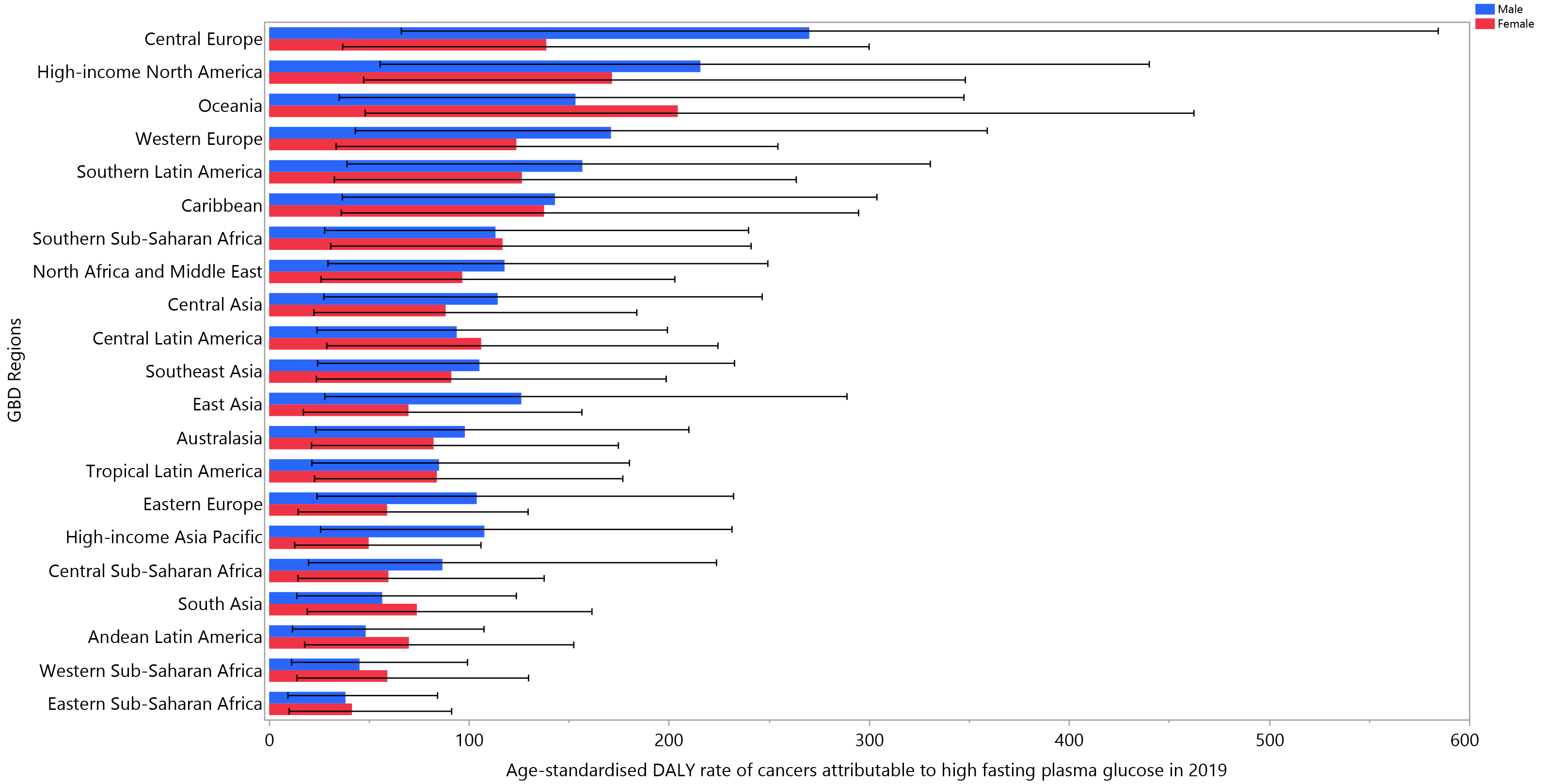

Supplement: Supplementary Figure 3 — The age-standardized DALY rate of cancers attributable to high fasting plasma glucose in 2019 for the 21 Global Burden of Disease regions, by sex. DALY= disability-adjusted-life-years. (Generated from data available from http://ghdx.healthdata.org/gbd-results-tool). [file Image_3.tif]

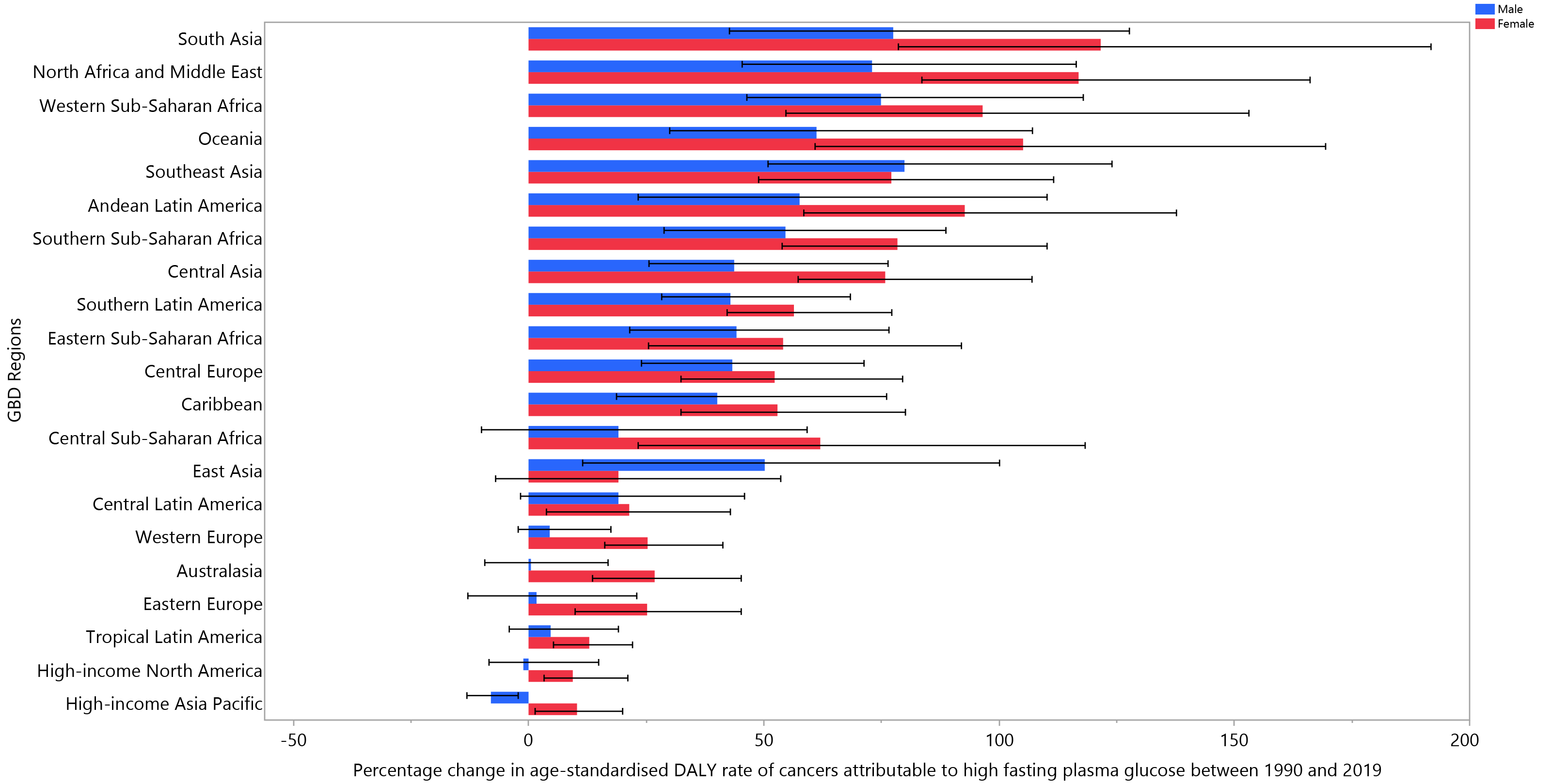

Supplement: Supplementary Figure 4 — The percentage change in the age-standardized DALY rate of cancers attributable to high fasting plasma glucose from 1990 to 2019 for the 21 Global Burden of Disease regions, by sex. DALY=disability-adjusted-life-years. (Generated from data available from http://ghdx.healthdata.org/gbd-results-tool). [file Image_4.tif]

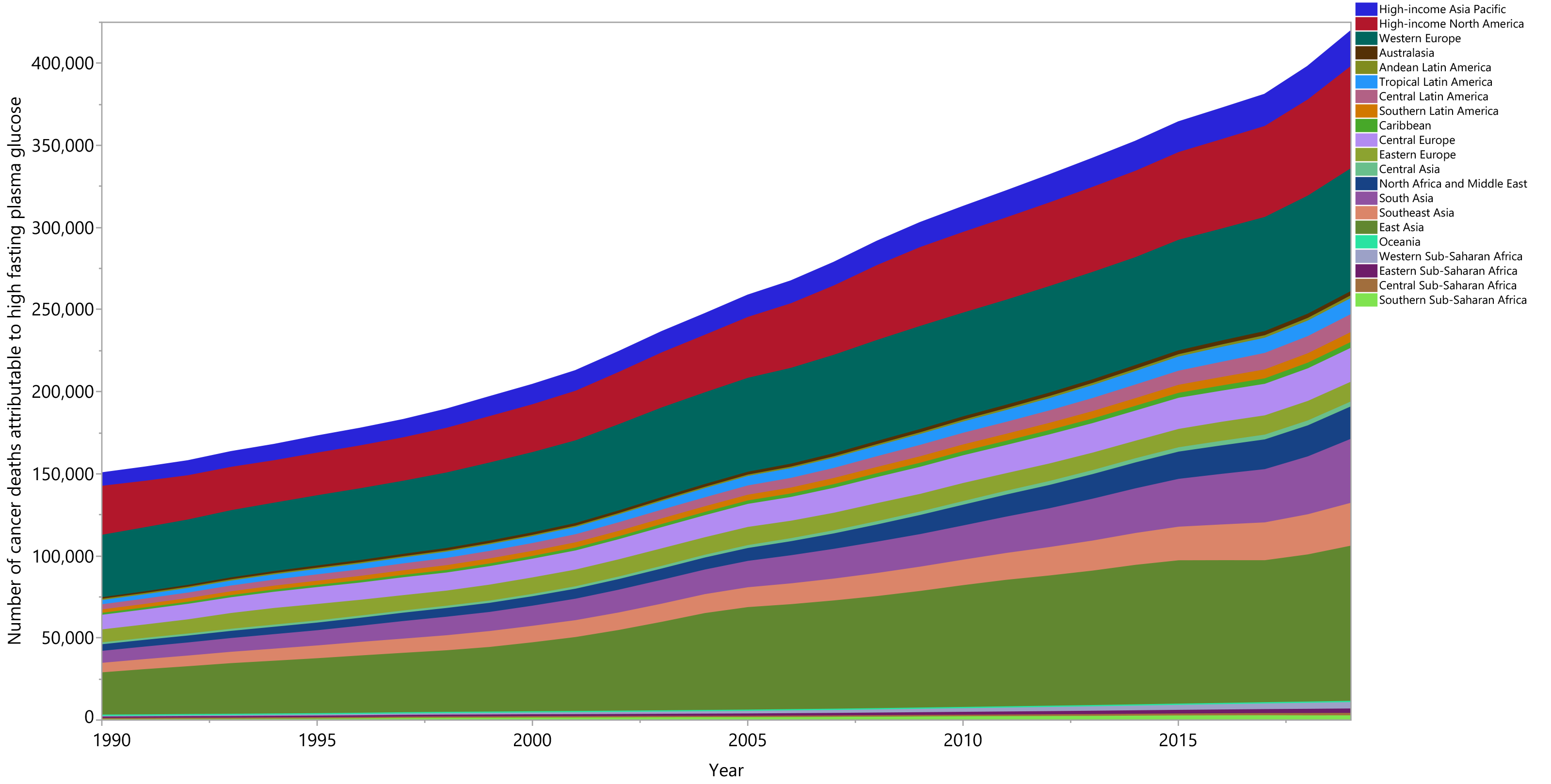

Supplement: Supplementary Figure 5 — Number of cancer deaths attributable to high fasting plasma glucose from 1990 to 2019 for the 21 Global Burden of Disease regions. (Generated from data available from http://ghdx.healthdata.org/gbd-results-tool). [file Image_5.tif]

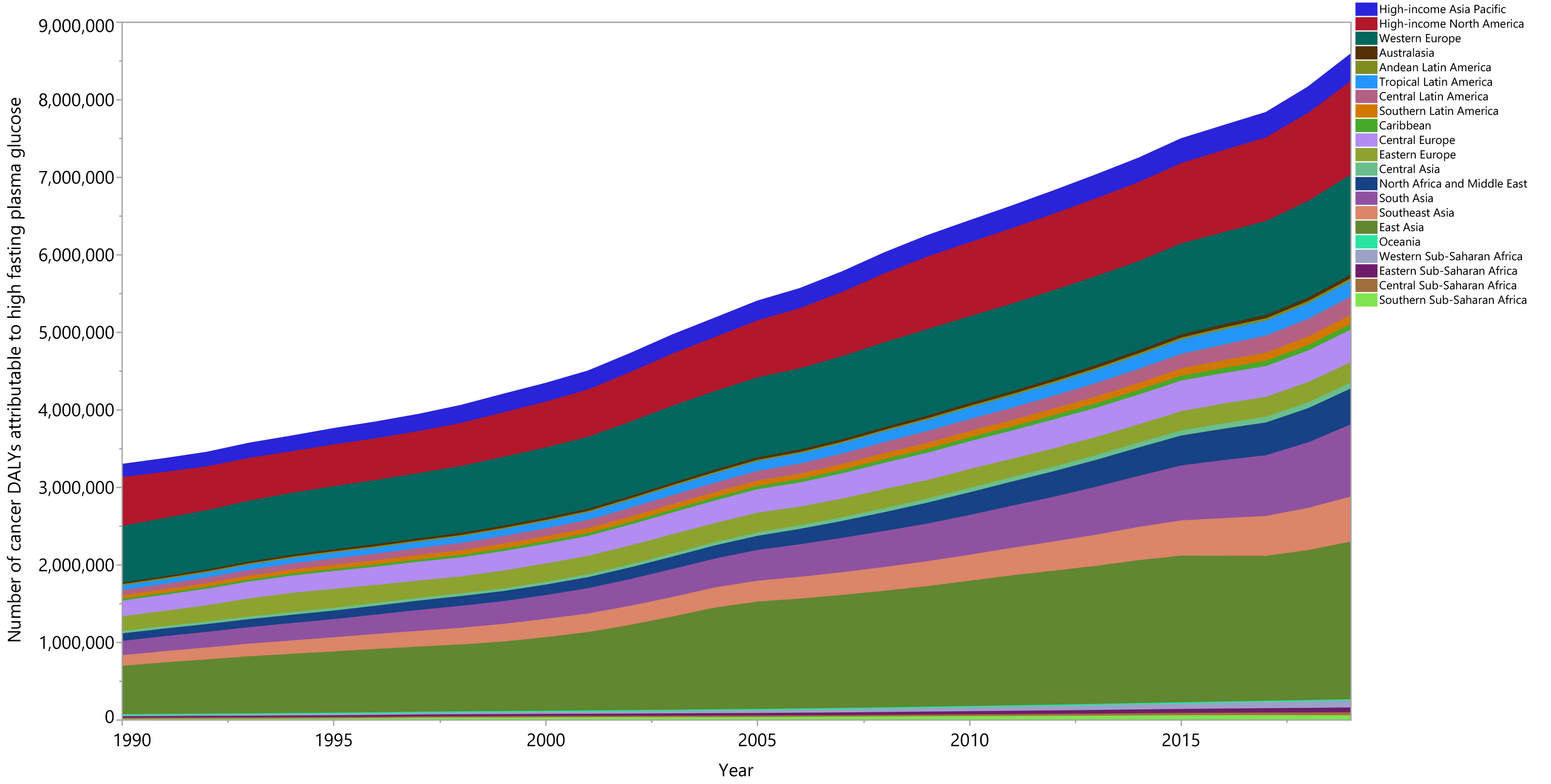

Supplement: Supplementary Figure 6 — Number of cancer DALYs attributable to high fasting plasma glucose from 1990 to 2019 for the 21 Global Burden of Disease regions. DALY=disability-adjusted-life-years. (Generated from data available from http://ghdx.healthdata.org/gbd-results-tool). [file Image_6.tif]

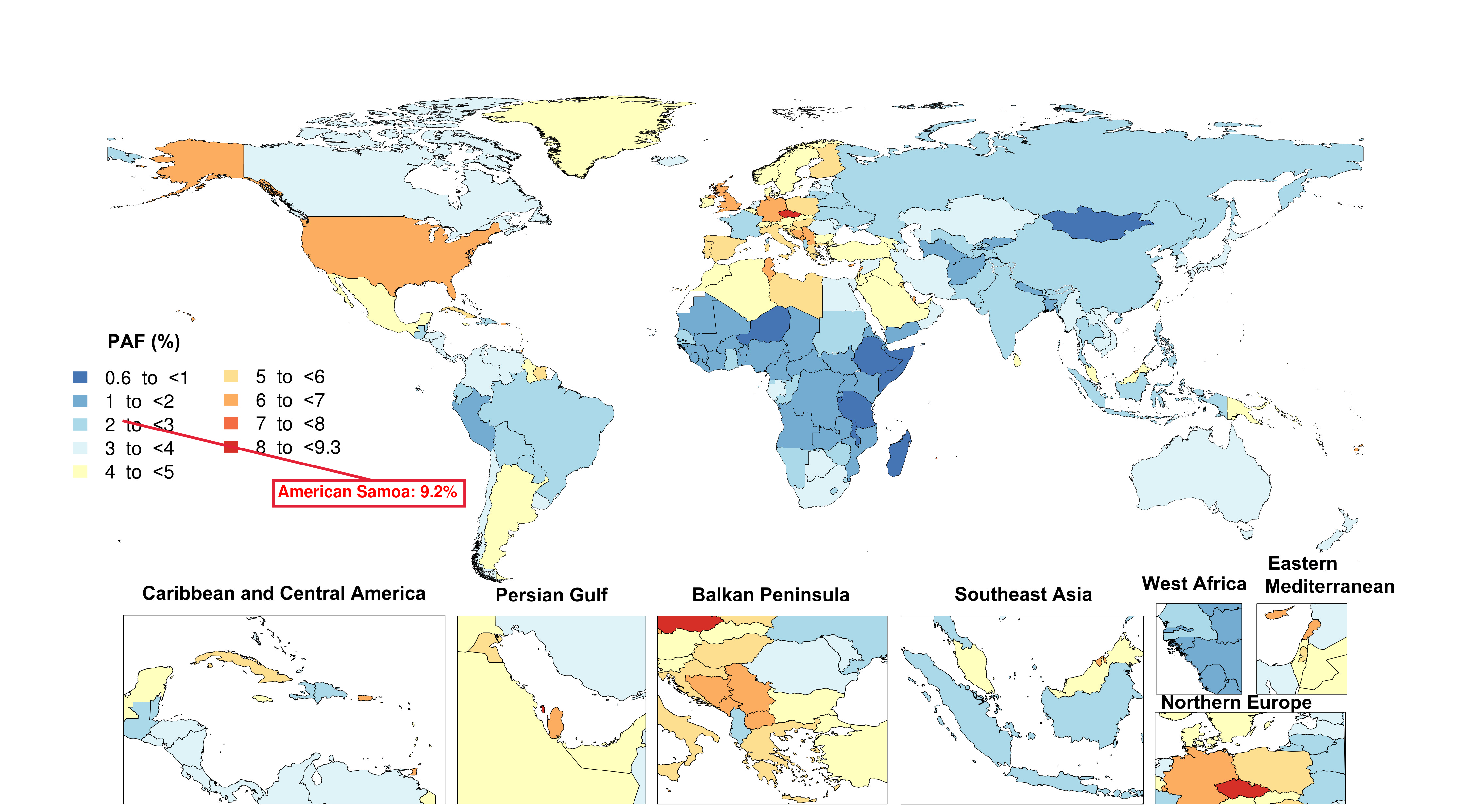

Supplement: Supplementary Figure 7 — Population attributable fraction (PAF) of DALYs due to cancers attributable to high fasting plasma glucose in 2019, by country. DALY=disability-adjusted-life-years. (Generated from data available from http://ghdx.healthdata.org/gbd-results-tool). [file Image_7.tif]

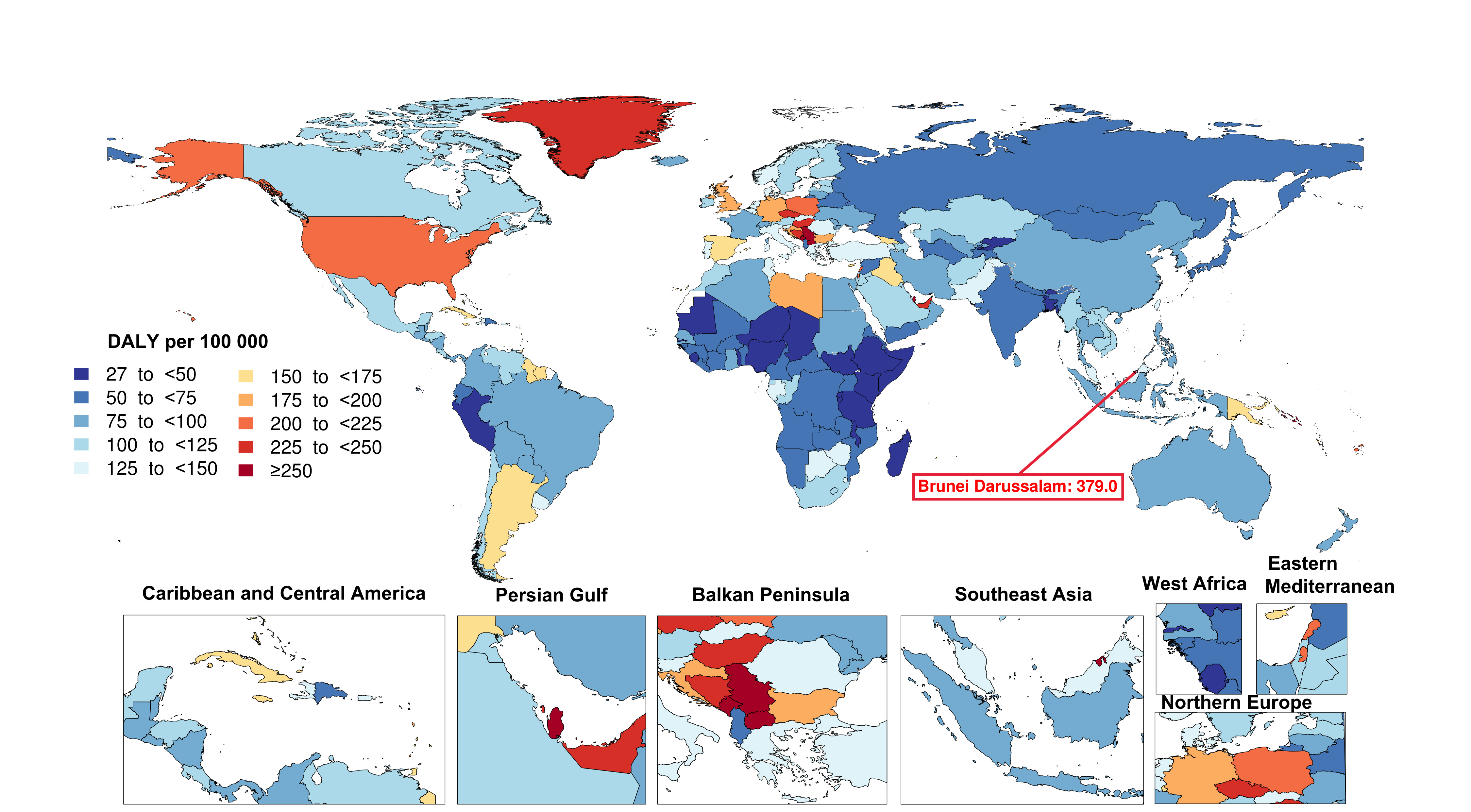

Supplement: Supplementary Figure 8 — Age-standardized rate of DALYs due to cancers that were attributable to high fasting plasma glucose in 2019, by country. (Generated from data available from http://ghdx.healthdata.org/gbd-results-tool). [file Image_8.tif]

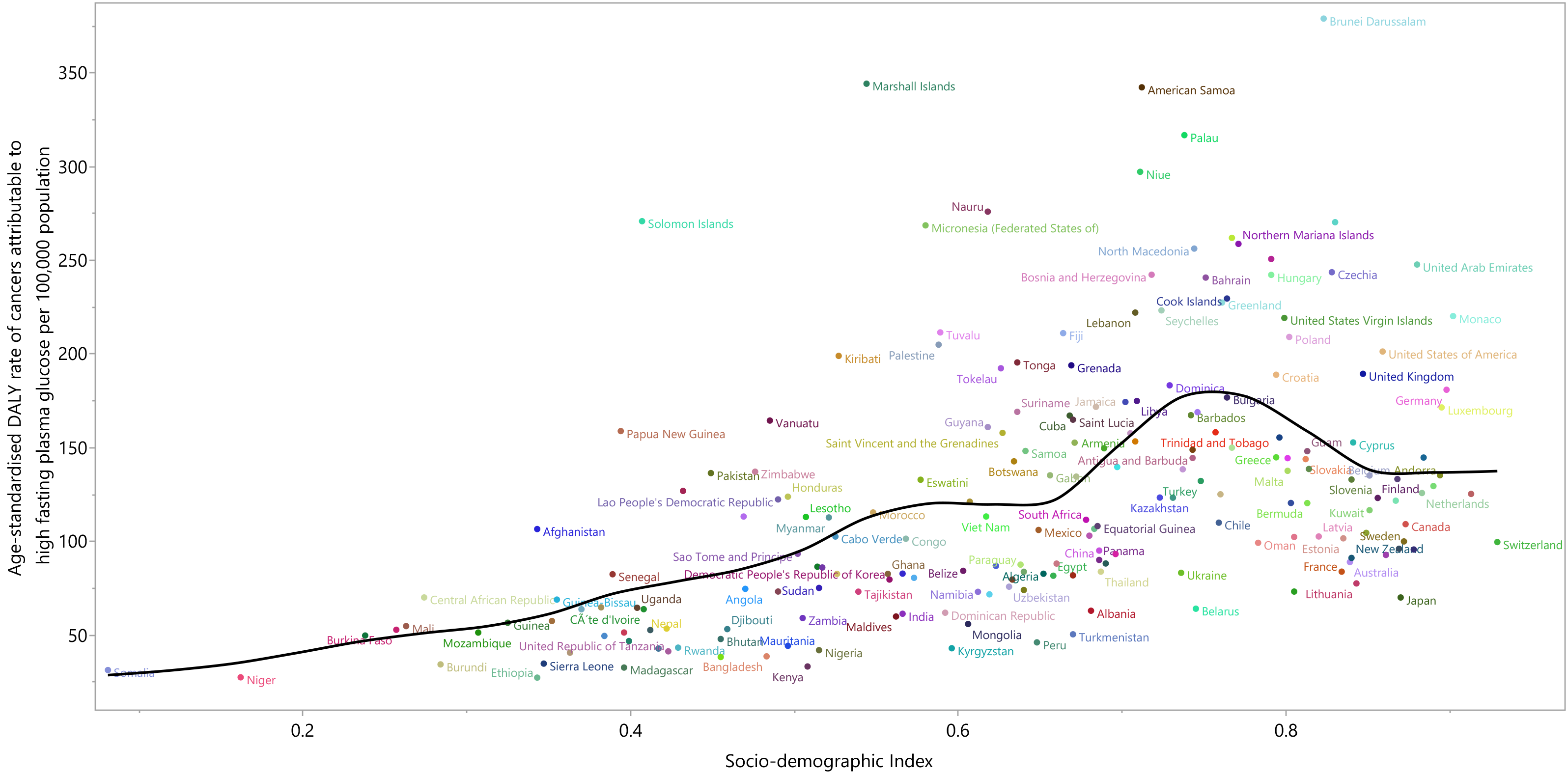

Supplement: Supplementary Figure 9 — Age-standardized DALY rates of cancers attributable to high fasting plasma glucose by Socio-demographic Index for 204 countries and territories in 2019; Expected values are shown as the black line. Each point shows observed age-standardized DALY rate for specified country in 2019. DALY=disability-adjusted-life-years. (Generated from data available from http://ghdx.healthdata.org/gbd-results-tool). [file Image_9.tif]
